# *Arabidopsis* epigenetic factor AS2 mitigates nucleolar stress and promotes flat leaf development by repressing a CDK inhibitor

**DOI:** 10.1242/dev.205275

**Published:** 2026-09-18

**Authors:** Ayami Nakagawa, Hidekazu Iwakawa, Hiro Takahashi, Simon Vial-Pradel, Mari Takahashi, Kazuomi Ohga, Takuma Ito, Motoki Tamai, Eri Takada, Sumie Keta, Nanako Ishibashi, Sayuri Ando, Iwai Ohbayashi, Masaki Ito, Mami Yamazaki, Ayato Sato, Masaaki Umeda, Byung-Yoon Cha, Je-Tae Woo, Michiko Sasabe, Munetaka Sugiyama, Shoko Kojima, Yasunori Machida, Chiyoko Machida

**Affiliations:** ^1^ Graduate School of Bioscience and Biotechnology, Chubu University, Kasugai 487-8501, Japan; ^2^ Institute of Transformative Bio-Molecules (WPI-ITbM), Nagoya University, Nagoya 464-8602, Japan; ^3^ Division of Biological Science and Technology, Graduate School of Natural Science and Technology, Kanazawa University Kakuma-machi, Kanazawa 920-1192, Japan; ^4^ Graduate School of Medical Sciences, Kanazawa University, Kakuma-machi, Kanazawa, Ishikawa 920-1192, Japan; ^5^ Division of Biological Science, Graduate School of Science, Nagoya University, Nagoya 464-8602, Japan; ^6^ Department of Life Sciences, National Cheng Kung University, Tainan City 701, Taiwan; ^7^ Graduate School of Pharmaceutical Sciences, Chiba University, Chiba 260-8675, Japan; ^8^ Graduate School of Science and Technology, Nara Institute of Science and Technology, Nara 630-0192, Japan; ^9^ Department of Biology, Faculty of Agriculture and Life Science, Hirosaki University, Bunkyo-cho, Hirosaki 036-8561, Japan; ^10^ Department of Biological Sciences, Graduate School of Science, The University of Tokyo, Tokyo 113-0033, Japan

**Keywords:** *ASYMMETRIC LEAVES2*, AS2 body, Camptothecin, CDK inhibitor *KRP5*/*ICK3*, Nucleolus, Topoisomerase I

## Abstract

The *Arabidopsis thaliana* leaf, which exhibits a symmetrically extended flat morphology, consists of two distinct cellular domains: adaxial and abaxial layers. The *ASYMMETRIC LEAVES2* (*AS2*) gene is essential for specifying the adaxial domain, and its protein forms nucleolar structures, termed AS2 bodies, at ribosomal-DNA loci. Numerous nucleolus-related genes have been reported to be cooperatively involved in leaf adaxialization together with *AS2*. However, the molecular relationships between *AS2* and these genes remain unclear. To identify chemical modulators of AS2 function, we screened a chemical library containing natural products and identified eight molecules, including camptothecin, that induced filamentous leaves in the *as2* mutant. Camptothecin is an inhibitor of topoisomerase I, which is required for transcription of ribosomal RNA genes, and induces nucleolar stress. Treatment with 10-hydroxy-camptothecin increased transcript levels of the CDK inhibitor *KIP*-*RELATED*-*PROTEIN5*/*INHIBITOR*-*OF*-*CYCLIN*-*DEPENDENT*-*KINASE3* (*KRP5/ICK3*), and caused notable changes in the number and morphology of AS2 bodies. Given its role in leaf morphogenesis, our findings suggest that AS2 is a key factor in leaf adaxialization, which leads to the formation of flat leaves, and in protection against nucleolar stress through coordination with nucleolar proteins.

## Teaser

**Highlighted Article:** Flattened leaf morphology is essential for efficient photosynthesis and ultimately for sustaining life on Earth. *Arabidopsis* AS2 plays a crucial role in developing flat leaves, even under nucleolar stress conditions.

## INTRODUCTION

Morphogenesis in multicellular organisms depends on coordinated cell division and differentiation (Harashima and Schnittger, 2010). Leaves arise from the shoot apical meristem (SAM) and establish three developmental axes to form flat and symmetric organs. In *Antirrhinum majus*, *PHANTASTICA* (*PHAN*) specifies adaxial identity required for lateral leaf expansion, and *ASYMMETRIC LEAVES1* (*AS1*) is its ortholog in *Arabidopsis thaliana* (Waites and Hudson, 1995; Waites et al., 1998; Hudson, 2000; Byrne et al., 2000). During early leaf development, adaxial fate is established in cells adjacent to the SAM, thereby generating adaxial–abaxial polarity; subsequent cell proliferation along the medial–lateral axis gives rise to a flat leaf blade (Byrne et al., 2000; Iwakawa et al., 2002; Bowman et al., 2002; Newman et al., 2002; Tsukaya, 2006; Fukushima and Hasebe, 2014). Although antagonistic interactions between adaxial and abaxial determinants are essential for leaf flattening, the mechanisms by which these factors regulate cell division remain unclear (Wu et al., 2008; Iwasaki et al., 2013; Fukushima and Hasebe, 2014; Machida et al., 2015; Husbands et al., 2015).

ASYMMETRIC LEAVES2 (AS2), a member of the plant-specific AS2/LOB family, forms a complex with the MYB-domain protein ASYMMETRIC LEAVES1 (AS1) (Byrne et al., 2000; Iwakawa et al., 2002; Guo et al., 2008; Yang et al., 2008). These proteins colocalize in the nucleoplasm and form subnucleolar structures termed AS2 bodies at the nucleolar periphery (Ueno et al., 2007; Luo et al., 2012, 2020). AS2 bodies partially overlap with chromocenters containing nucleolus organizer regions (NORs), which are composed of condensed heterochromatin derived from 45S ribosomal DNA repeats (Luo et al., 2020; Iwakawa et al., 2020; Ando et al., 2023). The AS2–AS1 complex directly represses class 1 *KNOX* genes and *ETTIN*/*AUXIN RESPONSE FACTOR3* (*ETT*/*ARF3*) and indirectly represses *ETT/ARF3* and *ARF4* through the miR390-tasiR-ARF pathway (Guo et al., 2008; Yang et al., 2008; Ikezaki et al., 2010; Iwasaki et al., 2013; Husbands et al., 2015). It also contributes to maintenance of gene-body DNA methylation in *ETT/ARF3*, the CpG methylation levels of which are inversely correlated with transcript accumulation (Iwasaki et al., 2013; Vial-Pradel et al., 2018). Repression of *ETT/ARF3* by AS2–AS1 is essential for adaxial–abaxial polarity establishment and flat leaf formation, and this repression is closely associated with AS2 body formation at the nucleolar periphery (Iwasaki et al., 2013; Takahashi et al., 2013; Matsumura et al., 2016; Luo et al., 2020; Ando et al., 2023).

Both *as2* and *as1* single mutants show mild defects in leaf adaxialization (Iwakawa et al., 2007), whereas additional mutations in these backgrounds often produce filamentous leaves that lack adaxial identity (Pinon et al., 2008; Yao et al., 2008; Szakonyi and Byrne, 2011; Horiguchi et al., 2011; Iwasaki et al., 2013; Machida et al., 2015). Genetic screens using *as2* or *as1* mutants have identified 60 modifier genes that function together with AS2–AS1 in leaf adaxialization (Machida et al., 2015; Iwakawa et al., 2020). These genes fall into several functional categories, including ribosomal and nucleolar components, factors involved in genome stability and chromatin organization, and components of the tasiR–ARF pathway. Many are linked to nucleolar function, such as *RNA HELICASE10* (*RH10*), *NUCLEOLIN1* (*NUC1*) and *ROOT INITIATION DEFECTIVE2* (*RID2*) (Ohbayashi et al., 2011, 2017; Matsumura et al., 2016; Ohbayashi and Sugiyama, 2018; Iwakawa et al., 2020), and mutations in these genes alter the pattern and localization of AS2 bodies (Luo et al., 2020; Ando et al., 2023). Because disruption of ribosome biogenesis causes nucleolar stress, and a plant-specific nucleolar stress response pathway has recently been identified (Ohbayashi et al., 2017; Ohbayashi and Sugiyama, 2018), the molecular relationship between AS2–AS1, modifier proteins and nucleolar stress is an important unresolved issue.

To investigate the functional relationship between AS2–AS1-associated modifiers and leaf development, we carried out a chemical screen using a natural product library. Natural products include many inhibitors of ribosome function and anticancer compounds that preferentially affect rapidly dividing cells (Boulon et al., 2010; Burger et al., 2010). This screen identified eight compounds that induced filamentous leaf formation in *as2* and *as1* mutants. One of these compounds was camptothecin (CPT), a well-known isoquinoline alkaloid and specific inhibitor of topoisomerase I (TOPI) (Hsiang et al., 1985, 1989). Because CPT can be applied directly to plants, it provides a useful tool for rapid analysis of leaf morphology and gene expression. Notably, nucleolar TOPI is required for transcription of ribosomal RNA (rRNA) genes (Ishihara et al., 2022), suggesting a possible link between nucleolar function, modifier activity and leaf development.

In this study, we demonstrate that *Arabidopsis TOP1α* cooperates with AS2–AS1 in leaf adaxialization. Meta-analysis of microarray and single-cell RNA-sequencing (scRNA-seq) data together with genetic analyses showed that repression of *KRP5/ICK3*, which encodes a Cip/Kip family CDK inhibitor, is essential for adaxialization and flat leaf formation. We also found that the *top1α* mutation and 10-hydroxycamptothecin (10H-CPT) treatment disrupted the morphology and patterning of AS2 bodies in the nucleolus, resembling the defects seen in *rh10*, *nuc1* and *rid2* mutants under nucleolar stress (Ando et al., 2023). Moreover, mutation of *SUPPRESSOR OF ROOT INITIATION DEFECTIVE TWO1* (*SRIW1*)/*ANAC082*, a nucleolar stress-response factor, suppressed the abaxialized filamentous-leaf phenotype of *as2 top1α* plants. We therefore propose that AS2 not only regulates leaf polarity but also attenuates nucleolar stress, thereby supporting flat leaf development under stress conditions.

## RESULTS

### Camptothecin induces filamentous leaf formation in the *as2* mutant

To elucidate the molecular network of leaf formation controlled by the *AS2* and *AS1* genes, 2209 molecules containing natural compounds derived from Inter BioScreen, which are composed of micro-organism and plant-derived natural compounds and their derivatives, were screened. Based on previous studies (Nakagawa et al., 2012; Iwasaki et al., 2013; Luong et al., 2018), in the first screening, Col-0 (wild type), *as2-1* and *as1-1* plants were grown for 3 weeks at a concentration of 12.5 μM, and 35 molecules that showed growth inhibition were selected. In the second screening, 35 molecules were tested at various concentrations, and eight molecules were identified that produced filamentous leaves in *as2-1* and *as1-1* (Fig. 1A; Table 1).

**Fig. 1.**
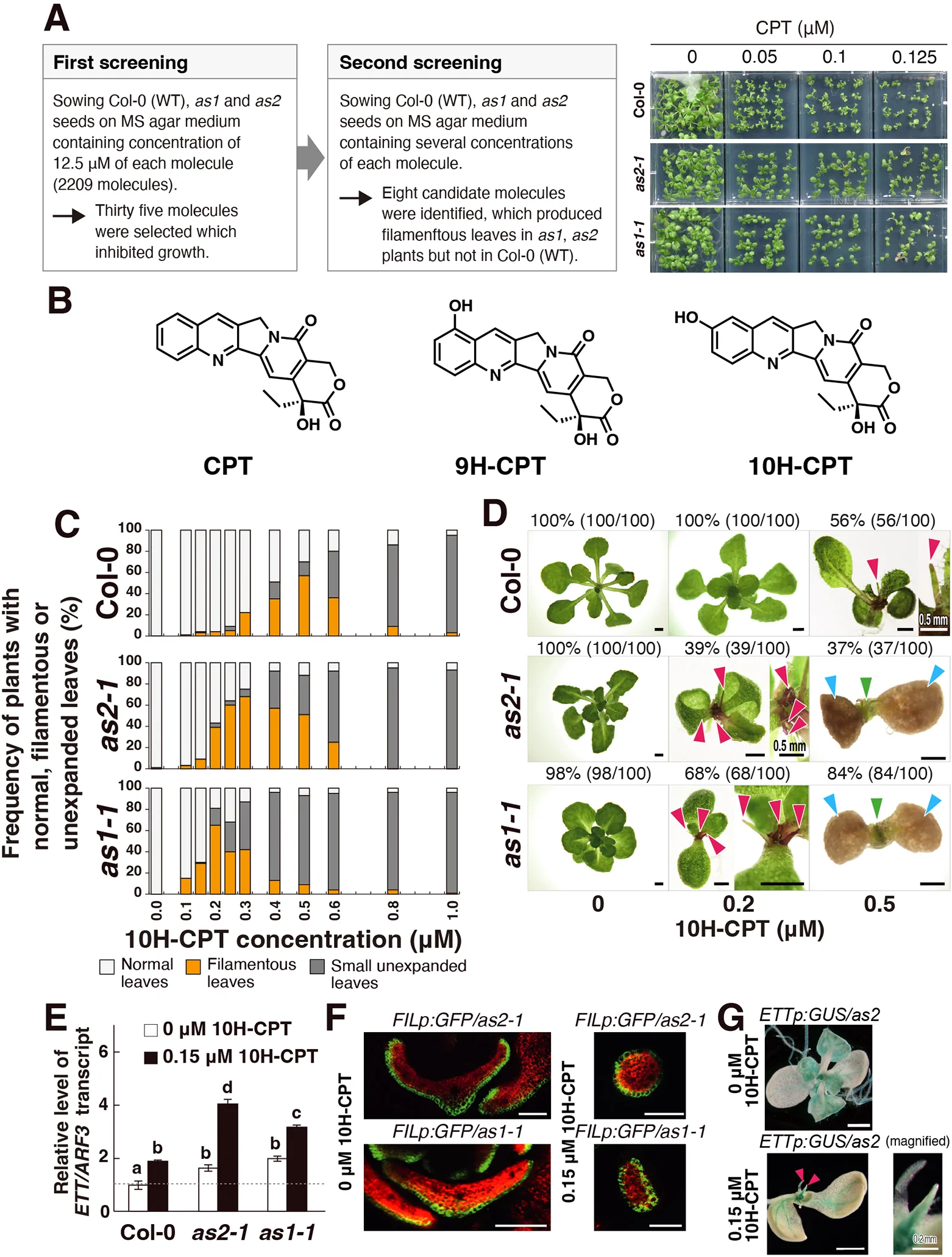
**Procedures used for screening molecules that produced filamentous leaves in *as2-1* and *as1-1* mutants, and characterization of the phenotypes of plants treated with 10-hydroxycamptothecin (10H-CPT).** (A) Left and middle: experimental procedures for two-step screenings of molecules that induced filamentous leaves in *as2-1* and *as1-1* mutants. Right: dose-dependent effects of 10H-CPT on leaf abnormalities and leaf formation. Col-0, *as2-1* or *as1-1* plants treated with CPT for 17 days after sowing. (B) Chemical structures of CPT, 9H-CPT and 10H-CPT. (C) Dose-dependent effects of 10H-CPT on leaf abnormalities and leaf formation. Each value is calculated from the mean of three biological replicates (*n*=100 plants). (D) Phenotypes of Col-0, *as2-1* and *as1-1* plants in the presence of DMSO, and 0.2 and 0.5 μM 10H-CPT for 21 days after sowing. Scale bars: 1 mm. Red arrowheads indicate filamentous leaves. Green arrowheads indicate small unexpanded leaves like clumps. Blue arrowheads indicate withered cotyledons. Values above each image indicate the frequency (%) of each leaf phenotype at each concentration (0, 0.2 or 0.5 μM). Numbers in parentheses represent the number of seedlings with the indicated phenotype/total number of seedlings examined. (E) Changes (mean±s.d.) in the transcription levels of the *ETT*/*ARF3* gene of shoot apices in plants treated with 0 or 0.15 μM 10H-CPT for 14 days after sowing. Each value was normalized by reference to the level of *ACT2* transcripts. The values are shown relative to the value for Col-0 plants (dashed line) (*P*<0.05, Tukey's HSD test after significant ANOVA). (F) *FILp:GFP*/*as2-1* and *as1-1* plants treated with 0 μM or 0.15 μM 10H-CPT for 14 days after sowing. Scale bars: 100 μm. (G) An *ETTp:GUS*/*as2-1* plant treated with 0 μM or 0.15 μM 10H-CPT for 14 days after sowing. Scale bars: 1 mm (0.2 mm in magnified image in G).

**Table 1.** Molecules identified in the chemical library that induced filamentous leaves in *as2-1* and *as1-1* mutants

Identified molecule	Molecular function	CAS registration number	IUPAC name	Chemical structure
Camptothecin	Inhibitor of topoisomerase I	7689-03-4	1*H*-Pyrano[3′,4′:6,7]indolizino[1,2-*b*]quinoline-3,14(4*H*,12*H*)-dione, 4-ethyl-4-hydroxy-, (4*S*)- (9CI, ACI)	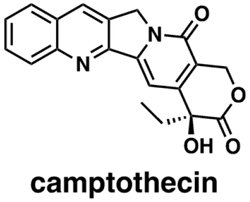
9-Hydroxy-camptothecin	Inhibitor of topoisomerase I	67656-30-8	1*H*-Pyrano[3′,4′:6,7]indolizino[1,2-b]quinoline-3,14(4H,12H)-dione, 4-ethyl-4,10-dihydroxy-, (4S)- (9CI, ACI)	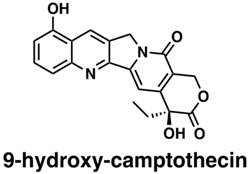
Bleomycin A5	Leading to double strand break	11116-32-8	Bleomycinamide, N1-[3-[(4-aminobutyl)amino]propyl]-	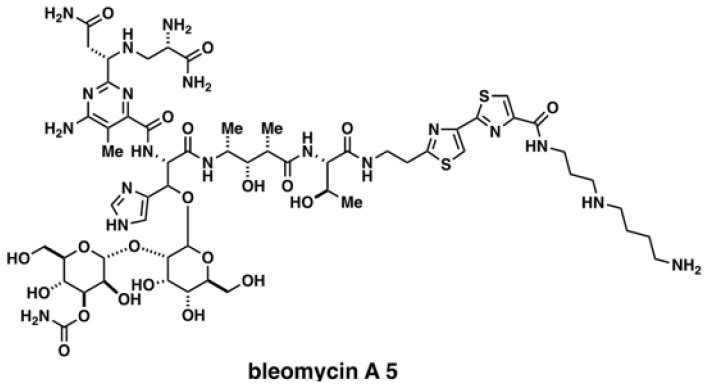
Berberine chloride	DNA intercalator	2086-83-1	Benzo[*g*]-1,3-benzodioxolo[5,6-*a*]quinolizinium, 5,6-dihydro-9,10-dimethoxy- (9CI, ACI)	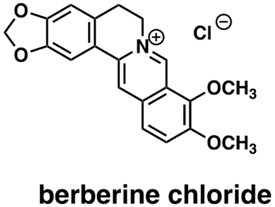
Fusidic acid	Binding to prokaryotic and eukaryotic peptide elongation factors	6990-06-3	29-Nordammara-17(20),24-dien-21-oic acid, 16-(acetyloxy)-3,11-dihydroxy-, (3α,4α,8α,9β,11α,13α,14β,16β,17*Z*)- (9CI, ACI)	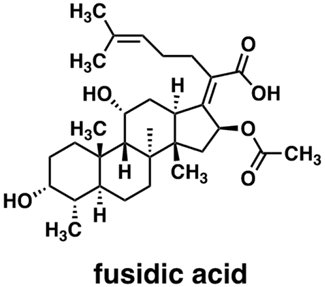
G-418 diastereomer	Possible inhibitor of protein synthesis by targeting 70S and 80S ribosomes	1192351-60-2	(1R,2R,4R,6S)-4,6-Diamino-3-({(5R)-2-amino-2-deoxy-5-[(1S)-1-hydroxyethyl]-α-D-xylopyranosyl}oxy)-2-hydroxycyclohexyl 3-deoxy-4-C-methyl-3-(methylamino)-D-arabinopyranoside	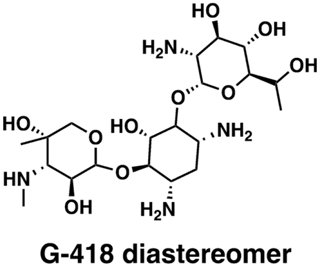
Monensin	Inhibitor of the K^+^ transporter	17090-79-8	(2S,3R,4S)-4-[(2S,5R,7S,8R,9S)-2-{(2S,2'R,3'S,5R,5'R)-2-Ethyl-5'-[(2S,3S,5R,6R)-6-hydroxy-6-(hydroxymethyl)-3,5-dimethyloxan-2-yl]-3'-methyl[2,2'-bioxolan]-5-yl}-9-hydroxy-2,8-dimethyl-1,6-dioxaspiro[4.5]decan-7-yl]-3-methoxy-2-methylpentanoic acid	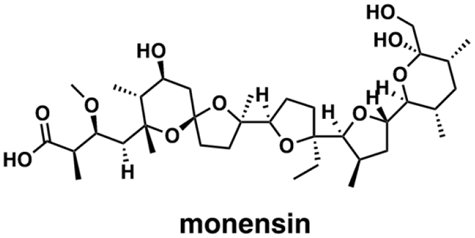
Lovastatin analog	Possible inhibitor of HMG-CoA reductase	2650832-70-3	(1S,3R,7S,8S,8aR)-8-{2-[(2R,4R)-4-hydroxy-6-oxooxan-2-yl]ethyl}-3,7,8a-trimethyl-1,2,3,7,8,8a-hexahydronaphthalen-1-yl (2S)-2-methylbutanoate	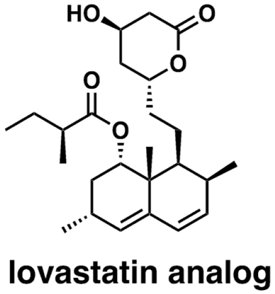

The eight molecules identified and their related molecules were shown in Tables 1 and 2. Four of the molecules (camptothecin, 9-hydroxyl camptothecin, bleomycin A5, and berberine chloride) are known as tumor suppressors in human cells, and inducers of nucleolar stress. Two of the molecules (fusidic acid and G-418 diastereomer) are known as inhibitors in protein synthesis. Monensin is a molecule that has been reported to cause oxidative stress in animal cells. Lovastatin analogs are therapeutic agents for dyslipidemia that act by inhibiting HMG-CoA reductase (HMGR), the rate-limiting enzyme in cholesterol biosynthesis. In addition, these molecules also reduce ATP production, a crucial energy source required for cell survival. Statins are also known to be inhibitors of various tumors.

**Table 2.** Molecules identified in the chemical library, and their related molecules, that induced filamentous leaves in *as2-1* and *as1-1* mutants

Molecular characteristics	Substance	Comments	References
Inhibitors of DNA topo-isomerases	Camptothecin*	A TOPI inhibitor, forming a topoisomerase I-DNA covalent complex	Christensen (2002); Rubbi and Milner (2003); Basili and Moro (2009); Ohwada (2009); Burger (2010); Boulon (2010); Hutten (2011); Grierson (2013); González-Arzola (2024)
	9-Hydroxy-camptothecin*		
	10-Hydroxy-camptothecin		
	Etoposide	A TOPII inhibitor, forming a topoisomerase II-DNA covalent complex	
DNA damge agents	Bleomycin A5*	Leads to double-strand break	Hecht (2000); Palwai and Eriksson (2011)
	Bleomycin A2+B2		
	Mitomycin C	An inhibitor of DNA synthesis by the introduction of cross-links into DNA at purine bases	Chan (1988)
	Hydroxyurea	A DNA replication inhibitor	Luong (2018)
	Berberine chloride*	A DNA intercalator	Nakagawa (2012); Avila-Carrasco (2019)
	Coptisine		
	N-methylmesoporphyrin IX (NMM)		
	Tricostatin A	Epigenetic regulation and an inhibitor of HDAC activity	Ueno (2007)
	5-Aza-2'-deoxycytidine	Epigenetic regulation and an inhibitor of DNA methylation	Iwasaki (2013)
Molecules targeting ribosomes	Fusidic acid*	Binding to prokaryotic and eukaryotic peptide elongation factors	Kim (2023)
	G-418	An inhibitor of protein synthesis by targeting 70S and 80S ribosomes	Bidou (2017); Heldin (2023)
	G-418 diastereomer*	A possible inhibitor of protein synthesis by targeting 70S and 80S ribosomes	None available
Inhibitor of the K^+^ transporter	Monensin*	An inhibitor of the K^+^ transporter and an inhibitor of androgen signaling leading to apoptosis in cancer cells	Ketola (2010); Kocaturk (2019)
Inhibitor of HMG-CoA reductase	Lovastatin analog*	A possible inhibitor of HMG-CoA reductase	None available
	Lovastatin	Molecules that inhibit HMG-CoA reductase, which is a key regulator of metabolism, and are used as drugs to reduce blood cholesterol and fat	Nayan (2017); Beckwitt (2018); Lu (2019)
	Mevastatin		

*Molecules selected by screening from the chemical library.

Among them, in this study, we focused on CPT and 9-hydroxyl-CPT (9H-CPT), which inhibit specifically the action of type IB DNA topoisomerase (Fig. 1B). However, CPT was too strong to control effects on leaf development. Since 9H-CPT was not commercially available, we used 10-hydroxyl-CPT (10H-CPT), which is less toxic than CPT (Fig. 1B; López-Meyer and Nessler, 1997; Lorence et al., 2004), and sought appropriate concentrations of 10H-CPT that affected leaf morphogenesis but not plant survival (Fig. 1C,D).

When *as2-1* and *as1-1* plants were grown on media supplemented with 10H-CPT (0.1-1.0 μM), most of the plants formed leaves with abnormal shapes, which were roughly classified into two groups (Fig. 1C,D): filamentous leaves (Fig. 1D, red arrowheads) and small unexpanded leaves like clumps (Fig. 1D, green arrowheads) between two browned cotyledons (Fig. 1D, blue arrowheads). Even at low concentrations of 10H-CPT (0.2 μM), one half of these mutants generated filamentous or small unexpanded leaves, although almost all (>95%) of the Col-0 plants generated normally expanded leaves under the same growth conditions (Fig. 1C,D). We decided to monitor leaf morphology of plants that had been grown in the presence of 0.15 and/or 0.2 μM 10H-CPT. We should also point that more than half of Col-0 plants produced filamentous leaves at 0.5 μM 10H-CPT (Fig. 1C,D).

We examined levels of transcripts of the abaxial domain-determining gene *ETT/ARF3*, which is a direct downstream target of the AS2–AS1 complex (Iwasaki et al., 2013; Husbands et al., 2015), in shoot apices of 0.15 μM 10H-CPT-treated Col-0, *as2-1* and *as1-1* plants. Real-time quantitative RT-PCR analysis revealed that levels of *ETT/ARF3* transcripts were higher in *as2-1* and *as1-1* than in the Col-0 plants when treated with 10H-CPT (Fig. 1E). This result implies that the higher level of accumulation of transcripts of the abaxial factor *ETT/ARF3* by the treatment with 10H-CPT might be, at least in part, a reason for the generation of filamentous leaves.

To examine the adaxial–abaxial patterning in leaves of 10H-CPT-treated Col-0, *as2-1* and *as1-1* plants, we used the *FILp:GFP* reporter gene in which the coding sequence for green fluorescent protein (GFP) was fused to the *FILAMENTOUS FLOWER* (*FIL*) promoter active in abaxial cells of wild-type leaves. We made transgenic plants (Col-0, *as2-1* and *as1-1*) expressing the reporter gene and treated these transgenic plants with 10H-CPT to induce filamentous leaves. As shown in Fig. 1F, strong signals of the GFP fluorescence were detected in cells of the surface of the filamentous leaves induced by the treatment of *as2-1* and *as1-1* plants with 10H-CPT (upper and lower right panels), whereas signals were detected in cells located in the abaxial epidermis of the leaves of *as2-1* and *as1-1* plants without 10H-CPT (upper and lower left panels). Thus, the 10H-CPT treatment of *as2* and *as1* plants failed to develop the adaxial domain during leaf morphogenesis.

The GUS activity was observed in filamentous leaves of 10H-CPT-treated *as2* plants expressing *ETTp:GUS* (Fig. 1G). We analyzed GUS activity in filamentous leaves of 10H-CPT-treated *BREVIPEDICELLUS* (*BP*)*p:GUS/as2* plants, but GUS activity was not observed (Fig. S1A). The *bp-1 knat2-3 knat6-2* mutation did not suppress the filamentous leaf phenotype in 10H-CPT-treated *as2-1* mutants (Fig. S1B). These results suggested that the filamentous leaves produced in *as2-1* plants treated with 10H-CPT are abaxialized.

### *AS2* and *TOP1α* repress *ETT/ARF3* expression

An endogenous target molecule of CPT is TOPI, which loosens the super-coils and loop structures in double-stranded DNAs by mediating single-strand breakages and reunions. In the *A. thaliana* genome, TOPI proteins are encoded by two genes, *TOP1α* and *TOP1β*, which are tandemly arrayed (Fig. S2A) (Takahashi et al., 2002). We investigated genetic interactions among *AS2*, *AS1*, *TOP1α* and *TOP1β*. The *as2-1 top1α-1* and *as1-1 top1α-1* double mutants exhibited phenotypes of the severely filamentous leaves (Fig. 2A, 2nd row; Fig. S2B). Similar phenotypes were also observed in *as2-1 mgo1-7* and *as1-1 mgo1-7* double mutants (*mgo1-7* is another allele of *top1α*) (Fig. S2A-C; Graf et al., 2010). However, mutants of *as2-1 top1β-1* and *as1-1 top1β-1* (or *top1β-2*) did not generate filamentous leaves (Fig. S2D). Thus, filamentous leaves are a synthetic phenotype specifically generated by the double mutants *as2-1 top1α-1* and *as1-1 top1α-1*, but not by *top1β*.

**Fig. 2.**
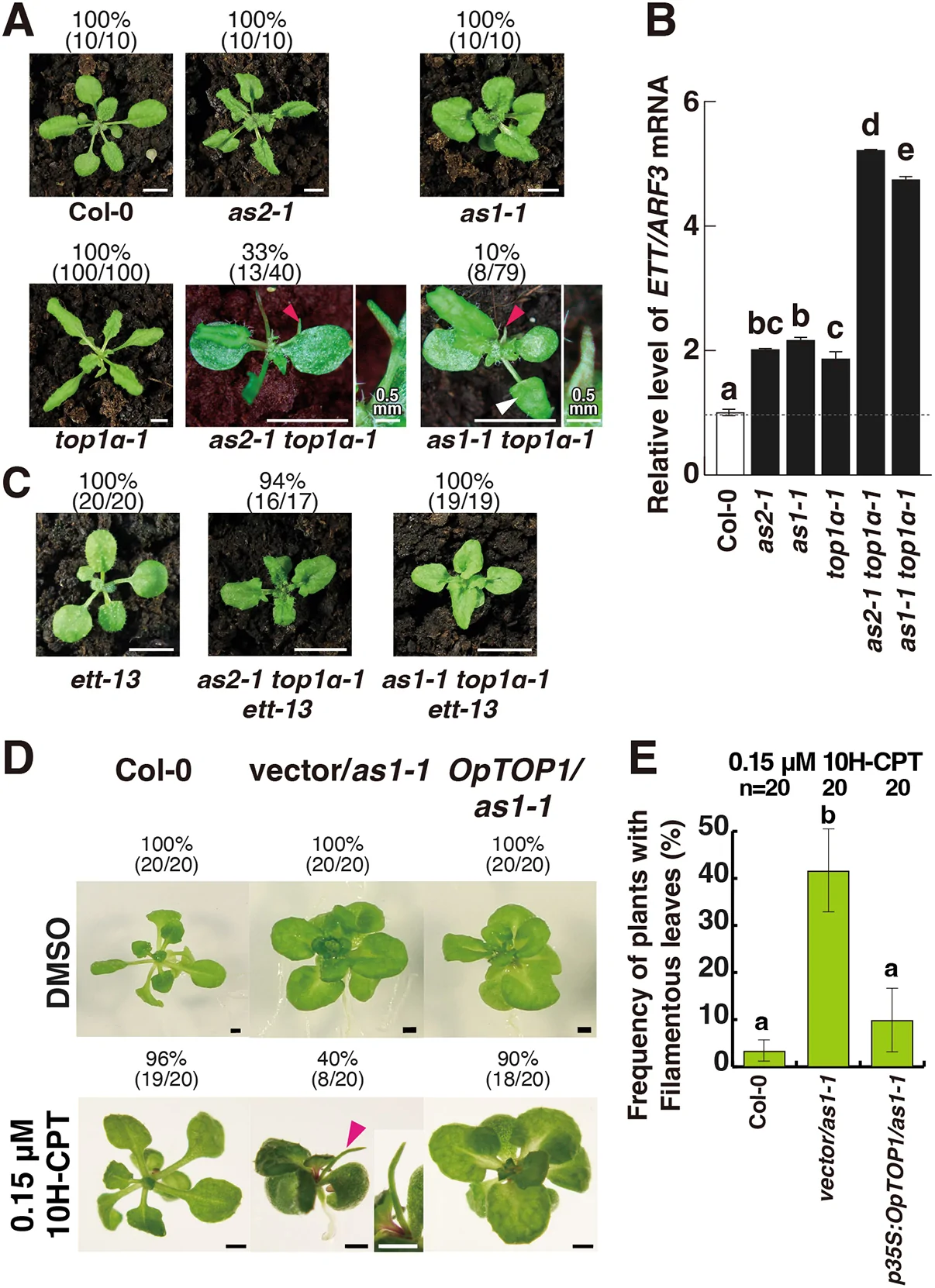
***TOP1α* and *AS2–AS1* cooperatively function in the development of expanded leaves with the proper adaxial–abaxial polarity.** (A) The mutation of *TOP1α* caused impairment of leaf adaxial–abaxial polarity in *as2-1* and *as1-1* backgrounds. Leaf phenotype of the plants at 21 or 22 days after sowing (DAS). Scale bars: 5 mm (unless otherwise indicated). Red arrowheads indicate filamentous leaves. Values above each image indicate the frequency (%) of each leaf phenotype. Numbers in parentheses represent the number of seedlings with the indicated phenotype/total number of seedlings examined. (B) Changes (mean±s.d.) in transcription levels of the *ETT*/*ARF3* gene of shoot apices in plants grown for 14 days. Each value was normalized by reference to the level of *ACT2* transcripts. The values are shown relative to the value for Col-0 plants (dashed line) (*P*<0.05, Tukey's test after significant ANOVA). (C) Suppression of leaf phenotypes of *as2-1 top1α-1* by *ett-13*. Leaf phenotype of the mutant plants at 22 DAS. Scale bars: 10 mm. Values above each image indicate the frequency (%) of each leaf phenotype. Numbers in parentheses represent the number of seedlings with the indicated phenotype/total number of seedlings examined. (D) The *OpTOP1* gene derived from the CPT-producing herb *Ophiorrhiza pumila* attenuates the action of CPT to induce the leaf filaments in *Arabidopsis*. Comparison of leaf morphology among Col-0, *as1-1* and *as1-1*-expressing *OpTOP1* plants growing in the absence or the presence of 10H-CPT for 21 days after sowing*.* Values above each image indicate the frequency (%) of each leaf phenotype. Numbers in parentheses represent the number of seedlings with the indicated phenotype/total number of seedlings examined. (E) Quantitative analyses of frequencies of filamentous leaves in plants of Col-0, *as1-1* transformed with empty vector pGWB2 and *as1-1*-expressing *OpTOP1*. Data are mean±s.d. of three replicates (*n*=20 plants for each). Letters above bars indicate significance (*P*<0.05, Tukey's test after significant ANOVA).

Since the *ETT/ARF3* gene is a direct target of the AS2–AS1 complex (Iwasaki et al., 2013; Husbands et al., 2015), we examined levels of transcripts in *top1α-1*, *as2-1 top1α-1* and *as1-1 top1α-1*. As shown in Fig. 2B, RT-PCR analysis revealed that levels of *ETT/ARF3* transcript were higher in the shoot apices of these double mutants than in Col-0 plants and each single mutant (*top1α-1*, *as2-1* and *as1-1*). The above results suggest that *TOP1α* and *AS2–AS1* synergistically repress transcript levels of the *ETT/ARF3* gene, the abaxial-determinant, which leads to the development of the adaxial domain of leaves. We examined whether increased levels of *ETT/ARF3* transcripts might be responsible for the formation of filamentous leaves in *as2-1 top1α-1* and *as1-1 top1α-1* (Fig. 2A) by introducing the *ett-13* mutation into each of the double mutants. As shown in Fig. 2C, the *ett-13* mutation suppressed the filamentous leaf phenotype in these double mutants. These results suggest that the increase of levels of the *ETT/ARF3* expression in *as2-1 top1α-1* and *as1-1 top1α-1* plants causes the formation of filamentous leaves. In Col-0, repression of *ETT/ARF3* by a cooperative action of *TOP1α* and *AS2-AS1* is crucial in leaf development with adaxial–abaxial polarity. These results, together with those of the previous section with 10H-CPT, imply that a synergistic action of *AS2* (and *AS1*) and *TOP1α* might play an essential role in the development of flat and expanded leaves in *Arabidopsis*.

### *OpTOP1* from camptothecin-producing herb confers camptothecin resistance in *Arabidopsis*

CPT was originally discovered in a medical plant, *Camptotheca acuminata*, and is known to be produced by many species of *Ophiorrhiza* including *Ophiorrhiza pumila* (Sirikantaramas et al., 2008). TOPI proteins of *Ophiorrhiza* plants (*Op*TOP1) have mutated amino acid residues in the region that are responsible for the catalytic function or CPT binding when compared to those of *A. thaliana* and *Homo sapiens*. Yeast cells expressing the *OpTOP1* cDNA from *Ophiorrhiza pumila* confer higher resistance to CPT than those expressing *TOP1* cDNAs of *A. thaliana* and *H. sapiens.* The 35S promoter-fused *OpTOP1* cDNA was constructed and introduced into the *as1-1* mutant, and the resulting transgenic plants were tested for the effects of 0.15 μM 10H-CPT on filamentous leaf formation. Fig. 2D showed that 90% of the *as1-1* mutants expressing *OpTOP1* formed normally expanding leaves in the presence of 0.15 μM 10H-CPT, although ∼40% of *as1-1* transformed with empty vector pGWB2 formed filamentous leaves at the same concentration of 10H-CPT (Fig. 2D,E). These results suggest that *OpTOP1* in the *as1-1* mutant is functionally replaced with activity of endogenous TOP1 of *Arabidopsis* that was sensitive to 0.15 μM 10H-CPT*.* Thus, type IB topoisomerase plays a crucial role in the formation of a flat and symmetrically expanding leaves.

### *KRP5/ICK3* is regulated by *AS2* and *TOP1α*

In the present experiment, we analyzed patterns of gene expression data from DNA microarrays using mRNAs from shoot apices of the 10H-CPT-treated *as2-1* and Col-0 plants, and extracted genes regulated downstream of AS2, which were classified into 30 clusters by using the knowledge-based fuzzy adaptive resonance theory (KB-FuzzyART) (Takahashi et al., 2003, 2008, 2013) (Fig. S3A; Table S1). Expression levels of genes that were classified to clusters 10, 12, 16 and 25 (Fig. 3A, Fig. S3A-C) were increased in both *as2-1* and Col-0 treated with 10H-CPT. Genes involved in ‘cell fate’ (such as *BP* and *ETT*/*ARF3*) were significantly enriched in the clusters 10, 12, 16 and 25 (Fig. S3A; Fig. S3B; Tables S2-S4). Therefore, we focused on those 556 genes in those clusters (Fig. 3A).

**Fig. 3.**
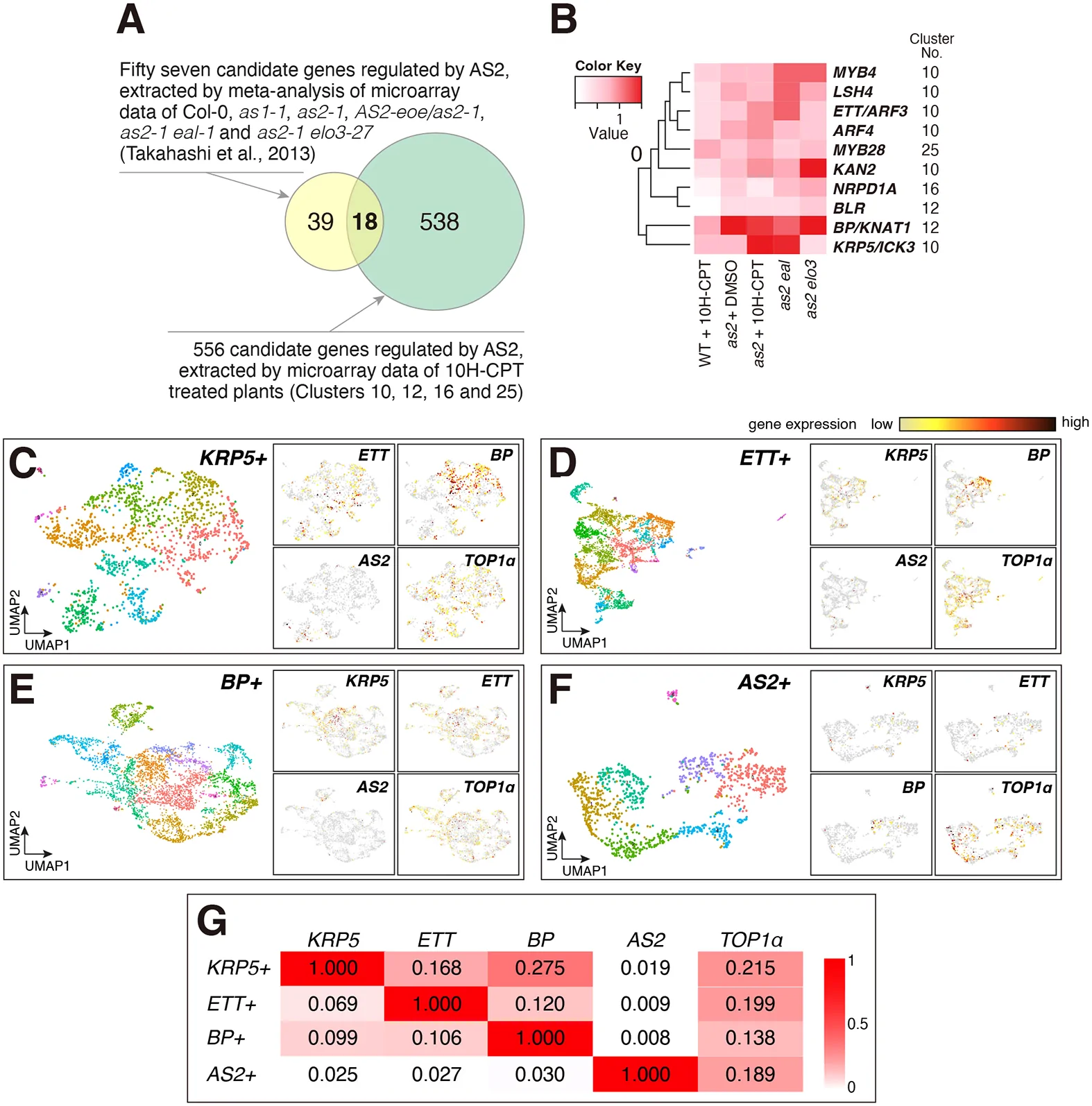
**The *KRP5*/*ICK3* gene is a downstream gene regulated by *AS2* and *TOP1α.*** (A) Venn diagram that shows the relationship between 57 candidates regulated by *AS2*, *AS1*, *ELO3* and *EAL* (Takahashi et al., 2013), and 556 genes in clusters 10, 12, 16 and 25 extracted in this experiment (Fig. S3). Eighteen genes overlapped. (B) Heatmap representing transcript levels of 10 genes that are candidates for being downstream of AS2–AS1. (C-F) UMAP visualization of cell populations expressing *KRP5* (KRP5+, *n*=1583), *ETT* (ETT+, *n*=3879), *BP* (BP+, *n*=4393) and *AS2* (AS2+, *n*=1221). Individual dots represent single cells. For each panel, a UMAP plot colored by cell cluster is shown on the left, while four UMAP plots displaying specific gene expression patterns are presented on the right. The color scale for expression levels is indicated at the top. (G) Heatmap showing co-expression ratios. The values represent the proportion of cells expressing the genes indicated at the top within the *KRP5+*, *ETT+*, *BP+* and *AS2+* cell populations. The color scale for values is indicated on the right.

As we have previously reported, mutations of *BOBBER1* (*BOB1*)/*ENHANCER of AS2 and AS1* (*EAL*) and *ELONGATA3* (*ELO3*)/*ENHANCER of ASYMMETRIC LEAVES TWO1* (*EAST1*) act as modifiers of *as2* and *as1*, enhancing adaxial defects and leading to the formation of filamentous leaves with an abaxialized surfaces. In addition, the expression levels of many genes, including *ETT/ARF3*, are elevated in *as2 bob1* and *as2 elo3* double mutants (Kojima et al., 2011; Ishibashi et al., 2012). *BOB1*/*EAL* is a gene that encodes an *Arabidopsis* ortholog of eukaryotic NudC domain proteins (Jurkuta et al., 2009; Perez et al., 2009; Ishibashi et al., 2012). *ELO3*/*EAST1* is a gene that encodes the histone acetyltransferase, a component of the Elongator complex (Wittschieben et al., 1999; Pandey et al., 2002; Kojima et al., 2011).

We have previously carried out clustering analysis using KB-FuzzyART (Takahashi et al., 2013; Iwasaki et al., 2013), using microarray data of *as2 bob1* and *as2 elo3* double mutants (Kojima et al., 2011; Ishibashi et al., 2012). We extracted 57 overlapping genes co-regulated by both *BOB1*/*EAL* and *ELO3*/*EAST1* (Fig. 3A; Kojima et al., 2011; Ishibashi et al., 2012; Takahashi et al., 2013). We further identified downstream factors by merging between 57 candidates regulated by *AS2*, *AS1*, *ELO3* and *EAL* (Takahashi et al., 2013) and 556 genes in clusters 10, 12, 16 and 25 extracted in this experiment (Fig. S3). Results showed that 18 genes overlapped (Fig. 3A).

The group of 18 selected genes included 10 genes encoding proteins with well-characterized domains, such as *ETT*/*ARF3* and *BP*, among which *KRP5/ICK3*, a Cip/Kip family CDK inhibitor gene, was included as a gene in the cluster of ‘division-related genes’ (Fig. S3B,D; Table S1). The level of *KRP5/ICK3* transcripts was increased in 10H-CPT-treated *as2-1* plants (Fig. S3E). The transcription levels of the other six *KRP/ICK* genes did not increase in the *as2-1* mutant treated with 10H-CPT, indicating that *KRP5*/*ICK3* is the only downstream gene within this family (Figs S3E,F and S7B).

Using a published scRNA-seq dataset of *Arabidopsis* vegetative shoot apices and leaves (BioProject PRJCA003094; Zhang et al., 2021), we re-clustered and visualized cell subsets expressing *KRP5*/*ICK3*, *ETT/ARF3*, *BP* and *AS2*, designated as *KRP5+*, *ETT+*, *BP+* and *AS2+*, respectively, via UMAP (Fig. 3C-F, Fig. S4). Ratios of co-expressing cells revealed that *KRP5/ICK3* was co-expressed with *ETT/ARF3* (16.8%) and *BP* (27.5%), but rarely with *AS2* (1.9%), suggesting that *KRP5/ICK3* may be expressed in very early leaf primordia (Fig. 3G). Conversely, *AS2* was minimally co-expressed with *KRP5/ICK3* (2.5%), *ETT/ARF3* (2.7%) and *BP* (3.0%) (Fig. 3G). These data reinforce earlier microarray evidence indicating that *AS2* acts as a repressor of these genes during leaf development.

We next examined the expression pattern of KRP5/ICK3 using a *pKRP5:KRP5-GUS* transgenic line. Weak GUS activity was observed in the internal tissues of young leaf primordia at the shoot apex (Fig. S5). On the other hand, *AS2* is expressed in the adaxial region of leaf primordia, including the epidermal cells (Iwakawa et al., 2007). These observations are consistent with scRNA-seq results indicating that *KRP5*/*ICK3* is expressed in cell populations distinct from those expressing *AS2*.

### *AS2* and *TOP1α* repress *KRP5*/*ICK3* to promote normal leaf expansion

Treatment of the *as2-1* plant with 10H-CPT gave rise to the production of filamentous leaves in 35% of the plants. The efficiency of occurrence of such filamentous leaves in the *as2-1 krp5-1* mutant was reduced to only 5% (Fig. 4A,B). Treatment of *as2-1 krp5-4* with 10H-CPT similarly reduced the efficiency of formation of filamentous leaves (Fig. S6). While treatment of *as2-1 krp2-5* with 10H-CPT did not reduce the efficiency of formation of filamentous leaves (Fig. S7). Thus, negative regulation of *KRP5/ICK3* should be necessary for the formation of normally expanded leaves.

**Fig. 4.**
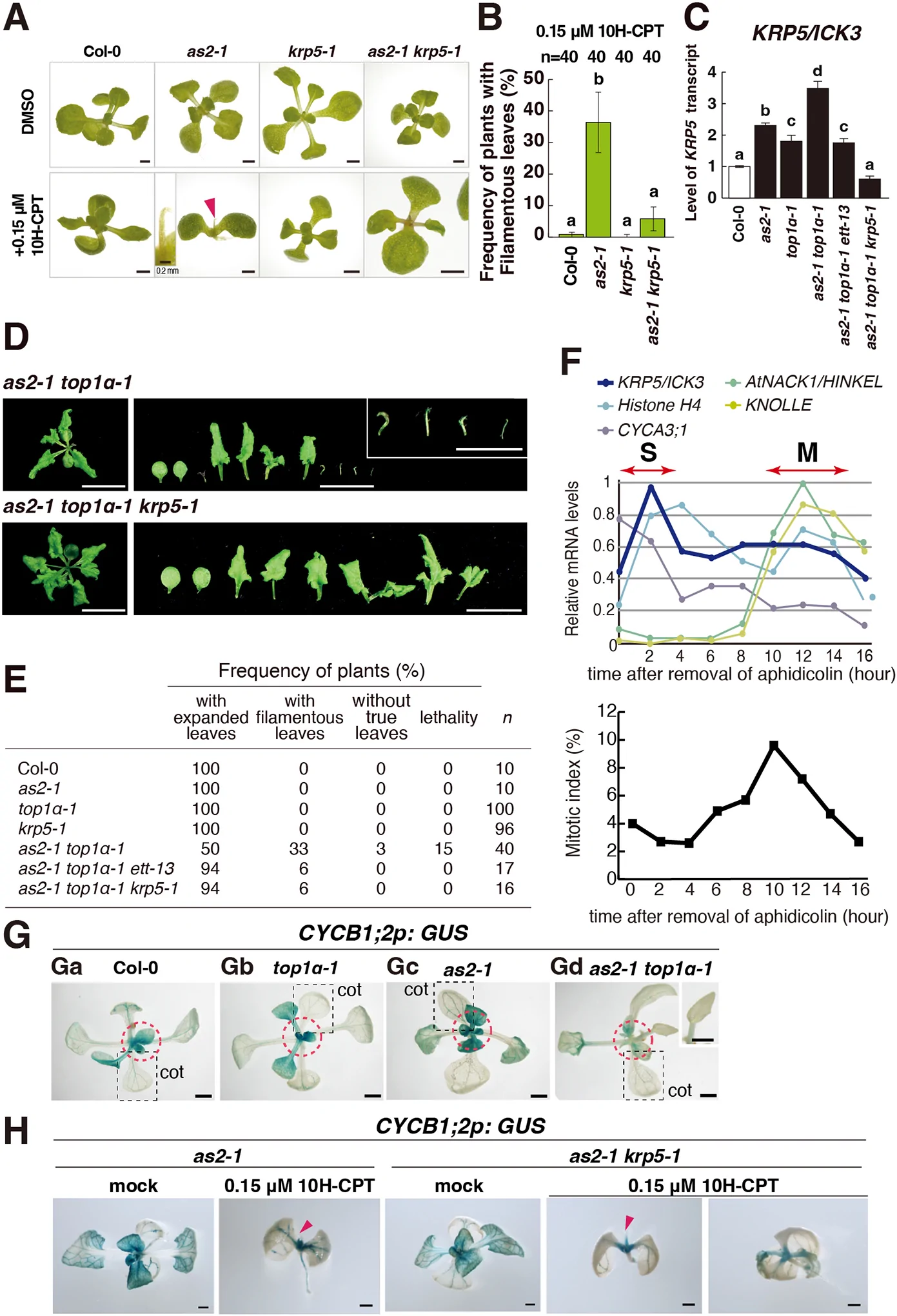
***AS2* and *TOP1α* repress the *KRP5/ICK3* gene to promote leaf expansion.** (A) The *krp5-1* mutation suppressed filamentous leaf phenotype in *as2-1* treated with 0.15 μM 10H-CPT from sowing for 14 days. Scale bars: 1 mm. A red arrowhead indicates a filamentous leaf. (B) Frequencies of plants with filamentous leaves at the 1st and 2nd leaf positions when plants were treated with 0.15 μM 10H-CPT Data are mean±s.d. of three replicates (*n*=40 plants for each) (*P*<0.05, Tukey's test after significant ANOVA). (C) Changes in levels of transcript of *KRP5* in shoot apices of plants grown on soil for 14 days*.* Each value was normalized by reference to the level of *ACTIN2* transcripts (*P*<0.05, Tukey's test after significant ANOVA). (D) Gross morphology of *as2-1 top1α-1* and *as2-1 top1α-1 krp5-1.* Scale bars: 10 mm (5 mm in inset of *as2-1 top1α-1*). Plants photographed at 22 days after sowing. (E) Frequencies of phenotypes of leaves and plant lethality at 22 DAS. (F) Transcript levels of the *KRP5/ICK3* gene (upper panel) and mitotic index (lower panel) in aphidicolin-synchronized *Arabidopsis* MM2d cells. (G) Representative GUS expression patterns of 13-day-old Col-0 (wild type, Ga), *top1α-1* (Gb), *as2-1* (Gc) and *as2-1 top1α-1* (Gd) mutant plants that harbored the *CYCB1;2p:GUS*. Shoot apices are marked by dashed red circles and cotyledons are marked by dashed black squares. Scale bars: 1 mm. (H) The GUS expression pattern of *CYCB1;2p:GUS*/*as2-1* and *CYCB1;2p:GUS*/*as2-1 krp5-1* plants treated with 0 μM or 0.15 μM 10H-CPT for 13 days after sowing. Red arrowheads indicate filamentous leaves.

We next quantified *KRP5/ICK3* transcript levels in the *as2-1* and *top1α-1* single mutants and in the *as2-1 top1α-1* double mutant. As shown in Fig. 4C, transcript levels were higher in the double mutant than in either of the single mutants. Introduction of the *ett-13* mutation into the *as2-1 top1α-1* double mutant reduced *KRP5*/*ICK3* transcript levels, suggesting that repression of *KRP5*/*ICK3* by *AS2* and *TOP1α* is mediated through *ETT*/*ARF3*.

We examined whether filamentous leaf phenotypes of the *as2-1 top1α-1* double mutant might be suppressed by introduction of the *krp5-1* mutation. As shown in Fig. 4D,E, the introduction of the *krp5-1* mutation into the double mutant decreased the proportion of plants showing the filamentous leaves (from 33% to 6%).

We measured relative levels of *KRP5/ICK3* transcripts during cell cycle progression of synchronized cultured cell line MM2d of *A. thaliana* (Fig. 4F). The transcript levels of *KRP5/ICK3* and *HISTONE H4* (a S phase marker) peaked at 2-4 h after the removal of aphidicolin (corresponding to S phase), being consistent with previous microarray results (Menges and Murray, 2002). The broad pattern of transcripts of *KRP5/ICK3* was also found during the progression of M phase, overlapping with peaks of the transcripts of M-phase-specific genes such as kinesin *AtNACK1/HINKEL* (Nishihama et al., 2002; Strompen et al., 2002) and *KNOLLE* (Heese et al., 2001). These results suggest that *KRP5*/*ICK3* expression is cell cycle dependent and associated with cell cycle progression.

Using transgenic plants expressing the *CYCB1;2p:GUS* gene (Ishibashi et al., 2012), we examined the cell division activity of local regions in shoot apices that cover developing leaves of wild type (Col-0), *top1α-1*, *as2-1* and *as2-1 top1α* plants (marked by dashed red circles). As shown in Fig. 4G, clear GUS activities were detected as blue staining in these regions. Only a limited level of GUS activity was detected in shoot apices of the double mutant (Fig. 4G, Fig. S8A,B). When *CYCB1;2p:GUS*/*as2-1* plants were treated with 10H-CPT, GUS activity was detected at low levels in the rod-shaped leaves (Fig. 4H). Several *CYCB1;2p:GUS*/*as2-1 krp5-1* plants formed true leaves, in which GUS activity was detected in the petioles and vascular bundles (Fig. 4H, Fig. S8C).

These results suggest that *AS2* and *TOP1α* promote cell proliferation in developing leaves by repressing *KRP5*/*ICK3*, which is required for the formation of flat expanded leaves. However, Fig. S8C shows no difference in signal between CPT-treated *CYCB1;2p:GUS*/*as2-1* and CPT-treated *CYCB1;2p:G*US/*as2-1 krp5-1*, suggesting that *KRP5*/*ICK3* alone does not regulate cell proliferation.

### Inhibitors of topoisomerase II and DNA synthesis induce filamentous leaf formation in the *as2* mutant

As shown in Fig. 5A,C and Fig. S9A,B, we further examined the effects of the following reagents on leaf development in the *as2-1* and *as1-1* backgrounds: etoposide, a specific inhibitor of type II DNA topoisomerase (TOPII); mitomycin C, an inhibitor of DNA synthesis that introduces cross-links into DNA at purine bases; and bleomycin A5, which induces double-strand breaks (Kampranis et al., 1999; Yogo et al., 2012; Hutten et al., 2011; Burger et al., 2010; Boulon et al., 2010; González-Arzola, 2024). Etoposide, mitomycin C and bleomycin A5 have also been shown to induce nucleolar stress in animal cells (Hutten et al., 2011; González-Arzola, 2024). The *as2-1* and *as1-1* mutant plants that were grown in the presence of these molecules formed filamentous leaves, although Col-0 plants scarcely formed, as observed upon the treatment with 10H-CPT (Fig. 5A,C, Fig. S9A,B).

**Fig. 5.**
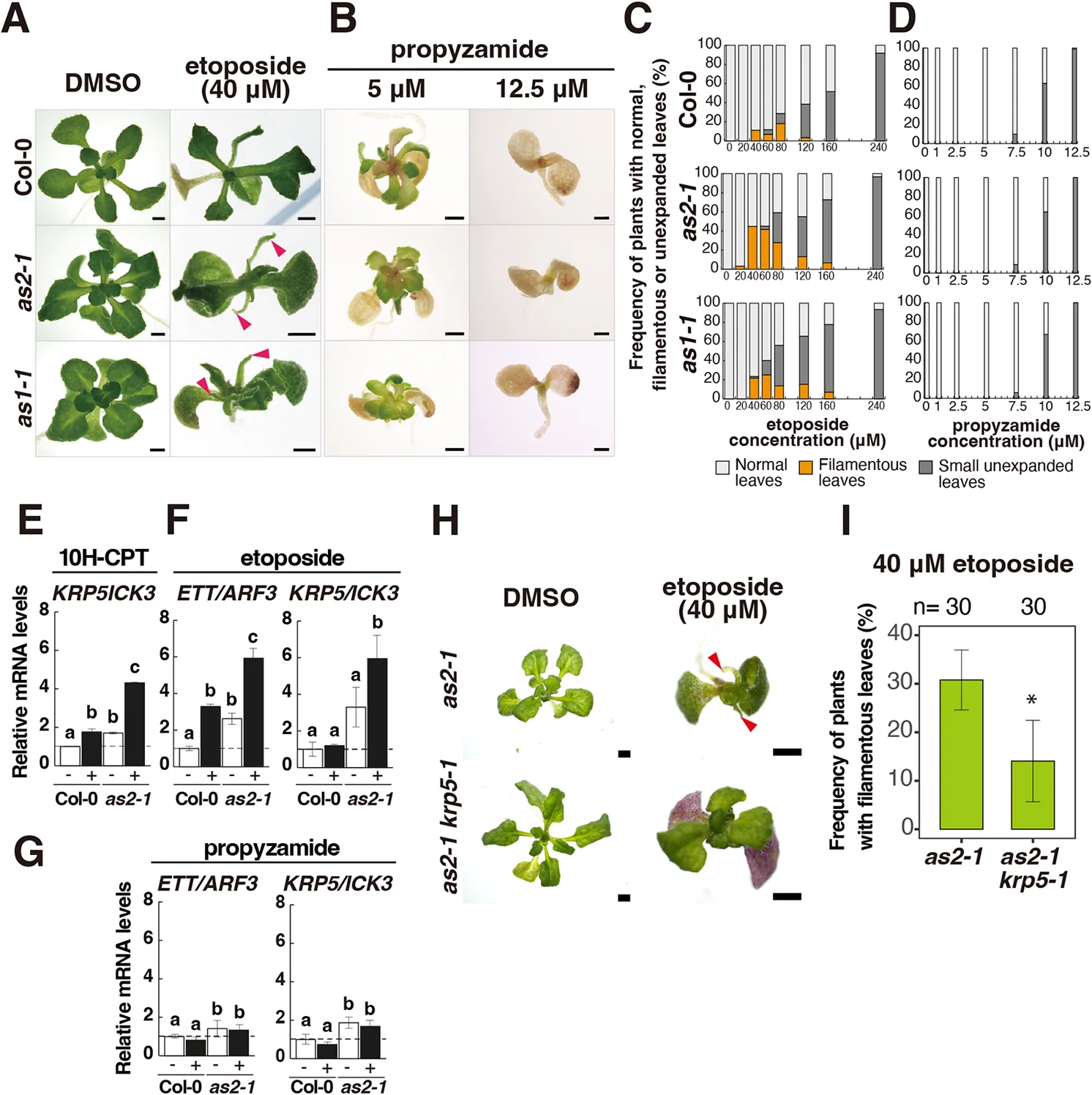
**Inhibitors of topoisomerase II induce formation of filamentous leaves, but inhibitors of microtubule turnover do not.** (A) Phenotypes of Col-0, *as2-1* and *as1-1* plants in the presence of DMSO and etoposide for 21 days after sowing. Red arrowheads indicate filamentous leaves. Scale bars: 1 mm. (B) Representative phenotypes of plants treated with 5 and 12.5 μM propyzamide for 21 days after sowing. Scale bars: 1 mm. (C,D) Dose-dependent effects of etoposide (C) and propyzamide (D) on leaf abnormalities and leaf formation. Each value is calculated from the means of three biological replicates (*n*=20 plants for etoposide and *n*=24 plants for propyzamide). (E) Changes (mean±s.d.) in transcription levels of *KRP5/ICK3* in shoot apices of Col-0 and *as2-1* treated without and with 10H-CPT for 14 days after sowing. Dashed line indicates the value for Col-0. (F,G) Changes in transcription levels of *ETT*/*ARF3* and *KRP5/ICK3* in shoot apices of plants treated without and with 40 μM etoposide (F), and with 5 μM propyzamide (G) for 14 days after sowing (*P*<0.05, Tukey's HSD test after significant ANOVA). (H) Phenotypes of *as2-1* and *as2-1 krp5-1* plants in the presence of DMSO or 40 μM etoposide for 22 days after sowing. Red arrowheads indicate filamentous leaves. Scale bars: 1 mm. (I) Frequencies of plants with filamentous leaves in *as2-1* or *as2-1 krp5-1* plants treated with 40 μM etoposide. Data are mean±s.d. *P<0.05 (unpaired one-tailed Student's *t*-test).

We subsequently examined effects of reagents such as propyzamide, oryzalin and paclitaxel that inhibit turnover of microtubules, which inhibits the progression of the M phase of the cell cycle at prometaphase (Kakimoto and Shibaoka, 1988; Nishihama et al., 2001, 2002; Sasabe et al., 2011). Upon the treatments with these inhibitors, only dose-dependent growth inhibition was observed, but no filamentous leaves were generated (Fig. 5B,D, Fig. S9A,B).

We next measured levels of *ETT/ARF3* and *KRP5*/*ICK3* transcripts in *as2-1* plants that had been treated with etoposide and propyzamide. The results showed that the levels of *ETT*/*ARF3* and *KRP5*/*ICK3* transcripts significantly increased in shoot apices of 10H-CPT, etoposide-treated *as2-1* plants (Fig. 5E,F). However, levels of transcripts of both genes did not further increase in the propyzamide-treated *as2-1* plant (Fig. 5G).

Etoposide treatment induced filamentous leaves in 30% of *as2-1* plants (Fig. 5H,I). The incidence of filamentous leaves was reduced to 10% in the *as2-1 krp5-1* mutant (Fig. 5H,I). Thus, inhibition of TOPI and TOPII was closely associated with filamentous leaf formation, and increased *ETT/ARF3* and *KRP5/ICK3* transcript levels. After treatment with mitomycin C and bleomycin A5, the incidence of filamentous leaves in the *as2-1 krp5-1* mutant was, on average, lower than in *as2-1*, although the difference was not statistically significant (Fig. S9C-F).

### AS2 attenuates nucleolar stress induced by 10H-CPT treatment and *TOP1α* loss through subnucleolar patterning of AS2 bodies

Fluorescence microscopy using the recombinant AS2-YFP fusion protein has shown that the AS2-YFP signal typically appears as two speckle-like structures, termed AS2 bodies, at the periphery of the nucleolus (Ueno et al., 2007; Luo et al., 2012, 2020; Ando et al., 2023). We previously reported that nucleolar proteins such as NUC1, RH10 and RID2 are involved in the subnucleolar patterning of AS2 bodies (Ando et al., 2023). We examined whether 10H-CPT and etoposide affect the morphology and number of AS2 bodies in the nucleolus. As shown in Fig. 6A,B, the number of AS2 bodies markedly increased and their morphology became more heterogeneous with increasing concentrations of 10H-CPT and etoposide.

**Fig. 6.**
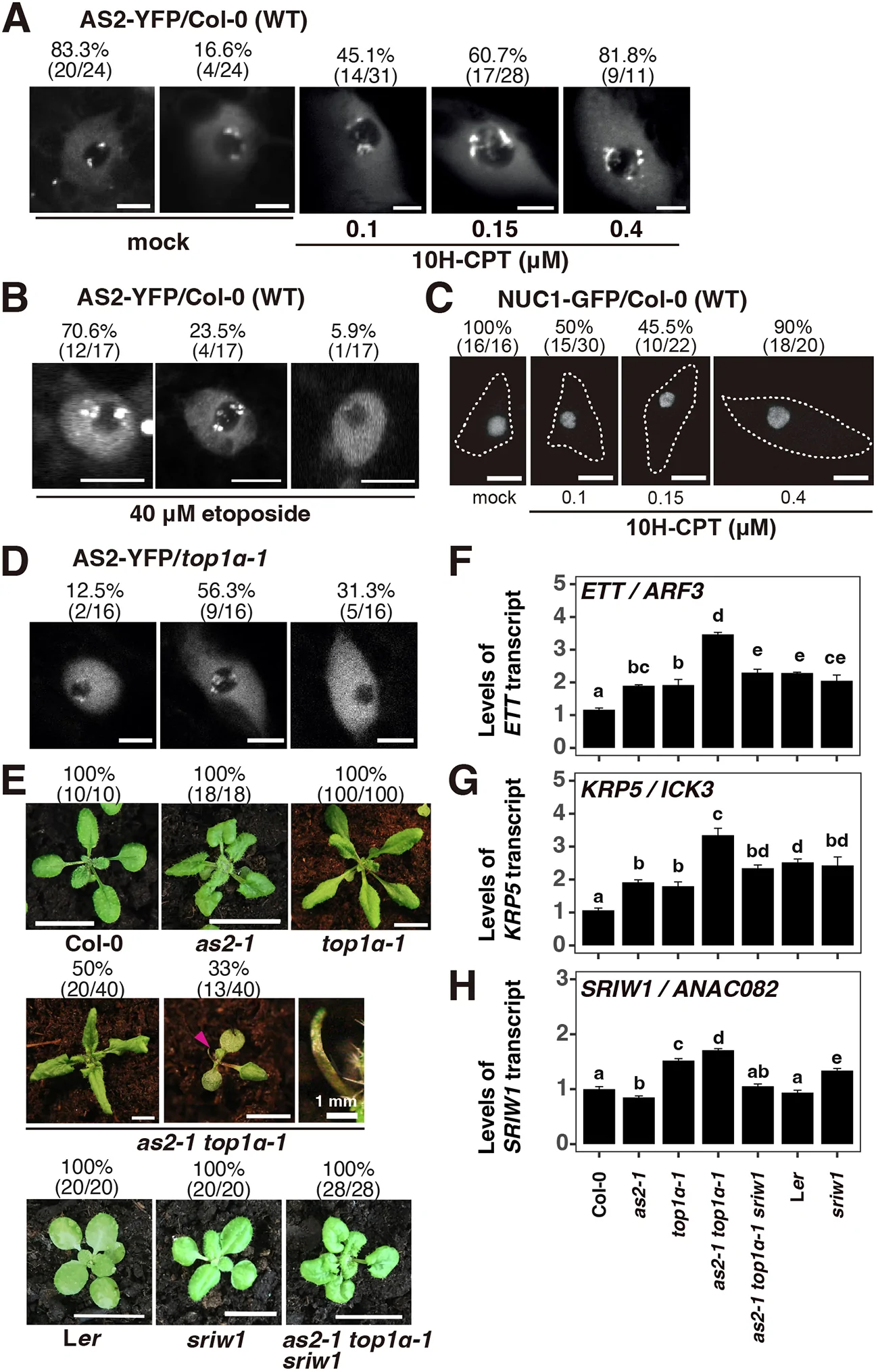
**Treatment with 10H-CPT and the *top1α* mutation affects subnucleolar patterning of AS2 bodies; the *sriw1* mutation suppresses leaf morphology in *as2-1 top1α-1.*** (A) Images showing the signals from AS2-YFP fusion proteins in abaxial cotyledons of Col-0 (wild-type) plants, treated with DMSO (mock), 0.1, 0.15 or 0.4 μM 10H-CPT for 7 days after sowing. (B) Images showing the signals from AS2-YFP fusion proteins in abaxial cotyledons of Col-0 (wild-type) plants, treated with 40 μM etoposide for 7 days after sowing. (C) Images showing signals from NUC1-GFP fusion protein in abaxial cotyledons of Col-0 plants treated with DMSO, 0.1, 0.15 or 0.4 μM 10H-CPT for 7 days after sowing. (D) Images showing the signals from AS2-YFP fusion proteins in abaxial cotyledons of the 7-day-old *top1α*-*1* mutant. GFP-positive nucleoli YFP-positive nuclei in A-D were observed in cells of the adaxial epidermis of cotyledons. Photographs in A, B and D show different types of subnucleolar patterning of AS2 bodies. Numbers above each photo show numbers of cells with AS2 body patterning exemplified by the photo/numbers of cells examined. Scale bars: 5 μm. Photographs in C show different types of NUC1-GFP patterning. Numbers above photos show the numbers of nucleoli with uniform GFP patterning (mock) or the numbers of nucleoli with non-uniform GFP patterning (10H-CPT-treated plants) per numbers of nuclei examined. The dotted lines indicate the nuclear outline. Scale bars: 5 μm. (E) Suppression of leaf phenotypes of *as2-1 top1α*-*1* by *sriw1*, which has mutation in *ANAC082*. Leaf phenotype of the plants grown on soil for 22 days after sowing. Scale bars: 10 mm. Photographs show different types of leaf morphology. Numbers above each photo show the numbers of plants with leaf morphology exemplified by the photo/numbers of plants examined. (F-H) Changes in transcription levels of *ETT*/*ARF3, KRP5/ICK3* and *SRIW1/ANAC082* in shoot apices of plants grown on soil for 14 days. Data are mean±s.d. (*P*<0.05, Tukey's HSD test after significant ANOVA).

In NUC1-GFP transgenic plants, the GFP signal was uniformly distributed throughout the nucleolus (Fig. 6C). However, upon treatment with 10H-CPT, the GFP signal exhibited a non-uniform distribution within the nucleolus (Fig. 6C), suggesting that 10H-CPT induces structural changes in the nucleolus.

We next examined effects of the *top1α-1* mutation on morphology of AS2 bodies. In the *top1α-1* mutant, 56% (9/16) of cells showed an increased number of AS2 bodies, whereas 31% (5/16) lacked obvious AS2 bodies (Fig. 6D). These observations suggest that TOP1α is required for proper formation and localization of the two AS2 bodies in the nucleolus. Similar alterations were observed in the *nuc1*, *rh10* and *rid2* mutants (Luo et al., 2020; Ando et al., 2023).

It has been reported that the *sriw1* mutant, which carries a mutation in *ANAC082*, a gene involved in the nucleolar stress response, suppresses the narrow-leaf phenotype of the *rid2* and *rh10* mutants (Ohbayashi et al., 2017; Ohbayashi and Sugiyama, 2018). The *top1α-1* mutant formed narrow leaves, as observed in *rid2* and *rh10* plants (Figs 2A and 6E). To determine which leaf phenotypes of *as2-1 top1α-1* were suppressed by *sriw1*, we generated the *as2-1 top1α-1 sriw1* triple mutant. The triple mutant did not exhibit a filamentous leaf phenotype and showed a phenotype nearly identical to that of *as2-1* (Fig. 6E).

We next examined *ETT/ARF3*, *KRP5/ICK3* and *SRIW1/ANAC082* transcript levels in these mutant plants. All three transcripts accumulated to higher levels in the *as2-1 top1α-1* mutant, and these increases were suppressed by the *sriw1* mutation (Fig. 6F-H). *SRIW1/ANAC082* transcript levels were increased in shoot apices of *top1α-1*, and further elevated in the *as2-1 top1α-1* mutant (Fig. 6H). These results suggest that nucleolar stress is induced in the *top1α* mutant. AS2 may alleviate leaf formation defects under nucleolar stress caused by the *top1α* mutation.

## DISCUSSION

### AS2 functions in leaf morphogenesis and mitigation of nucleolar stress through AS2 body formation

Our results show that *AS2* and *TOP1α* cooperate to repress the abaxial determinant *ETT/ARF3* and a CDK inhibitor, *KRP5*/*ICK3*, a process required for the formation of flat expanded leaves (Figs 2 and 4). Furthermore, our results suggest that AS2 mitigates nucleolar stress through AS2 body formation (Fig. 7A).

**Fig. 7.**
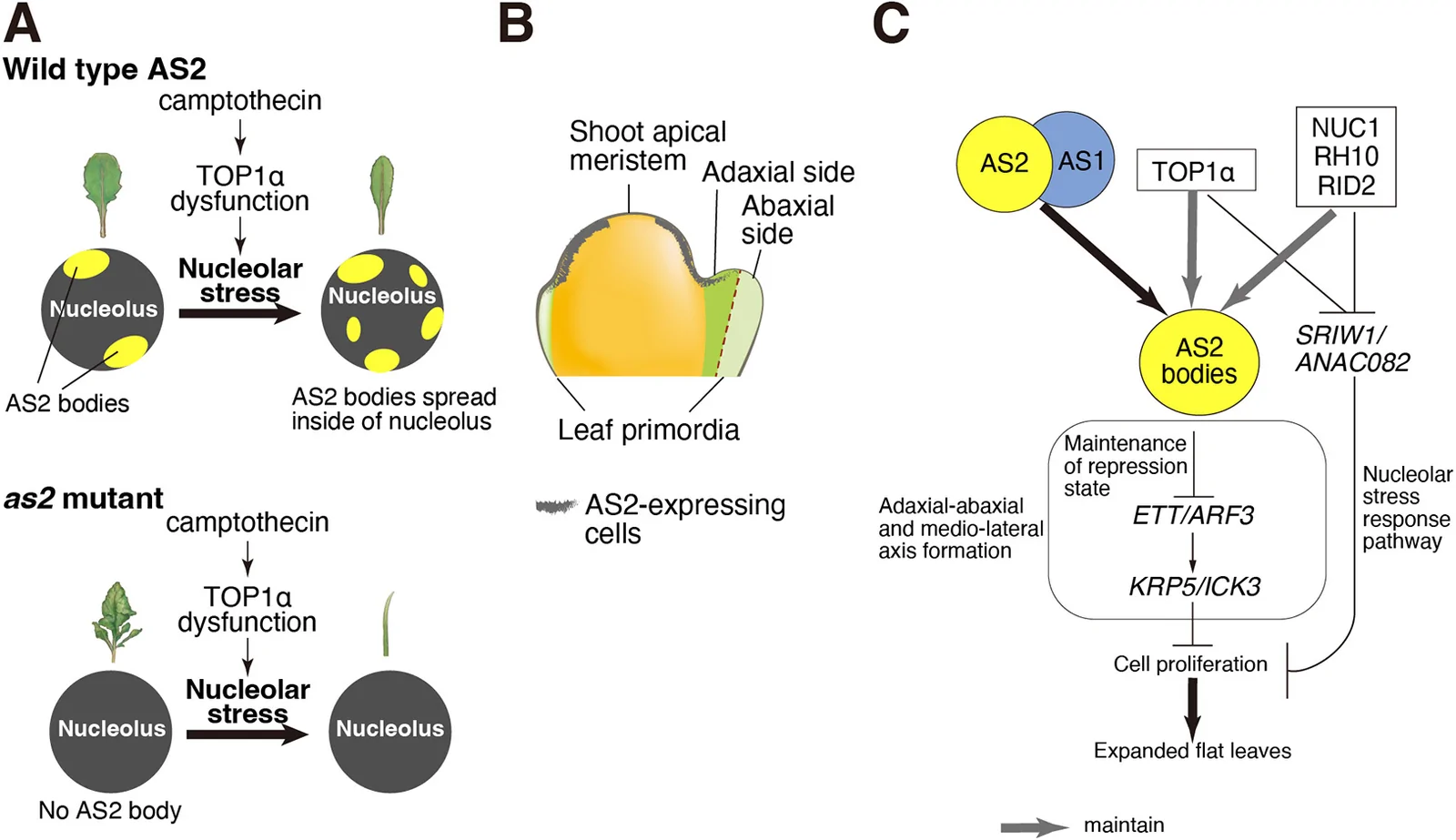
**Roles of AS2, TOP1α and nucleolar proteins in shoot apices during the formation of expanded flat leaves.** (A) Leaf phenotypes and localization of AS2 bodies in Col-0 and *as2*, when nucleolar stress is induced. (B) Developmental compartments in the shoot apex around the apical meristem are schematically shown. (C) Roles of the AS2–AS1 complex, TOP1α and nucleolar proteins (NUC1, RH10 and RID2) in the regulation of *ETT*/*ARF3* and *KRP5*/*ICK3* genes via AS2 bodies in early stages of leaf primordia for the formation of expanded flat leaves. The nucleolar stress response via *SRIW1*/*ANAC082* is shown. Gray arrows show the maintenance of AS2 bodies.

The AS2 bodies spread inside of nucleolus in the *top1α-1* plant (Figs 6D and 7A). Mutations in nucleolar factors such as *RH10*, *RID2* and *NUC1* similarly affect AS2 body patterning, further supporting a close relationship between AS2 bodies and nucleolar function (Kojima et al., 2007; Pontvianne et al., 2007; Ohbayashi et al., 2011; Matsumura et al., 2016; Luo et al., 2020; Ando et al., 2023). Previous studies in animal and yeast cells showed that TOPI participates in rDNA transcription, and that inhibition of topoisomerases induces nucleolar stress (Zhang et al., 1988; Boulon et al., 2010; Ishihara et al., 2022; Lafita-Navarro and Conacci-Sorrell, 2023). Consistent with this, the filamentous leaf phenotype of *as2-1 top1α-1* was suppressed by *sriw1/anac082*, a factor involved in the plant nucleolar stress response, suggesting that loss of *TOP1α* also induces nucleolar stress in plants. The filamentous leaf phenotypes observed in 10H-CPT-treated *as2-1* plants and in the *as2-1 top1α-1* double mutant further indicate that wild-type AS2 helps maintain leaf development under nucleolar stress conditions (Fig. 7A,C). Together, these findings suggest that AS2 functions as a factor that mitigates nucleolar stress through formation of AS2 bodies during leaf development. AS2 bodies may function in maintaining the repressed state of the *ETT*/*ARF3* gene.

The nucleolus is not only the site of ribosome assembly but also a hub for cellular stress responses (Boulon et al., 2010; Yang et al., 2018; González-Arzola, 2024). The term nucleolar stress, also known as ribosomal stress, is used to describe cellular stress that is caused by disorders of ribosome biogenesis and mostly accompanied by abnormalities of the nucleolar structure, which in turn compromise cellular homeostasis (Ohbayashi and Sugiyama, 2018). Consistent with this, mutations in genes encoding nucleolar proteins often cause narrow-leaf phenotypes, and their combinations with *as2* enhance defects in leaf development (Horiguchi et al., 2011; Matsumura et al., 2016; Ohbayashi et al., 2017).

In our study, we screened a library of natural organic compounds to identify those capable of inducing filamentous leaf morphology specifically within the *as2* mutant background. This screening effort yielded eight candidate molecules (Fig. 1A, Table 1). Upon further investigation, we discovered that molecules inducing the rod-shaped, filamentous leaves in the *as2* background share a common feature: they all exhibit antitumor activity in animal cells (Tables 1 and 2). Notably, several of these molecules have been shown to induce nucleolar stress in our system and in others, hinting at a conserved mechanism of action (Boulon et al., 2010; González-Arzola, 2024). Genetic screens using *as2* or *as1* mutants have identified more than 60 modifier genes that function together with AS2-AS1 in leaf adaxialization (Pinon et al., 2008; Yao et al., 2008; Szakonyi and Byrne, 2011; Horiguchi et al., 2011; Iwasaki et al., 2013; Machida et al., 2015; Iwakawa et al., 2020). Ribosome biogenesis may be impaired and induced nucleolar stress in a subset of these modifier mutants.

### Repression of *KRP5/ICK3* by AS2 promotes flat leaf development

We demonstrated that the *as2 top1α* double mutants produced abaxialized filamentous leaves, accompanied by elevated *KRP5/ICK3* expression (Fig. 4C). In addition, *krp5* mutations suppressed the formation of these filamentous leaves, while *CYCB1;2p:GUS* activity was reduced in the *as2 top1α* double mutants (Fig. 4G). Furthermore, *KRP5*/*ICK3* has been reported to positively regulate endoreduplication (Jégu et al., 2013; Wen et al., 2013). Therefore, AS2 may promote cell proliferation by suppressing KRP5-mediated endoreduplication. Together with previous findings (Takahashi et al., 2013), these results suggest that AS2 represses *KRP5/ICK3* expression via *ETT*/*ARF3*, thereby ensuring proper cell proliferation during early leaf development (Fig. 7C).

*Arabidopsis* contains seven *KRP*/*ICK* genes encoding Cip/Kip-type CDK inhibitors (De Veylder et al., 2001). In the root meristem, *KRP5/ICK3* contributes to meristem and stem cell maintenance and functions in the control of G1 progression (Santuari et al., 2016; Echevarría et al., 2025). These regulatory mechanisms may also operate in the shoot apex, suggesting that *KRP5*/*ICK3* could similarly suppress cell proliferation in shoot apex cells. Consequently, the repression of *KRP5/ICK3* gene expression by *AS2* and *AS1* appears essential for promoting cell proliferation following adaxial cell differentiation during early leaf formation (Fig. 7B,C).Together with the established role of AS2–AS1 in leaf polarity specification, our findings support a model in which AS2 contributes not only to leaf morphogenesis but also to the alleviation of nucleolar stress via the proper formation of AS2 bodies, thereby ensuring stable flat leaf development (Fig. 7B,C).

## MATERIALS AND METHODS

### Plant strains and growth conditions

*Arabidopsis thaliana* ecotype Col-0 (CS1092) and the mutants *as1-1* (CS3374), *as2-1* (CS3117) and SALK T-DNA insertion lines were obtained from the Arabidopsis Biological Resource Center (ABRC) (Columbus, OH, USA). Ler-0 (NW20) was obtained from the Nottingham Arabidopsis Stock Centre (Nottingham, UK; NASC). In this study, the T-DNA insertion lines SALK_069847, SALK_022236, SALK_089717 and SALK_069817 are designated as *top1β-1* (Zhang et al., 2016), *top1β-2*, *krp5-4* and *krp2-5* (Vieira et al., 2013), respectively. The genomic maps are shown in Figs S2A, S4A and S5A. The mutants *ett-13*, *bp-1 knat2-3 knat6-2*, *top1α-1*, *mgo1-7* (SALK_112625), *eal-1*, *krp5-1* (SALK_053533) and *sriw1* have been described previously (Takahashi et al., 2002; Graf et al., 2010; Ikezaki et al., 2010; Ishibashi et al., 2012; Iwasaki et al., 2013; Wen et al., 2013; Ohbayashi et al., 2017). The AS2-YFP and 35S:NUCLEOLIN1:GFP (35S:NUC1-GFP) have been described previously (Ueno et al., 2007; Luo et al., 2020; Kojima et al., 2007). Primers and combinations for detecting each mutation by genomic PCR are shown in Tables S5 and S6, respectively. Ages of plants are given in terms of the number of days after sowing (DAS).

### Chemicals

The library of molecules containing 2209 natural molecules was purchased from Inter BioScreen (Moscow, Russia). 10H-CPT and etoposide were purchased from Cosmo Bio (Tokyo, Japan) and Sigma-Aldrich (St Louis, MO*,* USA), respectively. Mitomycin C, paclitaxel, oryzalin and propyzamide were purchased from Fujifilm Wako Pure Chemical. Bleomycin A5 was purchased from LKT Laboratories. For all chemical treatments, molecules, except mitomycin C, were dissolved in dimethyl sulfoxide (DMSO; mitomycin C was dissolved in H_2_O), and each was mixed with Murashige and Skoog (MS) agar medium and immediately dispensed into plastic multi-dishes (multidish 267061, Thermo Fisher Scientific). 1,3,5(10)-estratriene-3,17 β-diol (17β-estradiol) was purchased from Sigma. The 17β-estradiol was prepared as a 100 μg/ml stock solution dissolved in DMSO. The solutions were stored at −20°C in darkness. The working solution was made by diluting the stock solution immediately before use. All chemical treatments (except 17β-estradiol) were performed by supplementing MS agar medium with the indicated compounds. Seeds were sown directly on chemical-containing MS medium and grown for 7 (for AS2-YFP and NUC1-GFP imaging, Fig. 6A,B,C,D), 13 (for *CYCB1;2p:GUS*, Fig. 4G,H, Fig. S8), 14 [for observation of *ETTp:GUS*, *BPp:GUS*, *FILp:GFP*, *krp5-1* (*krp5-4* or *krp2-5*) *as2-1* treated with 10H-CPT, all the qRTp-PCR analysis, Fig. 1F,G, Fig. S1, Fig. 4A,B, Fig. S6B, Fig. S7C,D, Fig. 1E, Fig. 5E-G, Fig. 6F-H, Fig. S3F, Fig. S7B] or 21-22 days (all the other leaf phenotypic analysis using 10H-CPT, etoposide, mitomycin C, bleomycin A5, propyzamide, oryzalin and paclitaxel, Fig. 5A-D, Fig. S9).

### Histochemical assay for GUS and GFP

Histochemical detection of GUS activity was performed essentially as described by Iwasaki et al. (2013). *BPp:GUS*/*as2-1* or 4.9 kb *ETTp:GUS*/*as2-1* (Iwasaki et al., 2013) plants were treated with and without 0.15 μM 10H-CPT for 21 days and fixed. Fixed plants were incubated in GUS staining solution described in Iwasaki et al. (2013) at 37°C for 24 h (*BPp:GUS* and *ETTp:GUS*) or 96 h (*pKRP5:KRP5-GUS*). Images of shoot apices of *pKRP5:KRP5-GUS* plants were obtained with Nikon Ti2 microscope. For the preparation of *FILp:GFP*/*as2-1* plants treated with 10H-CPT or *FILp:GFP*/*as2-1 top1α-1* plants, thin sections and cleared specimens were prepared, and GFP fluorescence images were obtained with a confocal laser microscope (Zeiss LSM510) as described previously (Nakagawa et al., 2012).

### Plasmid construction and transformation

For preparation of *BPp:GUS*, bacterial artificial chromosome (BAC) F9M13 from chromosome IV was obtained from the *Arabidopsis* Biological Resources Center. BAC DNA was digested with BamHI (at 5,3-kb upstream sequence of the *BP* gene) and NheI (at 0.3-kb downstream of translation start site of *BP* gene)*,* and the resultant 5.6-kb fragment of genomic DNA was inserted into pBluescript SK- and then the product was introduced into the binary vector pGreen Km^+^ bar^+^ with the coding sequence of β-glucuronidase. We transformed the Col-0 plants using *Agrobacterium tumefaciens* GV3101 that harbored the plasmid with the incorporated DNA fragment, as described by Bechtold and Pelletier (1998). We crossed *BPp:GUS*/Col-0 plants to *as2-1* mutant plants used for our experiments.

For preparation of *Op*TOP1-expression vector(s), *OpTOP1* cDNA from the entry vectors pDONR221-OpTOP1 (Sirikantaramas et al., 2008) was transferred to the binary T-DNA destination vector pGWB2 (Nakagawa et al., 2007) in reactions mediated by LR clonase (Thermo Fisher Scientific). We transformed the *as1-1* plants using *Agrobacterium tumefaciens* GV3101 that harbored the resultant recombination constructs, *35Sp:OpTOP1* with the incorporated DNA fragment, as described by Bechtold and Pelletier (1998).

For construction of *CYCB1;2p:GUS,* the promoter fragment (1.0 kb) from the *CYCB1;2* gene was cloned into the entry vector pENTR-D/TOPO (ThermoFisher), and the resulting plasmid was subjected to LR reaction with the destination vectors pBGGUS (Kubo et al., 2005) to create *CYCB1;2p:GUS*. The construct was introduced by floral-dip transformation (Clough and Bent, 1998) into *Arabidopsis* (Col-0) plants. For cloning of the promoter fragment from *CYCB1;2* gene locus, the primers Cyc1b fullpro5/CACC: CACCATCGTGAAGGTAACATTTACAAC and Cyc1b-p3: TTCTCTTTCGTAAAGAGTCTCTGCG were used. We crossed a *CYCB1;2p:GUS*/Col-0 transgenic line with *as2-1*, *top1α-1* and *krp5-1*.

For construction of *pKRP5:KRP5-GUS*, the fragment containing the promoter sequence (2987 bp) and coding region (750 bp) was fused to *GUS* reporter gene. The construct was introduced by floral-dip transformation into *Arabidopsis* (Col-0) plants.

### Quantification of GUS activity

The GUS signal intensity of shoot apices from GUS stained images was measured using Image J software as described previously (Béziat et al., 2017). In brief, the images of shoot apices were cropped and converged to Hue Saturation Brightness (HSB) mode, and the GUS signal intensity was measured in the Saturation channel. The quantitative GUS assay for detecting 4-methylumbelliferone (4-MU) fluorescence was performed as described previously (Halder and Kombrink, 2015; Iwakawa et al., 2021), with some modifications. The aerial part from 13-day-old *CYCB1;2p:GUS* seedlings grown in 1/2MS medium was harvested in zirconia beads-containing 2 ml tubes, crushed with Tissue lyzer, resuspended with 200 μl of extraction buffer containing 100 mM sodium phosphate (pH 7.0) and 1 mM dithiothreitol, and centrifuged at 20,000 ***g*** at 4°C for 20 min. After determining the protein concentrations of the supernatants, 160 μl of the supernatant (corresponding to 30 μg protein) was transferred to a 96-well transparent plate and mixed with GUS assay solution (40 μl) containing 5 mM 4-methylumbelliferyl- β-D-glucuronide (M3026, Tokyo Chemical Industry), 125 mM sodium phosphate (pH 7.0), 2.5 mM EDTA, 0.25% Triton X-100 and 25 mM β-mercaptoethanol. Samples were incubated at 37°C for 60 min, and 4-MU fluorescence was measured every 10 min using the plate reader Spark (TECAN Group, Männedorf, Switzerland) with an excitation/emission wavelength of 365/455 nm. The GUS activity was calculated using the ΔE455 increments (10-120 min).

### Real-time quantitative reverse transcriptase-polymerase chain reaction

Each 30 shoot apices from 14-day-old plants were collected, and total RNA was extracted with the RNeasy Plant Mini Kit (Qiagen) according to the manufacturer's instructions. Sample volumes were normalized as described by Nakagawa et al. (2012). The primer pairs are shown in Table S7. For the analysis of RNA levels in *Arabidopsis* by real-time quantitative reverse transcriptase-polymerase chain reaction (qRT-PCR), we prepared 10 μg of total RNA. Reverse transcription was carried out with ReverTra Ace (TOYOBO, Osaka, Japan). PCR was performed in the presence of the double-stranded DNA-specific dye SYBR Green (Thermo Fisher Scientific). Amplification was monitored in real time with the Applied Biosystems StepOnePlus Real-Time PCR system (Thermo Fisher Scientific) according to the supplier's recommendations. The mean values of three technical replicates were normalized by using the *ACTIN2* (*ACT2*) or *TRANSLATION ELONGATION FACTOR* (*EF-1α*) transcripts as each control.

### Introduction of AS2-YFP into *top1α-1*

We crossed the Col-0 background AS2-YFP stamens with *top1α-1* pistils. The F_1_ seeds were selected on MS medium that contained hygromycin. The F_2_ plants were then selected on MS medium supplemented with hygromycin, based on leaf phenotype, via PCR. The appropriate pairs of primers for each mutant that we used for the genotyping are listed in Table S7. We used the F_3_ plants for the AS2-YFP observation.

### Observation of fluorescence

To examine the subcellular localizations of the YFP fused to AS2, we established three lines of transgenic *Arabidopsis* plants. To induce the expression of the transgenes, the 6 DAS transgenic *Arabidopsis* whole plants were soaked in 0.05 μg/ml of 17β-estradiol in 1× MS medium under vacuum conditions for 30 min and were then incubated at 22°C overnight. The patterns of fluorescence due to YFP were analyzed in 11 to 31 cells of each line of transgenic plants after the induction of expression of transgenes. YFP-positive nuclei were observed in the adaxial epidermis of the cotyledons. Images were recorded via confocal laser scanning fluorescence microscopy with an Objective Plan-Apochromat 63×/1.4 Oil DICII and an Objective α Plan-Apochromat 100×/1.46 Oil DIC (LSM710; Zeiss). We acquired *z*-stack images of the AS2 bodies using Zen 2012 software (Carl Zeiss). The YFP signal was detected with excitation at 514 nm and an emission bandpass filter from 519 nm to 620 nm. Raw data were collected at a resolution of 1024×1024 pixels.

For NUC1-GFP, images were acquired using a STELLARIS 5 WLL microscope (Leica) equipped with a 63× objective lens. The GFP signal was detected with excitation at 489 nm and emission spectral window of 494 nm to 560 nm. Raw data were collected as 2048×2048 pixel images.

### Microarray

Shoot apices from seedlings of wild-type and *as2-1* plants grown with and without 0.15 μM 10H-CPT for 14 days were harvested and total RNA was extracted as described in the ‘Real time quantitative reverse transcriptase-polymerase chain reaction’ section. For microarray analysis, the quality and purity of the RNA were confirmed with an Ultrospec 2100 pro (GE Healthcare). Total RNA samples (100 ng) were reverse transcribed, yielding double-stranded cDNA that was transcribed *in vitro* in the presence of biotin-labeled nucleotides with an IVT Labeling Kit (Affymetrix) and purified. Labeled cRNA was fragmented and hybridized to Affymetrix ATH1 GeneChip arrays for 16 h at 45°C according to Affymetrix protocols. Arrays were washed on an Affymetrix Fluidics Station 450 and measured for fluorescence intensity with an Affymetrix GeneChip Scanner 3000. The raw data were processed by using Affymetrix Gene Chip Operating Software (GCOS; Version 1.4.0.036).

### Microarray data processing

The raw data were processed by using Affimetrix Gene Chip Operationg Software (GCOS; Version 1.4.0.036). Each expression data set includes 22,746 *Arabidopsis* genes (probe set). Initially, we calculated the expression signal for all data sets and the log2 fold changes (FC) for Col-0+10H-CPT, *as2-1*+DMSO and *as2-1*+10H-CPT against the Col-0+DMSO by GCOS. In this experiment, we excluded 64 controls and 2177 genes subject to cross-hybridization, according to NetAffx Annotation (www.affymetrix.com). We also excluded genes for which all datasets showed an absent call (i.e. the detection call determined by GCOS based on the P-value of the one-sided Wilcoxon signed-rank test; an absent call means *P*≥0.065, which is the default threshold in GCOS), because it indicates that the expression signal was undetectable. Furthermore, we excluded genes for which all datasets showed a no change call [i.e. no change call means (1–0.006)≥*P*≥0.006 for one-sided Wilcoxon signed-rank test], because no change indicates that the expression signal is almost equal to that of Col-0+DMSO. Thus 8009 genes were chosen. Among these data, the log2 FC values that were >1 or <−1 were rounded to 1 and −1, respectively; the log2 FC values with no change were rounded to 0, to avoid category proliferation in clustering. We hired KB-FuzzyART for clustering as described previously (Takahashi et al., 2013). First, for KB-FuzzyART, FuzzyART was applied to any knowledge composed of 486 genes listed in Gene-list-4, previously published (Tomida et al., 2002; Matsumura et al., 2016; Table S1). The KB-FuzzyART parameters (i.e. vigilance parameter and learning rate) were optimized by using an optimal clustering index (OCI) (Takahashi et al., 2003), and then 30 clusters were constructed. Weight vectors (representative patterns for each cluster) for the 30 clusters were constructed by FuzzyART, and the learning rate was set to 0, to fix the constructed weight vectors. FuzzyART with 30 fixed weight vectors was then applied to the 8009 filtered genes for each dataset. All genes were assigned to each cluster or outliers (160 genes). The outliers represent genes with patterns that did not match patterns from Gene-list-4. In Fig. S3B,D and Tables S2-S4, the gene enrichment rate for categories designated in Gene-list-4 [‘cell fate (cell fate determination-related genes)’, ‘cell division (cell division-related genes)’, ‘plant hormone (plant hormone-related genes)’, ‘small RNA (small RNA biogenesis-related genes)’] among 8009 genes were calculated in each Clusters and mixed Clusters. FDR values were calculated with the Benjamini-Hochberg method using *P*-values (Fisher's exact test).

### scRNA-seq data processing

The published scRNA-seq dataset (BioProject PRJCA003094) was downloaded from the Beijing Institute of Genomics Data Center (Zhang et al., 2021). This dataset comprises four sets of FASTQ files derived from shoot apices of 7-day-old seedlings (replicates 1 and 2), 14-day-old shoot apices, and the third leaves of 18-day-old plants. The raw data were processed using Cell Ranger v.10.0.0 (10x Genomics). The *Arabidopsis* genome sequence and GTF annotation files were obtained from EnsemblPlants (https://plants.ensembl.org/index.html/). To avoid potential errors caused by redundant “gene_names” attributed in the GTF file (column 9), all “gene_name” entries were removed. Consequently, Cell Ranger utilized “gene_id” (e.g. AT1G65620), which provides non-redundant gene locus identifiers. A filtered GTF was generated using “cellranger mkgtf” function with the “--attribute=gene_biotype:protein_coding” argument. The reference genome was then constructed via “cellranger mkref” function with “--genome, --fasta, and --genes” arguments. Single-cell gene expression matrices were generated using “cellranger count” function with “--id, --fastqs, --sample, --transcriptome, and --create-bam true” arguments. All datasets were integrated using “cellranger aggr” function.

Downstream analyses were performed using the Seurat R package (v.5.4.0). For quality control, low-quality cells and genes were filtered following the criteria described by Zhang et al. (2021), as summarized below: (1) cells with fewer than 500 or more than 10,000 detected genes were excluded; (2) cells with unique molecular identifier (UMI) counts outside the range of 500 to 50,000 were removed; (3) genes expressed in fewer than three cells were discarded; and cells with (iv) mitochondrial and (v) plastid gene contents exceeding 5% and 10%, respectively, were filtered out.

For data normalization, the “NormalizeData” function was employed using the LogNormalize method with a scale factor of 10,000. Variable genes (2000 features) were identified via the “FindVariableFeatures” function with the vst method. After data scaling using “ScaleData” function, principal component analysis (PCA) was performed using “RunPCA” on the variable features. Cell clustering was conducted using “FindNeighbors” and “FindClusters” functions based on the Louvain algorithm to construct a shared nearest neighbor (SNN) graph. Dimensionality reduction was visualized via Uniform Manifold Approximation and Projection (UMAP) using “RunUMAP” function, and gene expression patterns were visualized using the “FeaturePlot” function.

For the co-expression analysis shown in Fig. 3C-F, cells expressing at least one count of *KRP5*/*ICK3*, *ETT/ARF3*, *BP* or *AS2* were extracted to create individual sub-datasets. The raw counts for each subset were then used to construct new Seurat objects, which were processed following the analytical procedures described above. For Fig. 3G, the proportion of cells expressing *KRP5*/*ICK3*, *ETT/ARF3*, *BP*, *AS2* or *TOP1α* (count≥1) within each subset was calculated to determine co-expression ratios.

### MM2d cells and synchronization

A suspension culture of *Arabidopsis thaliana* cell line MM2d (L*er* ecotype) (Menges and Murray, 2002) was maintained at 25°C in the dark by weekly subculturing in modified Linsmaier and Skoog medium (Haga et al., 2007) with rotation at 120 rpm. To synchronize the MM2d cell cycle at the G1/S phase boundary, 40 ml of a 7-day-old cell culture were transferred to 160 ml of modified Linsmaier and Skoog medium containing 5 mg/l aphidicolin (Fujifilm Wako Pure Chemicals). After culturing for 24 h, cells were washed and cultured further in 200 ml of fresh medium. Every 2 h after the drug removal, cells were harvested by centrifugation, frozen in liquid nitrogen and stored at −80°C until use. RNA was extracted with Trizol reagent according to the manufacturer's instructions (Thermo Fisher Scientific). Purification of Poly(A)^+^ RNA and synthesis of first-strand cDNA were performed as described previously (Iwakawa et al., 2007).

## Supplementary Material



10.1242/develop.205275_sup1 Supplementary information
