# Supplementary material for: Arabidopsis epigenetic factor AS2 mitigates nucleolar stress and promotes flat leaf development by repressing a CDK inhibitor
Source: Development. 2026 Sep 18;153(18):dev205275. doi: 10.1242/dev.205275 (PMC13680833; doi:10.1242/dev.205275)
Supplement: Supplementary file 1 [file develop-153-205275-s1.pdf]

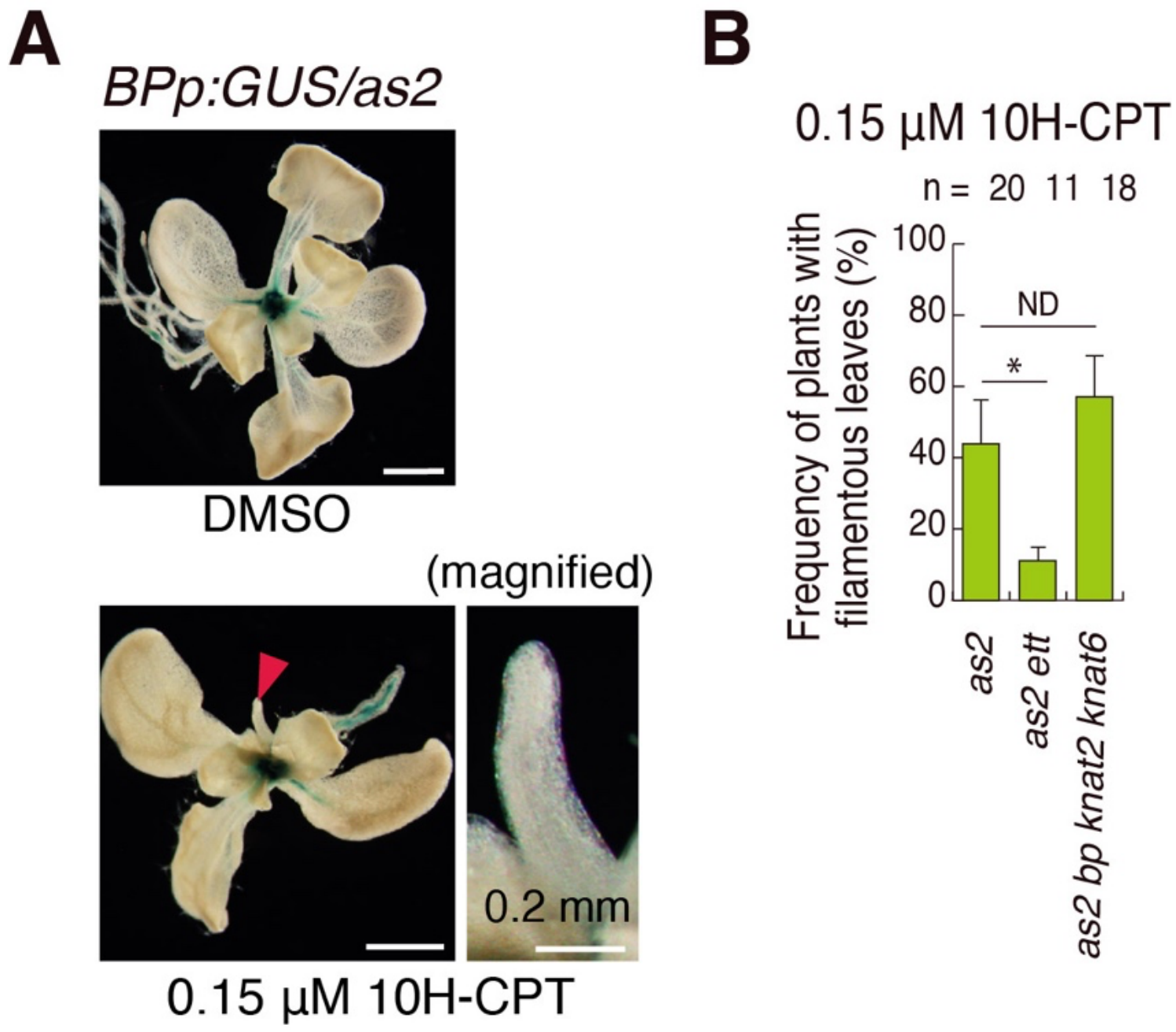

Fig. S1. Mutations of class 1 *KNOX* genes did not suppress the phenotype of filamentous leaves.

(A) Typical *BPp:GUS*/*as2-1* plants treated with 0.15 µM 10H-CPT from sowing for 14 days. Bars = 1 mm. (B) Frequencies of plants with filamentous leaves at positions of 1st and 2nd leaves when plants were treated with 0.15 µM 10H-CPT. (p<0.05, Tukey's HSD test after significant ANOVA).

Development • Supplementary information

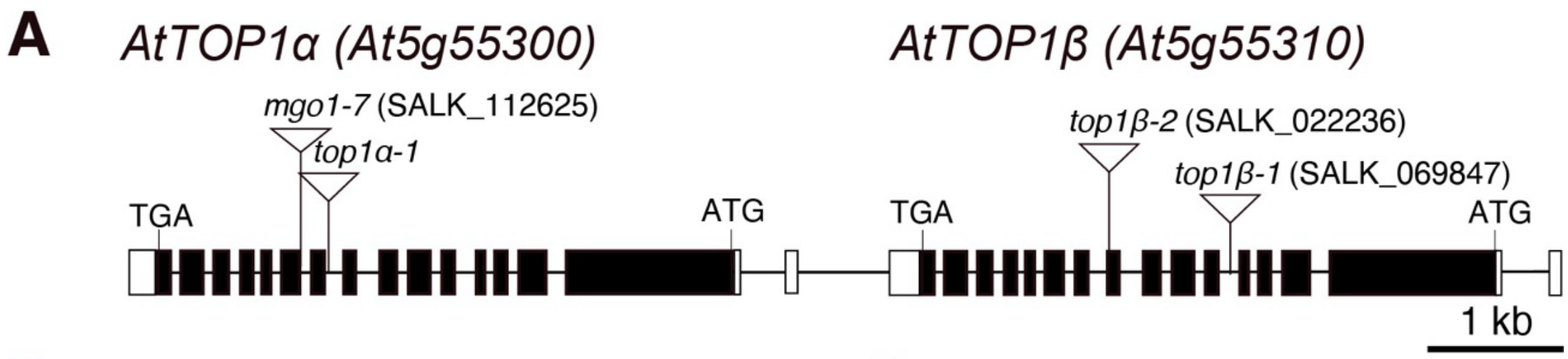

B

	Frequency of plants (%)				
	with expanded leaves	with filamentous leaves	without true leaves	lethality	*n*
Col-0	100	0	0	0	10
as2-1	100	0	0	0	10
top1α-1	100	0	0	0	100
mgo1-7	72	1	5	22	101
as2-1 top1α-1	19	21	31	29	86
as2-1 mgo1-7	50	10	9	31	101
as2-1 top1α-1 ett-13	94	6	0	0	17
as1-1	100	0	0	0	10
as1-1 top1α-1	50	10	20	20	79
as1-1 mgo1-7	76	3	4	17	101
as1-1 top1α-1 ett-13	100	0	0	0	19

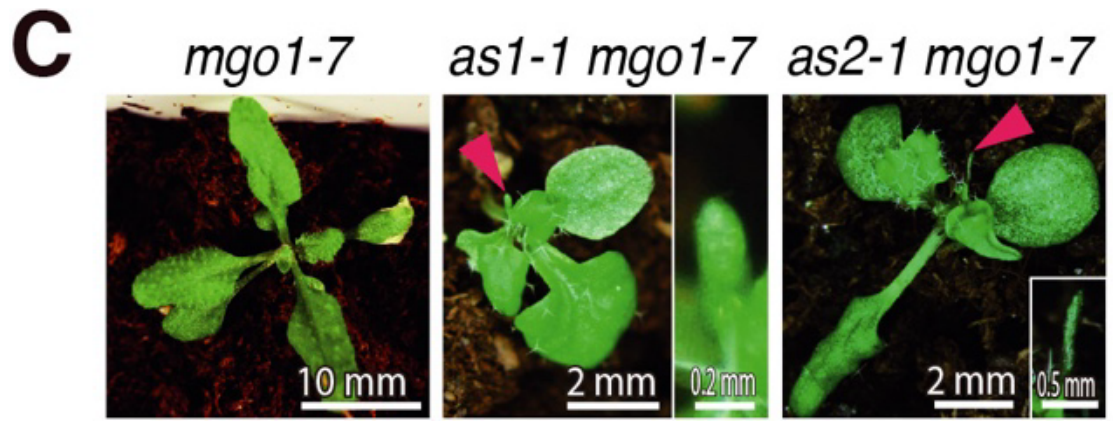

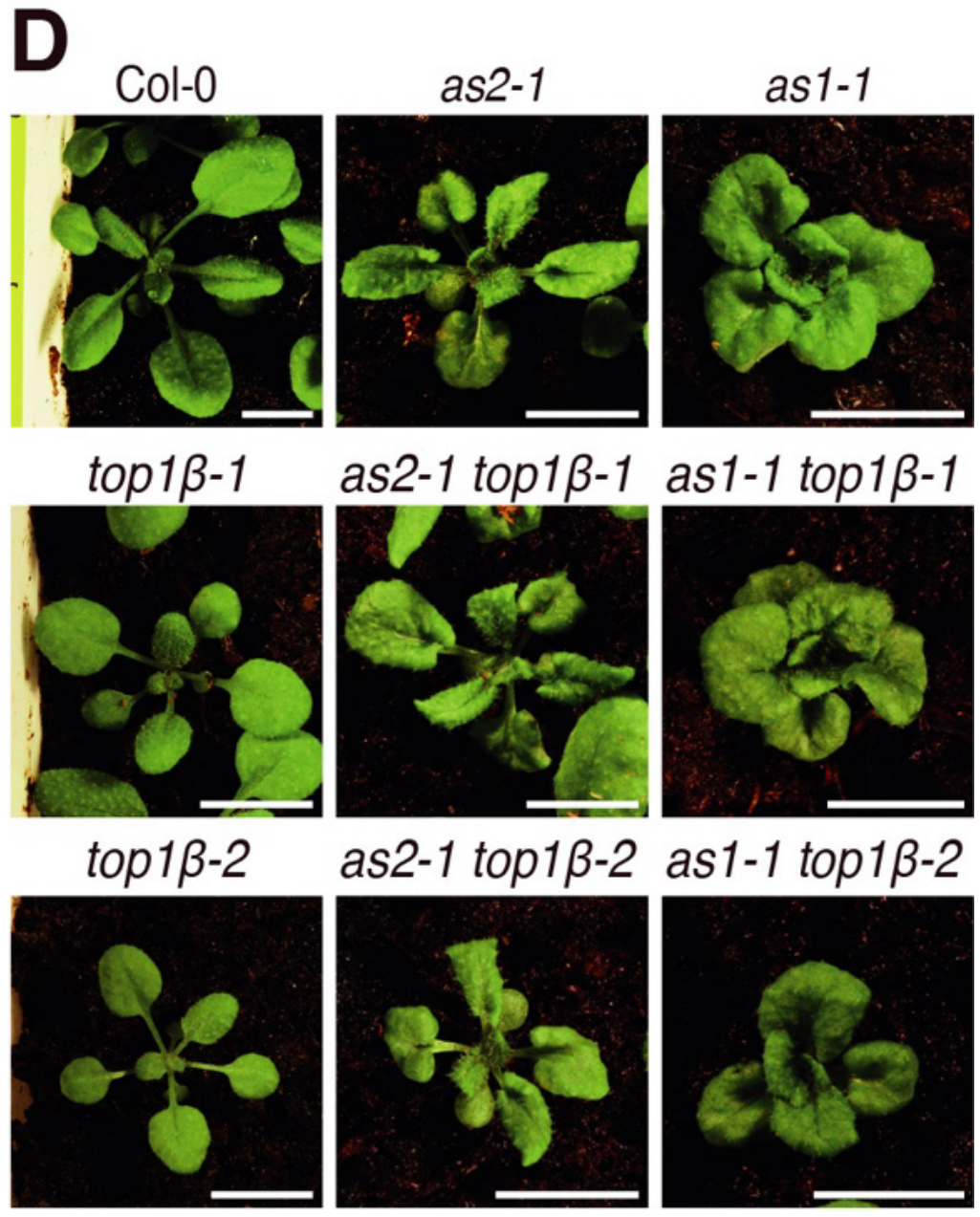

Fig. S2. Mutation of *TOP1α*/*MGOUN1* (*MGO1*) enhanced the leaf abaxialization of *as1* and *as2*, but mutation of *TOP1β* did not.

(A) Genomic structures (exon-intron organizations) of type IB DNA topoisomerases, *AtTOP1α*/*MGO1* and *AtTOP1β*. Black boxes indicate exons, and white boxes indicate untranslated exons. Sites of T-DNA insertions in mutants used in this study are indicated. (B) The *top1α-1*/*mgo1-7* mutation in *as2* or *as1* background exhibited enhanced formation of filamentous/trumpet-shaped leaves. Filamentous leaves and other abnormal shapes of leaves such as trumpet-shaped leaves were counted in the indicated genetic backgrounds. The *ett-13* mutation suppressed the filamentous leaves phenotype. (C) The mutation of *mgo1-7* exhibited impairment of leaf adaxial/abaxial polarity in *as1* and *as2* backgrounds. The phenotypes of plants grown in soil for 21 days are shown. Red arrowheads indicate filamentous leaves. (D) Genetic interactions between *AS1*, *AS2*, and *AtTOP1β*. The phenotypes of plants grown in soil for 21 or 22 days are shown. Bars = 10 mm.

Development • Supplementary information

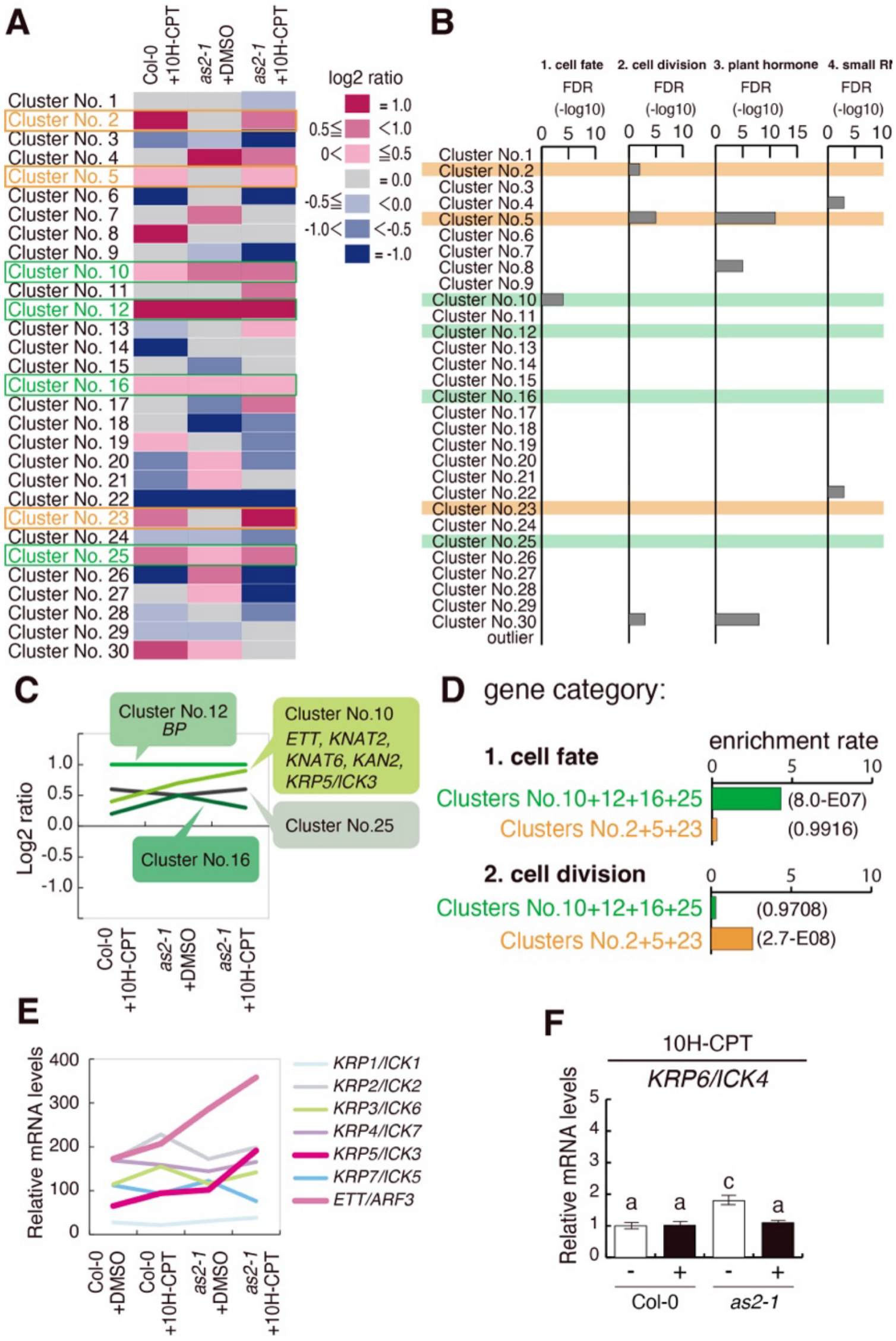

Fig. S3. The microarray and KB-FuzzyART classified 30 clusters and the *KRP5/ICK3* gene is the only gene that is categorized as a cell division gene.

(A) Clustering analysis by KB-FuzzyART. Levels of gene expression in comparison with those of Col-0 treated with DMSO are shown. (B) Gene enrichment analysis. Enrichment rates for 2 categories (cell fate and cell division) in gene list-4 among 8,009 genes were calculated. Values indicate $-\log_{10}$ q. (C) The expression patterns for cluster that includes *ETT/ARF3* (cluster 10) and those for similar expression patterns (clusters 12, 16, and 25). (D) Gene enrichment analysis of clusters 10 + 12 + 16 + 25 and 2 + 5 + 23. Enrichment rates were calculated for 2 categories (cell fate and cell division) in gene list-4 among 8,009 genes. Numbers in parenthesis indicate q values. (E) The transcript levels of *ETT/ARF3* and *KRP/ICK* genes (except *KRP6/ICK4*) in shoot apices of plants treated with and without 10H-CPT. (F) The transcript levels of *KRP6/ICK4* in shoot apices of plants treated with and without 10H-CPT from sowing for 14 days ($p<0.05$, Tukey's HSD test after significant ANOVA).

Development • Supplementary information

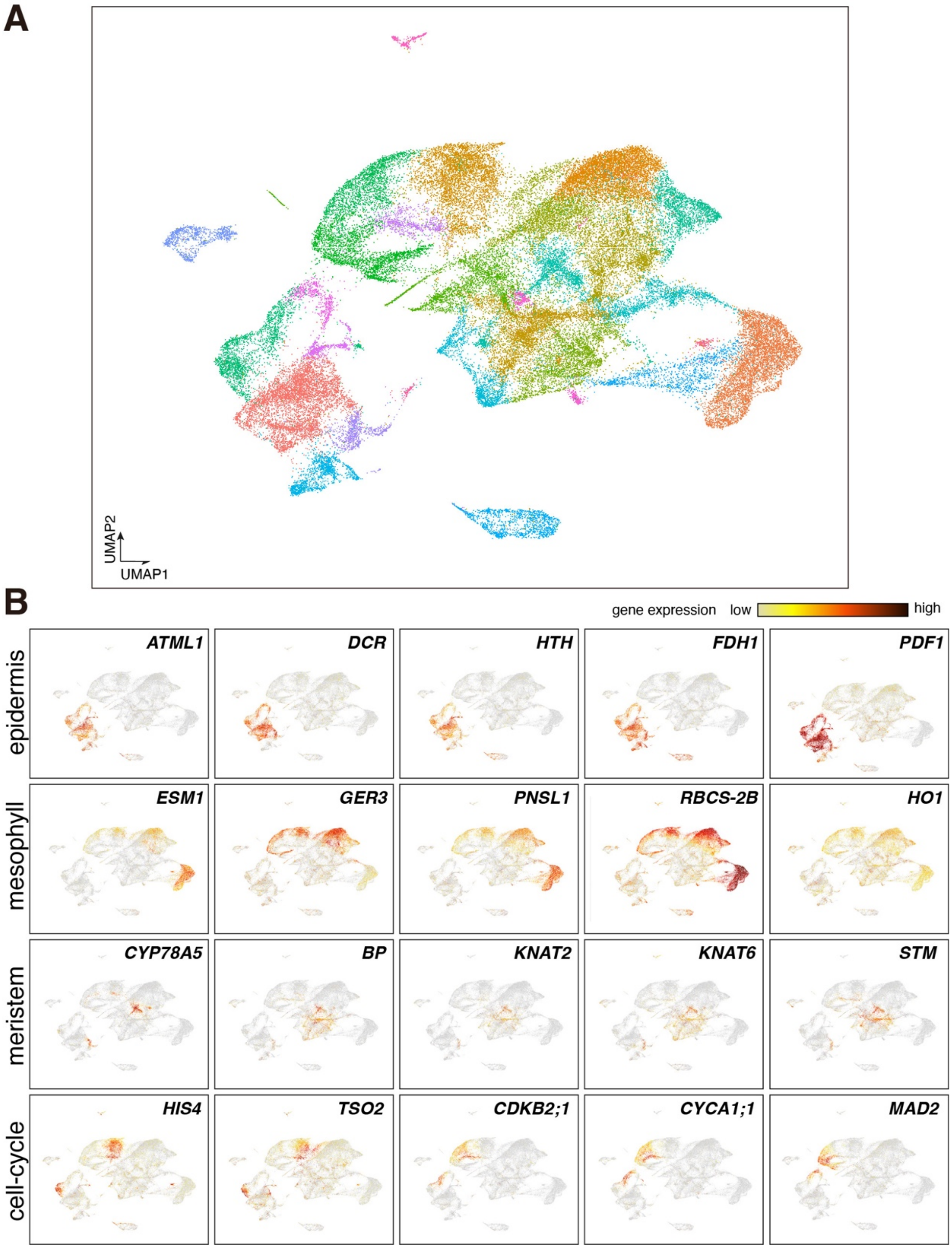

Fig. S4. Integrated UMAP visualization showing cell clusters.

(A) The dataset includes cells merged from shoot apices of 7-day-old seedling, 14-day-old shoot apices, and the third leaves of 18-day-old plants. **(B)** UMAP plots showing the expression patterns of marker genes for the epidermis, mesophyll, meristematic tissues, and the cell cycle. The color scale for gene expression levels is indicated at the top.

Development • Supplementary information

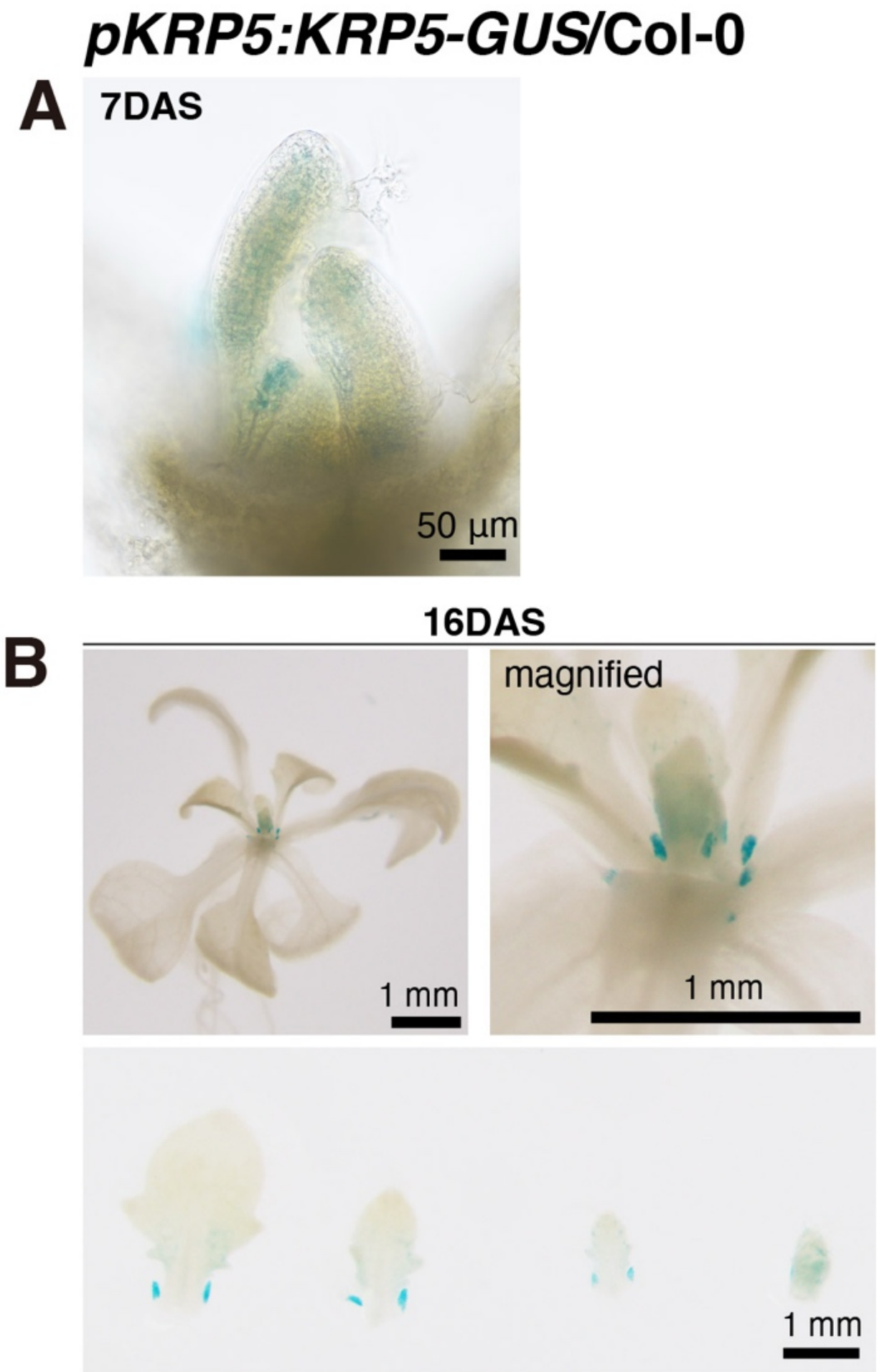

Fig. S5. The GUS expression in 7-day- or 16-day-old *pKRP5:KRP5-GUS* plants.

(A) A representative photo of meristem and early leaf primordia in 7-day-old *pKRP5:KRP5-GUS*/Col-0 plants grown on MS medium. (B) A representative photo of meristem and seedling in 16-day-old *pKRP5:KRP5-GUS*/Col-0 plants grown on MS medium (upper left), magnified views around shoot apices (upper right), and expanded leaves (bottom). A strong staining is also observed in the stipules (A and B).

Development • Supplementary information

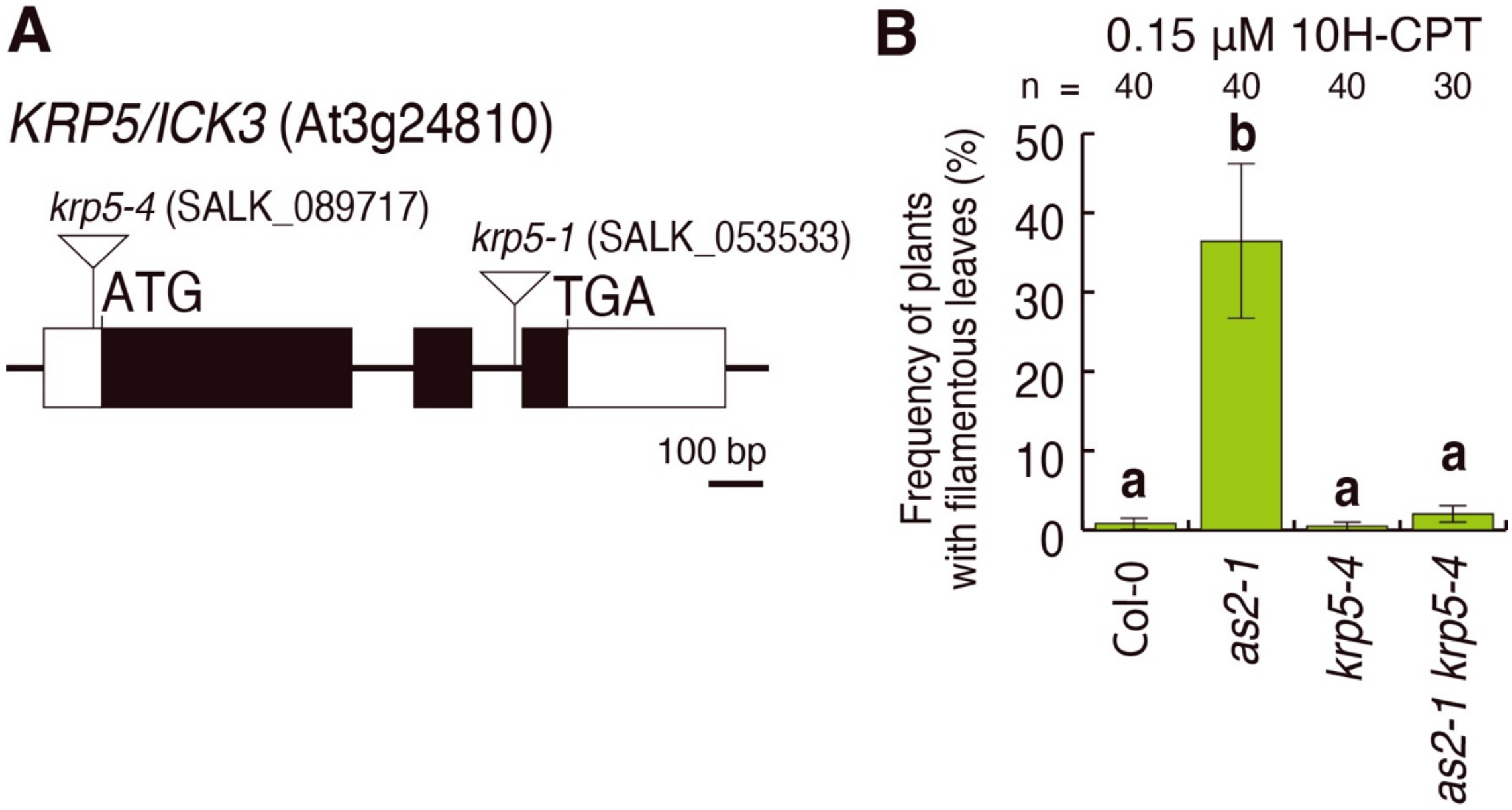

Fig. S6. The *KRP5/ICK3* gene is responsible for the formation of filamentous leaves in 10H-CPT-treated *as2*.

(A) Genomic structures of *KRP5/ICK3*. Black boxes indicate exons, and white boxes indicate untranslated exons. Sites of T-DNA insertions of mutants used in this study are indicated. (B) Frequencies of *krp5-4* plants with filamentous leaves when plants were treated with 0.15 µM 10H-CPT from sowing for 14 days. Data represent mean ±SD of 3 replicates (n=40 or 30 for each). $p<0.05$, Tukey's HSD test after significant ANOVA.

Development • Supplementary information

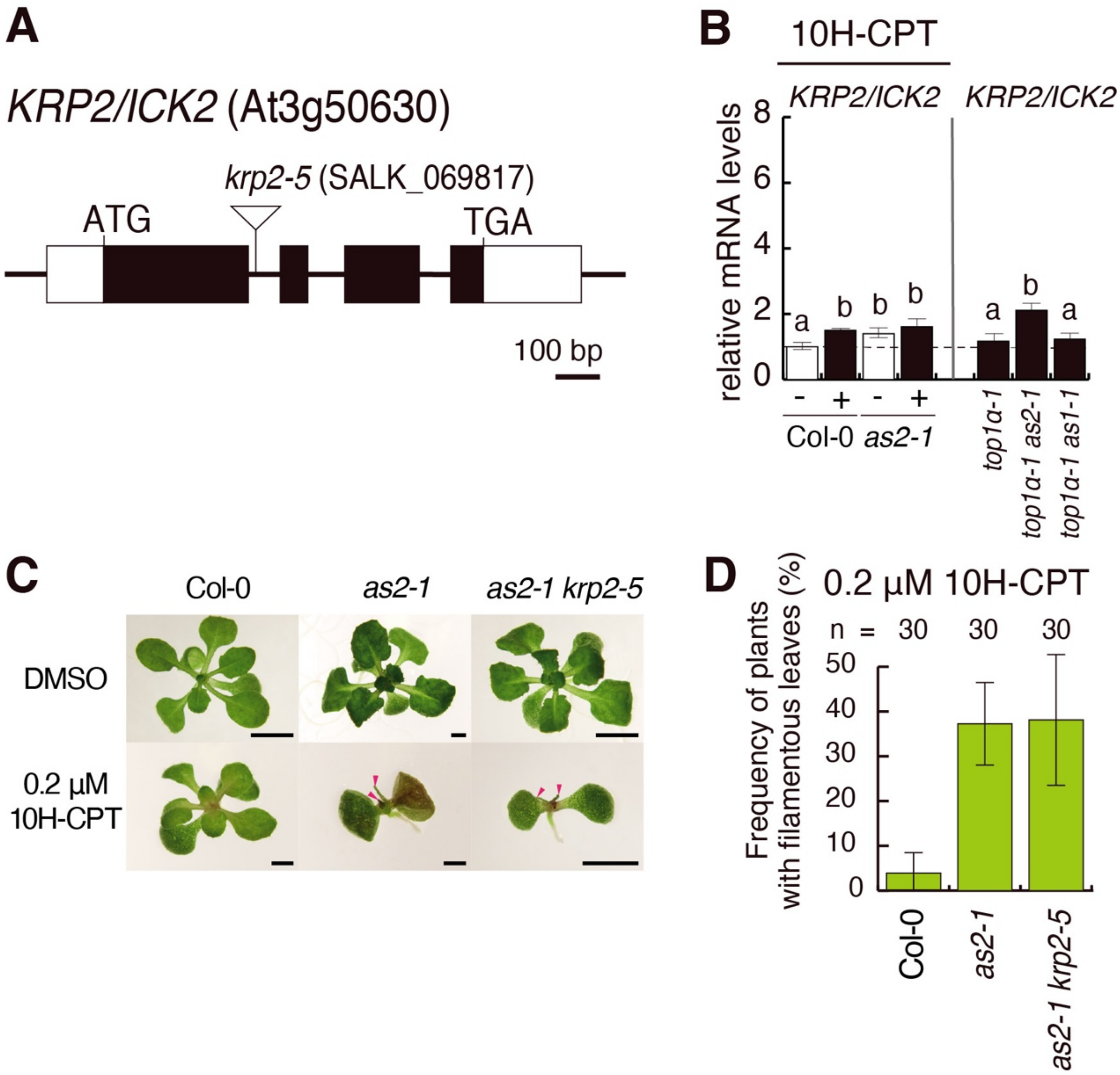

Fig. S7. The *KRP2/ICK2* gene was not responsible for the formation of filamentous leaves in *top1α as2* and 10H-CPT-treated *as2*.

(A) Genomic structures of *KRP2/ICK2*. Black boxes indicate exons, and white boxes indicate untranslated exons. Sites of T-DNA insertions of mutants used in this study are indicated. (B) Changes in transcription levels of *KRP2/ICK2* in shoot apices of Col-0 and as2-1 plants treated with and without 10H-CPT from sowing for 14 days, or shoot apices of Col-0, *top1α-1*, *as2-1 top1α-1* and *top1α-1* plants grown on soil for 14 days. Letters above bars indicate significance among transcript of each gene (p<0.05, Tukey's HSD test after significant ANOVA). (C) Phenotypes of plants treated with 0.2 µM 10H-CPT from sowing for 14 days. Bars = 1 mm. Red arrowheads indicate filamentous leaves. (D) Frequencies of plants with filamentous leaves when plants were treated with 0.2 µM 10H-CPT. Data represent mean ±SD of 3 replicates (n=30 for each).

Development • Supplementary information

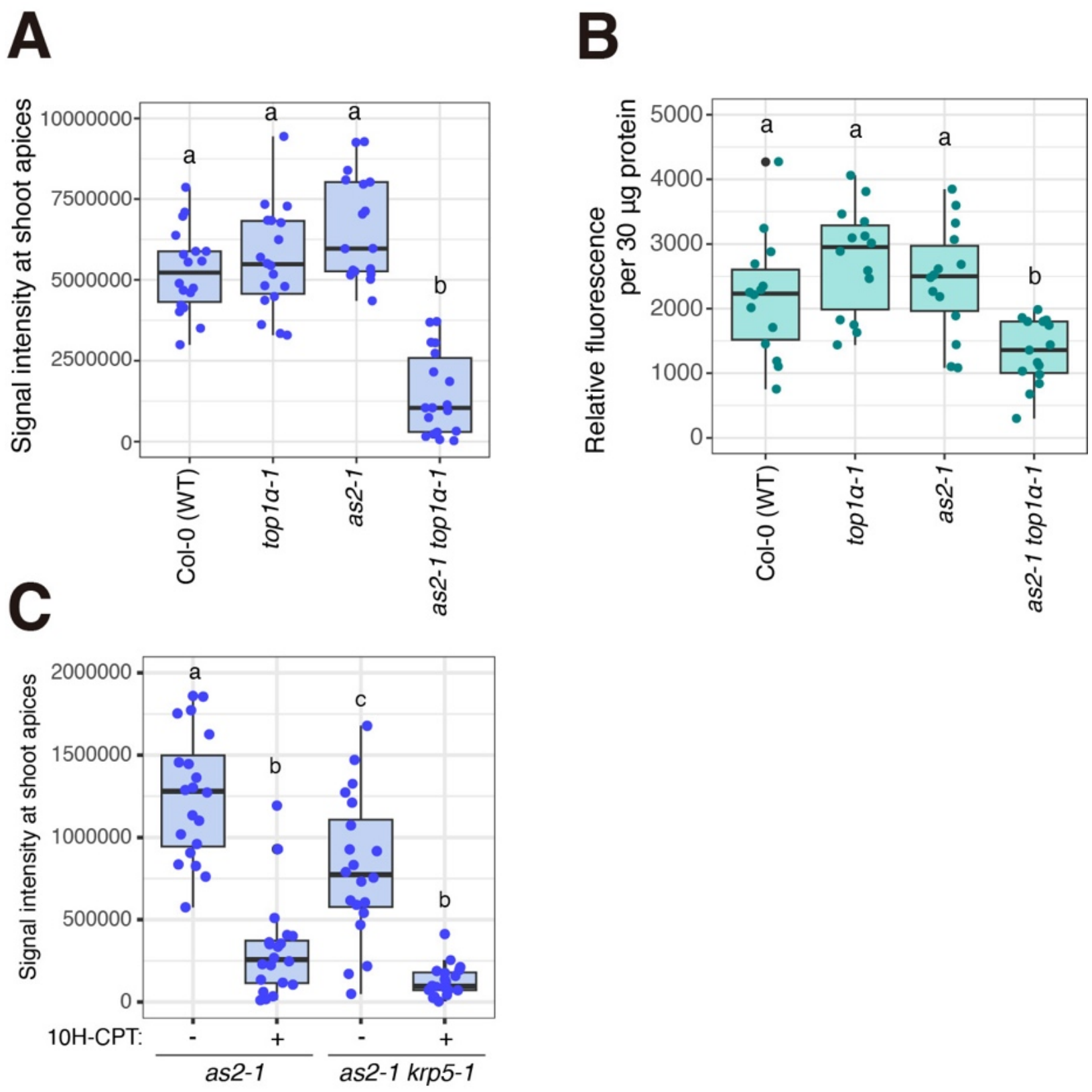

Fig. S8. Quantitative *CYCB1;2p:GUS* activity.

(A) Relative GUS activity around shoot apices in Col-0, *as2-1*, *top1α-1* and *as2-1 top1α-1* plants grown in MS medium for 13 days using X-Gluc calculated by ImageJ. One-way ANOVA followed by Tukey's HSD analysis was performed. Letters (a, b) indicate that the signal intensities are statistically different within the group. (p<0.01) n= 18. (B) Relative GUS activity from aerial part using 4-MU. Crude protein was collected from aerial part of 13-day-old *CYCB1;2p:GUS*/Col-0, *CYCB1;2p:GUS*/*as2-1*, *CYCB1;2p:GUS*/*top1α-1* or *CYCB1;2p:GUS*/*as2-1 top1α-1* plants. One-way ANOVA followed by Tukey's HSD analysis was performed. Letters (a, b) indicate that the fluorescence is statistically different within the group. (p <0.01) n = 15. (C) Relative GUS activity around shoot apices in *CYCB1;2p:GUS*/*as2-1* and *CYCB1;2p:GUS*/*as2-1 krp5-1* plants treated with 0.15 µM 10H-CPT from sowing for 13 days using X-Gluc calculated by ImageJ. One-way ANOVA followed by Tukey's HSD analysis was performed. Letters (a, b) indicate that the signal intensities are statistically different within the group. (p <0.05) n= 20.

Development • Supplementary information

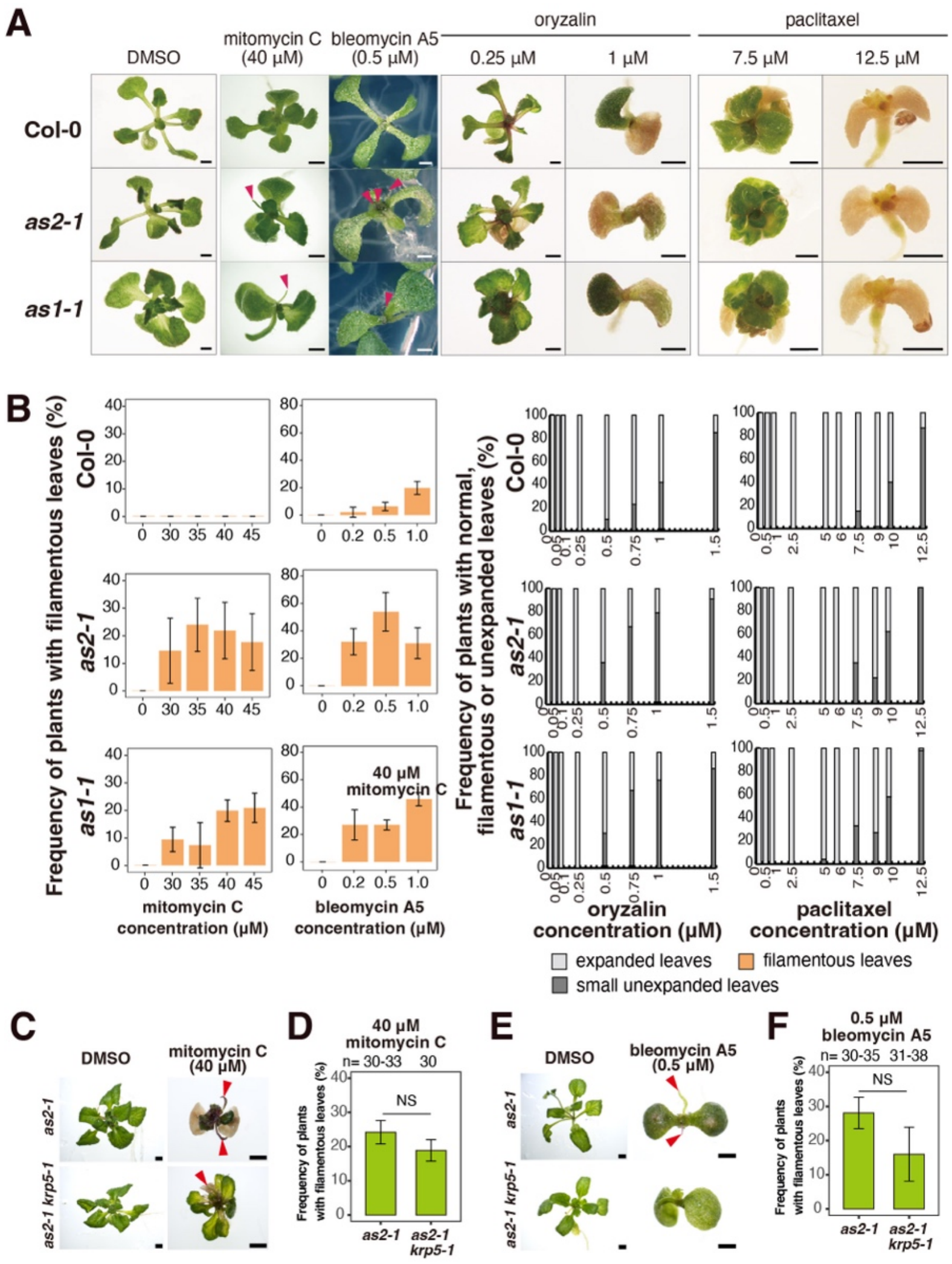

Fig. S9. Inhibitors of DNA replication induce formation of filamentous leaves, but treatment with oryzalin and paclitaxel did not exhibit defects in leaf adaxial/abaxial polarity. (A) Representative phenotypes of plants treated with mitomycin C, bleomycin A5, oryzalin, or paclitaxel from sowing for 21 days. Red arrowheads indicate filamentous leaves. Bars = 1 mm. (B) Dose-dependent effects of mitomycin C, bleomycin A5 oryzalin and paclitaxel on leaf abnormality and leaf formation. Each value is calculated from the means of 3 biological replicates (n=20 for each). (C) Representative phenotypes of *as2-1* and *as2-1 krp5-1* plants treated with mitomycin C from sowing for 22 days. Bars = 1 mm. (D) Frequencies of plants with filamentous leaves in *as2-1* and *as2- 1 krp5-1* plants treated with 40 µM mitomycin C from sowing for 22 days. $p>0.05$ for Student's t test. NS, not significant. Each value is calculated from the means of 3 biological replicates (n=30 for each). (E) Representative phenotypes of *as2-1* and *as2-1 krp5-1* plants treated with 0.5 µM bleomycin A5 from sowing for 23 days. Bars = 1 mm. (F) Frequencies of plants with filamentous leaves in *as2-1* and *as2-1 krp5-1* plants treated with bleomycin A5. $p>0.05$ for Student's t test. Each value is calculated from the means of 3 biological replicates (n=30 for each). NS, not significant.

Table S1. The constructed clusters and assignment of genes for Dataset-C

Cluster number	No. of genes	No. of genes in Gene list-4	Categories in Gene list-4				Pattern		
			1. cell fate	2. cell division	3. plant hormone	4. small RNA	Col-0 10H-CPT	*as2-1* DMSO	*as2-1* 10H-CPT
1	956	15	5	5	3	2	0	0	-
2	205	7	0	6	1	0	+	0	+
3	160	7	2	0	5	0	-	-	-
4	145	10	2	0	8	0	0	+	+
5	1507	35	3	24	7	1	+	0	+
6	267	8	1	0	7	0	-	0	-
7	246	10	2	0	7	1	0	+	0
8	151	4	0	3	1	0	+	0	0
9	181	10	4	1	4	1	0	-	-
10	140	9	7	1	1	0	+	+	+
11	980	13	3	0	8	2	0	0	+
12	170	5	2	0	3	0	+	+	+
13	364	3	1	0	2	0	-	0	+
14	287	3	0	0	3	0	-	0	0
15	317	7	1	2	4	0	0	-	0
16	174	9	5	0	2	2	+	+	+
17	19	1	0	0	1	0	0	-	+
18	71	4	0	0	4	0	0	-	-
19	34	1	0	0	1	0	+	0	-
20	30	1	1	0	0	0	-	+	-
21	70	1	0	0	1	0	-	+	0
22	181	9	0	0	9	0	-	-	-
23	204	4	1	0	3	0	+	0	+
24	94	3	1	0	2	0	-	-	-
25	72	1	1	0	0	0	+	+	+
26	22	1	1	0	0	0	-	+	-
27	136	7	4	0	3	0	0	+	-
28	373	4	1	1	2	0	-	0	-
29	173	1	1	0	0	0	-	-	0
30	120	7	1	6	0	0	+	+	0
Outlier	160	0	0	0	0	0			
Over all	8009	200	50	49	92	9			

+, -, and 0 indicate up-regulation, down-regulation, and no change, respectively.

Development • Supplementary information

Table S2. Enrichment rate of each gene cluster for Gene list-4.

Cluster No.	Categories in Gene list-4			
	1. cell fate	2. cell division	3 plant hormone	4. small RNA
1	0.8	0.9	0.3	1.9
2	0.0	4.8	0.4	0.0
3	2.0	0.0	2.7	0.0
4	2.2	0.0	4.8	0.0
5	0.3	2.6	0.4	0.6
6	0.6	0.0	2.3	0.0
7	1.3	0.0	2.5	3.6
8	0.0	3.2	0.6	0.0
9	3.5	0.9	1.9	4.9
10	8.0	1.2	0.6	0.0
11	0.5	0.0	0.7	1.8
12	1.9	0.0	1.5	0.0
13	0.4	0.0	0.5	0.0
14	0.0	0.0	0.9	0.0
15	0.5	1.0	1.1	0.0
16	4.6	0.0	1.0	10.2
17	0.0	0.0	4.6	0.0
18	0.0	0.0	4.9	0.0
19	0.0	0.0	2.6	0.0
20	5.3	0.0	0.0	0.0
21	0.0	0.0	1.2	0.0
22	0.0	0.0	4.3	0.0
23	0.8	0.0	1.3	0.0
24	1.7	0.0	1.9	0.0
25	2.2	0.0	0.0	0.0
26	7.3	0.0	0.0	0.0
27	4.7	0.0	1.9	0.0
28	0.4	0.4	0.5	0.0
29	0.9	0.0	0.0	0.0
30	1.3	8.2	0.0	0.0
Outlier	0.0	0.0	0.0	0.0
02+05+23	0.3	2.6	0.5	0.5
10+12+16+25	4.3	0.3	0.9	3.2
8+30	0.6	5.4	0.3	0.0

Table S3. *p* values for enrichment rate of each gene cluster and each category in Gene list-4.

Cluster No.	Categories in Gene list-4			
	1. cell fate	2. cell division	3 plant hormone	4. small RNA
1	0.73	0.71	1.00	0.29
2	1.00	1.5E-03	0.91	1.00
3	0.26	1.00	3.7E-02	1.00
4	0.23	1.00	2.5E-04	1.00
5	1.00	1.6E-06	1.00	0.85
6	0.82	1.00	3.3E-02	1.00
7	0.46	1.00	2.3E-02	0.24
8	1.00	0.06	0.83	1.00
9	2.6E-02	0.67	0.15	0.19
10	2.3E-05	0.58	0.80	1.00
11	0.95	1.00	0.89	0.30
12	0.29	1.00	0.31	1.00
13	0.90	1.00	0.93	1.00
14	1.00	1.00	0.65	1.00
15	0.87	0.58	0.50	1.00
16	4.4E-03	1.00	0.60	1.5E-02
17	1.00	1.00	0.20	1.00
18	1.00	1.00	8.8E-03	1.00
19	1.00	1.00	0.33	1.00
20	0.17	1.00	1.00	1.00
21	1.00	1.00	0.56	1.00
22	1.00	1.00	2.2E-04	1.00
23	0.73	1.00	0.42	1.00
24	0.45	1.00	0.29	1.00
25	0.36	1.00	1.00	1.00
26	0.13	1.00	1.00	1.00
27	1.0E-02	1.00	0.21	1.00
28	0.91	0.90	0.93	1.00
29	0.67	1.00	1.00	1.00
30	0.53	8.2E-05	1.00	1.00
Outlier	1.00	1.00	1.00	1.00
02+05+23	1.00	2.7E-08	1.00	0.91
10+12+16+25	8.0E-07	0.97	0.62	0.13
8+30	0.82	3.2E-05	0.96	1.00

Table S4. *q* value for enrichment rate of each gene cluster and each category in Gene list-4.

Cluster No.	Categories in Gene list-4			
	1. cell fate	2. cell division	3 plant hormone	4. small RNA
1	1.00	1.00	1.00	1.00
2	1.00	1.5E-02	1.00	1.00
3	0.99	1.00	0.19	1.00
4	0.99	1.00	3.8E-03	1.00
5	1.00	4.92E-05	1.00	1.00
6	1.00	1.00	0.19	1.00
7	1.00	1.00	0.18	1.00
8	1.00	0.50	1.00	1.00
9	0.20	1.00	0.68	1.00
10	7.09E-04	1.00	1.00	1.00
11	1.00	1.00	1.00	1.00
12	0.99	1.00	0.84	1.00
13	1.00	1.00	1.00	1.00
14	1.00	1.00	1.00	1.00
15	1.00	1.00	1.00	1.00
16	0.07	1.00	1.00	0.47
17	1.00	1.00	0.71	1.00
18	1.00	1.00	0.09	1.00
19	1.00	1.00	0.84	1.00
20	0.89	1.00	1.00	1.00
21	1.00	1.00	1.00	1.00
22	1.00	1.00	3.8E-03	1.00
23	1.00	1.00	0.99	1.00
24	1.00	1.00	0.84	1.00
25	1.00	1.00	1.00	1.00
26	0.80	1.00	1.00	1.00
27	0.10	1.00	0.71	1.00
28	1.00	1.00	1.00	1.00
29	1.00	1.00	1.00	1.00
30	1.00	1.28E-03	1.00	1.00
Outlier	1.00	1.00	1.00	1.00

Development • Supplementary information

Table S5. Sequences for primers used for genomic PCR.

Gene	Primer name	Sequence
TOP1α	TOP1α 2711 FW	5'-ATCATGAGGCCGACACTGTT-3'
	TOP1α 2948 RE	5'-TGTCCAATGGCCTTGTAAAC-3'
	TOP1α 2520 FW	5'-AAATTGCTGTGGCGACATATC-3'
	TOP1α 3494 RE	5'-ATTGCAGATTATGGCAACCTG-3'
	top1α LB	5'-ACCCCAGTACATTAAAAACGTC-3'
TOP1β	TOP1β +1292 FW	5'-TTCCCAGAGTGTTTTTGCATC-3'
	TOP1β +2409 RE	5'-GGTAAGAAATGGGAAAGCAGG-3'
	TOP1β +2409 FW	5'-CCTGCTTTCCCATTTCTTACC-3'
	TOP1β +3560 RE	5'-CTCCGTGAGATTTCGAGACAG-3'
ETT	ETT LP2	5'-TCTTCTTTTGGCTGAGAGGG-3'
	ETT RP3	5'-GAGATAAGGGTCCAGCACA-3'
pGWB2/OpTOP1	pGWB2 35Sp +5682FW	5'-GACGTTCCAACCACGTCTTC-3'
	OpTOP1 148RE	5’-GTATGCACAGCAGATTGTCC-3’
KRP5	KRP5 -196FW	5'-GATGACAATCAGGTGAGAGG-3'
	KRP5 +73RE	5'-GCTCTGGTACGGAAACCTAGC-3'
	KRP5 +1451FW	5’-CTTCTTTGCTAGCGCAGAAC-3’
	KRP5 +1610RE	5’-TCGTAACGTCCAGGCAATGG-3’
KRP2	KRP2 +202FW	5'-TACGACGGCGAGATTCTCCT-3'
	KRP2 +440RE	5'-CACGACGTTTCTGTTTCACG-3'
SALK LB	LBb1	5'-GCGTGGACCGCTTGCTGCAACT-3'
	LBb1.3	5’-ATTTTGCCGATTTCGGAAC-3’

Development • Supplementary information

Table S6. Combinations of primers for detection of mutation or T-DNA insertion by genomic PCR.

allele or transgenic lines		FW	RE	DNA size (bp)
top1α-1	wild type	TOP1α 2711 FW	TOP1α 2948 RE	258
	mutant	TOP1α 2948 RE	top1α LB	approx.200
mgo1-7 (SALK_112625)	wild type	TOP1α 2520 FW	TOP1α 3494 RE	994
	mutant	TOP1α 2520 FW	LBb1	646
top1β-1 (SALK_069847)	wild type	TOP1β +1292 FW	TOP1β +2409 RE	1138
	mutant	TOP1β +2409 RE	LBb1	600
top1β-2 (SALK_022236)	wild type	TOP1β +2409 FW	TOP1β +3560 RE	1153
	mutant	TOP1β +3560 RE	LBb1	approx.700
ett-13	wild type	ETT LP2	ETT RP3	300
	mutant	ETT LP2	LBb1.3	367
OpTOP1 transgenic plants	T-DNA insertion	pGWB2 35Sp +5682FW	OpTOP1 148RE	448
krp5-1 (SALK_053533)	wild type	KRP5 +1561FW	KRP5 +1610RE	159
	mutant	LBb1.3	KRP5 +1610RE	353
krp5-4 (SALK_089717)	wild type	KRP5 -196 FW	KRP5 +73 RE	269
	mutant	LBb1.3	KRP5 +73 RE	392
krp2-5 (SALK_069817)	wild type	KRP2 +202FW	KRP2 +440RE	238
	mutant	KRP2 +202FW	LBb1.3	393

Development • Supplementary information

Table S7. Sequences for primers used for real-time RT-PCR.

Gene	Primer name	Sequence
BP	BP-F	5'-TGTTGTTTCCACATATGAGCTCTCT-3'
	BP-R	5'-TCATGATCAGATCGGAAGCAAT-3'
KNAT2	KNAT2-F	5'-TTCCGCTCGACGGAAGAC-3'
	KNAT2-R	5'-AATCGGACGGCATCATCAAC-3'
KNAT6	KNAT6-F	5'-GATGTCACCGGAGAGTCTCATG-3'
	KNAT6-R	5'-CGGCGGAGGAACATAGCA-3'
STM	STM-F	5'-CTCCTCCCCAAGGAACTAAGAAC-3'
	STM-R	5'-TCCTCCTGCAACGATTTCG-3'
KAN1	KAN1-F	5'-CCACGCGCGGTTTGTT-3'
	KAN1-R	5'-CGACTTTGGAGTTGCTCTTTCA-3'
KAN2	KAN2-F	5'-AAGGAACTAGATGGAAAGTGCTCAA-3'
	KAN2-R	5'-GCTTGTTCCCGAGATGCTTG-3'
FIL	FIL-F	5'-AAACCAACATGCCCCAACAG-3'
	FIL-R	5'-TCACACCAACGTTAGCAGCTG-3'
YAB5	YAB5-F	5'-ACGCCCTAATTTCCAGGCAAC-3'
	YAB5-R	5'-GTTGCTCAGTTATGGTACGAG-3'
ETT	ETT-F	5'-CGCCTACTCAATAACCGATCATC-3'
	ETT-R	5'-ACGGCCCACACCAAATGTT-3'
ARF4	ARF4-F	5'-CGCTTAAATCATTCCCGCAAT-3'
	ARF4-R	5'-ACTTGTTGGCTTGGTAAGCAAAG-3'
PHB	PHB-F	5'-GCTGTTGACTGGGTTCAGATGA-3'
	PHB-R	5'-GCGAAATAGCGACTATGCCAAT-3'
PHV	PHV-F	5'-GGCGGAGTTCCTTTGCAA-3'
	PHV-R	5'-CCAGGCTTCATCCCAATCAT-3'
ACT2	ACT2-F	5'-TCGGTGGTTCCATTCTTGCT-3'
	ACT2-R	5'-GCTTTTTAAGCCTTTGATCTTGAGAG-3'
EF1α	EF1α-F	5’-TGAGCACGCTCTTCTTGCTTTCA -3’
	EF1α-R	5’-GGTGGTGGCATCCATCTTGTTACA -3’
KRP5	KRP5-F	5'- TCCTAGTGTCAATCAATGTCAAACG-3'
	KRP5-R	5'- CGTCGTATCCGGCTCTAATTT-3'
KRP2	KRP2-F	5'-CGTGGATTTACGATGATTTGAA-3'
	KRP2-R	5'-GCGGCGAGACTCTACATCTT-3'
IPT3	IPT3-F	5'-CCGCCTGAAGCCGACTTAA-3'
	IPT3-R	5'-TTTAGGACGGATTCAATGGAGAGA-3'
KNOLLE	KNOLLE-Q3	5'-TGCAGTCTTCAGCTCATTAGCTC-3'
	KNOLLE-Q5	5'-TGATGGTTGAATCGCAAGGTGAAC-3'
HINKEL	HINKEL_F	5'-CCAAGCAGCGCATCCAA-3'
	HINKEL_R	5'-AAGACTTGCCTAGAAGCTGAAAGC-3'
histone H4	histone H4-Q3	5'-TCCTTGAATGTTATCCCTCAGAAC-3'
	histone H4-Q5	5'-ACAGTCACAGATCTTTGGGTATC-3'
CYCA3;1	CycA3;1-Q3	5'-AGAAGTTTGTATTCTTCAGCAAC-3'
	CycA3;1-Q5	5'-TCTTCGTCAATTAGAGGTAAAGTC-3'
ANAC082	ANAC082-F	5’-CCCCCAACAATGATGACTTATATTC-3’
	ANAC082-R	5’-TCCGTTGAAAATGCCTTCTTCT-3’

Development • Supplementary information
