# Supplementary material for: Arabidopsis epigenetic factor AS2 mitigates nucleolar stress and promotes flat leaf development by repressing a CDK inhibitor
Source: Development. 2026 Sep 18;153(18):dev205275. doi: 10.1242/dev.205275 (PMC13680833; doi:10.1242/dev.205275)
Supplement: Supplementary file 2 [file develop-153-205275_review_history.pdf]

Arabidopsis epigenetic factor AS2 mitigates nucleolar stress and promotes flat leaf development by repressing a CDK inhibitor

Ayami Nakagawa, Hidekazu Iwakawa, Hiro Takahashi, Simon Vial-Pradel, Mari Takahashi, Kazuomi Ohga, Takuma Ito, Motoki Tamai, Eri Takada, Sumie Keta, Nanako Ishibashi, Sayuri Ando, Iwai Ohbayashi, Masaki Ito, Mami Yamazaki, Ayato Sato, Masaaki Umeda, Byung-Yoon Cha, Je-Tae Woo, Michiko Sasabe, Munetaka Sugiyama, Shoko Kojima, Yasunori Machida and Chiyoko Machida

DOI: 10.1242/dev.205275

Editor: Dominique Bergmann

Review timeline

Original submission:	27 September 2025
Editorial decision:	8 November 2025
First revision received:	14 June 2026
Accepted:	24 July 2026

Original submission

First decision letter

MS ID#: dev.205275

MS TITLE: Arabidopsis epigenetic factor AS2 mitigates nucleolar stress and promotes flat leaf development by repressing a CDK inhibitor

AUTHORS: Ayami Nakagawa; Hidekazu Iwakawa; Hiro Takahashi; Simon Vial-Pradel; Mari Takahashi; Kazuomi Ohga; Takuma Ito; Motoki Tamai; Eri Takada; Sumie Keta; Nanako Ishibashi; Sayuri Ando; Iwai Ohbayashi; Masaki Ito; Mami Yamazaki; Ayato Sato; Masaaki Umeda; Byung-Yoon Cha; Je-Tae Woo; Michiko Sasabe; Munetaka Sugiyama; Shoko Kojima; Yasunori Machida; Chiyoko Machida

Dear Dr Machida,

I have now received all the referees' reports on the above manuscript, and have reached a decision. The referees' comments are appended below.

As you will see, the referees express considerable interest in your work, but have some significant criticisms and recommend a substantial revision of your manuscript before we can consider publication. If you are able to revise the manuscript along the lines suggested, which may involve further experiments, I will be happy receive a revised version of the manuscript. Your revised paper will be re-reviewed by one or more of the original referees, and acceptance of your manuscript will depend on your addressing satisfactorily the reviewers' major concerns. Please also note that Development will normally permit only one round of major revision. If it would be helpful, you are welcome to contact us to discuss your revision in greater detail. Please send us a point-by-point response indicating your plans for addressing the referees' comments, and we will look over this and provide further guidance.

Please attend to all of the reviewers' comments and ensure that you upload both a 'clean' version of your Word file, along with a highlighted version clearly showing where you have made changes in the revised manuscript. Please avoid using 'Tracked changes' in Word files as these are lost in PDF

© 2026. Published by The Company of Biologists under the terms of the Creative Commons Attribution License (https://creativecommons.org/licenses/by/4.0/).

conversion. I should be grateful if you would also provide a point-by-point response detailing how you have dealt with the points raised by the reviewers in the 'Response to Reviewers' box. If you do not agree with any of their criticisms or suggestions please explain clearly why this is so.

Reviewer 1

SUMMARY OF THE ADVANCE MADE IN THIS PAPER AND ITS POTENTIAL SIGNIFICANCE TO THE FIELD

Leaf adaxial-abaxial polarity has been studied extensively and there are well-characterised genetic pathways demonstrating repressive interactions between adaxial and abaxial fate. One of the genes involved in this pathway is AS2. Several studies, including those of the authors, have demonstrated AS2 acts in a complex associated with the nucleolus and AS2 is postulated to regulate transcription of genes such as rRNA. How this relates to leaf adaxial-abaxial patterning and cell fate is not known. The experiments in the current manuscript nicely combine physiological and genetic approaches to extend our understanding of AS2 function. The manuscript describes screening a library of chemical compounds that induce radial leaves in the as2 mutant identifies several compounds, one of which is a known inhibitor of topoisomerase I and nucleolar integrity. It is shown that this chemical leads to increased levels of abaxial gene ETT transcripts and genetically it is shown AS2 and TOP1a suppress expression of the CDK cell cycle inhibitor KRP5. Testing other chemicals demonstrated radial leaves in as2 of TOPII inhibitors but not chemicals that interfere with microtubules and cell cycle M phase progression. The results from selective chemical treatments that induce radial leaves in as2 mutants is qualified through genetic analysis. Combining mutants in TOP1a with as2 increases radial leaf production, and this is suppressed by mutations in the abaxial gene ETT and by KRP5. Although a synthesis of AS2 function in relation to nucleolar integrity and cell cycle progression remains to be fully determined the data in this manuscript provide further interesting and intriguing insights into this pathway of leaf patterning.

Comments for the author

Major comments:

Line 153: the chemical library is screened at a concentration of 12.5 μM. Please provide a rational for selecting this concentration.

Line 205: ETTp:GUS is expressed in radial leaves of 10H-CPT as2. No conclusion is drawn from this data. Does the reporter expression pattern change in 10H-CPT treated as2 radial leaves relative to as2? These leaves should be sectioned to show the pattern of expression.

Line 207-209: explain the rational for examining KNOXI expression and loss-of-function effects on the radial leaves of chemically treated as2.

Line 220: Have the authors confirmed the insertion site for the T-DNA in the top1ß mutants? Are these null alleles? Likewise is krp2-5 a null allele? It might effect the outcome if these are weak alelles.

Line 222: why is there a difference in the severity of effect of top1 and mgo alelles on as2 and as1?

Line 279: There is reference to cell fate genes such as BP and ETT being enriched in as1 relative to wt. But Figure S3C shows no change in cluster 12 BP expression in wt + 10H-CPT vs as2 with and without the chemical inhibitor. This requires clarification.

Line 274: more detail is required on the data clustering. Unless I have missed something the references provided describe around 14 clusters, whereas this manuscript describes 30 clusters. It is unclear what the clusters are.

Line 282: To narrow down candidate target genes a comparison has been carried out with expression data from BOB1 and ELO3. It would be useful to have an explanation of the rational for selecting this data set and these particular modifiers of as2 and as1.

© 2026. Published by The Company of Biologists under the terms of the Creative Commons Attribution License (https://creativecommons.org/licenses/by/4.0/).

Line 325- 330: KRP5/ICK3 expression peaks during S phase in synchronised cells. The expression pattern is described as "broad" during M phase and overlapping with peaks of M-phase specific genes. How does this broad expression pattern relate to the suggestion that KRP5/ICK3 acts as a repressor of progression in the M phase of the cell cycle? I think the data are overinterpreted.

Line 335: CYCB1;2p:GUS expression levels are compared in wt, as2, top1α and the double mutant. How were these lines generated? How many independent lines were examined? Could the low level of expression be due to transgene insertion site?

Line 350: state the rational for the additional chemical treatments. Were these chemicals selected to test for redundancy between topI and topII?

Line 374: Transcripts of ETT and KRP5 did not increase in propyzamide-treated as2 plants. However, this was tested at a concentration (5uM) that does not induce a radial-leaf phenotype (Fig. 5). Why use 5uM for this analysis? Did the authors assay the transcript levels for these genes in plants grown at higher propyzamide concentrations?

Line 461: I think this paragraph in the discussion could be used to link with the data on ETT.

Minor comments

Line 67-70: the sentence is unclear, requires rephrasing.

Line 72: "division of cells locating along.." suggest change to "division of cells located along.."

Line 74: "polarity determinants should play a role.." suggest remove the word "should".

Line 98: "is tightly related to the repression level of ETT/ARF3 transcript .." might be better phrased "is tightly related to the repression of ETT/ARF3 transcript levels"

Line 162-165: the sentence is awkward, needs rephrasing.

Line 199-200: What is meant by "peripheral positions" regarding the radial leaves of as2 and as1 treated with 10H-CPT? I think this requires a clearer description.

Line 202: adaxial should be abaxial.

Line 354: "allows the passage of a double-stranded DNA segment .." is unclear and requires clarification.

Line 489: The term "guardian angel" is defined as "An angel conceived as watching over or protecting a particular person or place" and "angel" is defined as "…a member of a class of celestial beings considered intermediate between God and humanity" (OED). I don't see how the description of a protein or gene can be congruent with these definitions. I suggest using different terminology.

Table 2: G418 analog has a comment "N/A" but the text notes this chemical is an inhibitor of protein synthesis.

Figure 1C (and other graphs): The X and Y axes should be fully labelled. For Fig1C should be something like 10H-CPT concentration (uM) on the X axis and "leaf shape frequency" on the Y axis.

Figure 2A and other similar figures: the figure legend should state what the % indicates (is this the frequency of plants without radial leaves?) and what is in the brackets (is this the number of plants without radial leaves over the total number of plants examined?).

Figure 3E: were the plants used for this qRT-PCR treated with 10H-CPT? Include the information in the figure.

The title is somewhat unwieldy. Possibly "Arabidopsis epigenetic factor AS2 attenuates nucleolar stress to establish leaf polarity by repressing a CDK inhibitor".

© 2026. Published by The Company of Biologists under the terms of the Creative Commons Attribution License (https://creativecommons.org/licenses/by/4.0/).

Reviewer 2

The AS2 gene plays an important role in determining the upper (adaxial) epidermis. A chemical compound screen identified topoisomerase inhibitors (CPT and related compounds) as agents that inhibit adaxialisation in an as2 mutant background (this was confirmed using abaxial reporter lines). These observations were subsequently confirmed genetically using available topoisomerase mutants, which demonstrated that this was caused by increased transcript levels of the ETT/ARF3 gene. Most interestingly, through transcriptome analysis, the cyclin-dependent kinase gene KRP5 was identified as a downstream target, a finding that was supported by genetic data.

Overall, this manuscript is rich in data and makes a robust and novel contribution to our understanding of leaf polarity regulation in Arabidopsis thaliana, presenting novel insights into the role of AS2 in regulating leaf polarity via CDK inhibition. The work logically progresses from chemical screening to genetic interactions.

Major comments:

1. While I find the macroscopic and RT-PCR data on loss of abaxialisation convincing, the role of cell division in the process is currently overinterpreted. Currently, the authors rely on CYCB1;2-GUS reporter activity in the presence or absence of topoisomerase activity. A better approach would be to make a comparison in the as2 krp5 top1 (or as2 krp5) background. As introducing this marker line into these mutant backgrounds might take a long time, an alternative would be to use leaf clearing to estimate the number of cells in both epidermal tissues.

2. A related shortcoming is the absence of data on a KRP5 reporter line, despite such a line being available in the field (see, for example, the cited Echevarría et al. paper recently published in Nature Plants). Does it demonstrate any abaxial-adaxial specificity? Alternatively, such data could be extracted from recent leaf scRNA-seq experiments that have been published.

3. Another shortcoming is that the authors ignored the possible effects of endoploidy, a process known to be controlled by KRP genes. It should also be noted that many of the mutants previously found to act together with AS2 in determining leaf adaxialisation possess a ploidy link (e.g. ribosome and nucleosome components).

Secondary comments and questions:

1. Some sentences in the introduction are long and complex, making them hard to follow, e.g. lines 67-70 and 100-104.

2. Define 'nucleolar stress' in the introduction rather than the discussion section.

3. Section 363-369: (related to Figure 5): might a dose of propyzamide that is too high have been used to conclude that impaired M phase progression does not trigger a similar phenotype? Given the bleached leaves (compared to the etoposide plants), it appears that these plants are dying. A similar comment applies to the RT-PCR data, for which the figure legend does not specify the length of treatment. Overall, the legends could provide more explanation to enable standalone understanding. Overall, the rationale for choosing certain concentrations or compounds could be better justified.

4. Is the nucleolar bodies phenotype still present in the top1 as2 sriw1 mutant?

Reviewer 3

SUMMARY OF THE ADVANCE MADE IN THIS PAPER AND ITS POTENTIAL SIGNIFICANCE TO THE FIELD

Nakagawa et al. described the involvement of TOPI function in regulating leaf morphology, through the interaction with AS2, an epigenetic regulator. Multiple compounds with the effect of enhancing

© 2026. Published by The Company of Biologists under the terms of the Creative Commons Attribution License (https://creativecommons.org/licenses/by/4.0/).

the as2 phenotype were isolated from chemical screening, and among them, the authors focused on the mode of action of camptothecin, a well-known topoisomerase I inhibitor. Indeed, the top1α mutation enhanced as2 phenotype to induce filamentous leaves and seedling lethality. The authors also found that ETT/ARF3 and/or KRP5/ICK3 genes were upregulated in as2, top1α, and as2 top1α mutants, and that the mutations of them can partially suppress the phenotypes of as2 top1α as well as the camptothecin effects, indicating that the severe leaf morphology and lethal phenotype in as2 top1α would be attributable to ETT/ARF3 and KRP5/ICK3. They also demonstrated that the induction of genomic stress, such as DNA double-strand breaks, rather than cell cycle progression inhibition, enhanced the upregulation of ETT/ARF3 and KRP5/ICK3 expression and the formation of filamentous leaves in as1 or as2 mutants. Finally, the top1α mutation and camptothecin disturbed the number and morphology of AS2 bodies. Taken together with the suppression effect of sriw1 mutation, in which the regulator of nucleolus stress response is defective, the authors concluded that AS2 would function as a mediator of TOPI-related nucleolus stress response to the determination of leaf morphology. Overall, the study was well-executed and undoubtedly contributes to a deeper understanding of the effects of nuclear (or nucleolar) stress on leaf morphogenesis.

SUGGESTIONS TO AUTHORS

While I believe important findings are presented in the manuscript, to support the conclusion, several questions remain. The major points are as follows:

1. Title and discussions: The leaf morphogenesis process related to TOPI and KRP5 may not be the stage of polarity determination itself, but rather the stage of cell division control that occurs after polarity determination. Or, do the authors think that the control of cell division itself is included in leaf polarity determination? (Based on the last sentence of the first paragraph in Introduction, I understood that these stages are considered separately, but is that incorrect?) Throughout the paper's title and discussions, the terms like leaf morphogenesis and polarity determination are used interchangeably sometimes. However, to place the AS2-ETT-KRP5 pathway precisely on the leaf development, I would like the authors to clarify this once more. In other words, the current title is highly misleading. I request a careful reconsideration, including the phrasing used throughout the discussion.

2. The experimental verification of the involvement of TOPI and KRP5 in leaf morphology is an excellent result. However, the view that "ETT is a target of AS2, and KRP5 would act as its downstream regulator" and that "nucleolar stress is involved in as2 leaf morphogenesis" have already been reported by the authors, i.e. these points are not novel. To ensure the novelty of this paper, it is necessary to examine how the top1α mutant or camptothecin affect nucleolar structure using markers other than AS2-YFP. Furthermore, it is necessary to clarify whether ETT-mediated induction of KRP5 expression occurs through the direct transcriptional activation by ETT.

3. The authors state that AS2 and TOP1 "synergistically" regulate cell proliferation (e.g. line 308, 319, 344). But where is the "synergy"? Since ETT expression levels are approximately twice wild-type in as2 and top1α single mutants, and 4-5 times in double mutants, this is simply additive, not synergistic, I guess. Furthermore, it is problematic that KRP5 expression in top1α single mutants has not been examined. Please investigate the KRP5 expression in top1α.
Even in the final model presented in Fig. 7, TOP1 is simply located at the upstream of AS2. If the effect is synergistic (or even additive), this contradicts the authors' claim. This point also requires either a revision of the description or a modification of the model.

4. The descriptions related to Figure 3, S3, and the corresponding supplemental Tables need to be rewritten. Materials and Methods section completely lacks any explanation of the criteria used for gene expression analysis and clustering analysis. Without this information, the readers cannot understand what kind of gene analysis was performed. Specifically, the gene categorization such as "cell fate" and "cell division was serious problem. How were these categories determined? If GO term enrichment was used, this should be explicitly stated. If an in-house method was employed, it should be disclosed carefully. Particularly since the microarray experiments are now irreproducible, more careful description is essential.

© 2026. Published by The Company of Biologists under the terms of the Creative Commons Attribution License (https://creativecommons.org/licenses/by/4.0/).

5. Please examine the ETT and KRP5 expression level in as2 top1α sriw1 triple mutant, to make sure the nucleolus stress response-based leaf phenotype is mediated by the ETT and KRP5 upregulation.

6. Figure 5 and S6: please check the effects of these chemicals on as2 ett and as2 krp5, or more appropriate mutant lines, to conclude "the genome stress affects AS2-ETT-KPR5 pathway," as the authors claimed. In addition,the top2 mutant analysis might be required. However, to be honest, I feel this section is unnecessary for this paper. Although the authors would like to conclude with nucleolar stress, this part discussed the genomic stress (and TopII) instead, leaving it hanging and not incorporated into the model in Fig. 7. The data showing oryzalin nor propyzamide did not affect leaf morphogenesis is very interesting, as it indicates the importance of something beyond simple cell divisional control, therefore it would be reasonable to leave as is.

Minors

1. For the order of data appearance: Fig. S1A and S1B should be re-named, since Fig. S1B is appeared first.

2. Page 9 Line 282 and 294, what means "common"? Please make sure common between what and what.

3. Line 381: "Cell biology with the recombinant…" should be "Cell biological analysis with the recombinant…". "Cell biology" is too huge to describe your own analysis with AS2, I feel.

4. Line 489 and other places: "AS2 is a guardian angel" this expression is not acceptable for me. This is far too abstract and religious for a scientific paper. Especially since the as2 ko mutant is viable, thus AS2 is not an essential gene. In this sense, the term "guardian angel" is highly misleading. Please reconsider your phrasing.

5. Related to above comment: the final section of Discussion part (line 489-557) contains many repetitions of results and introductions to past findings, and I believe it lacks substantive messages relevant to the results of this paper. Therefore, it should be made much shorter.

6. Line 288-289 "10 genes encoding proteins with known domains such as ETT/ARF3 an BP", I could not understand the meaning of this phrase. Please reword for easy understanding.

7. Figure legends for Fig 1D, 2A,C,D, Fig. 6 (and so on): The author must clearly state what the percentage represents.

First revision

<u>Author response to reviewers' comments</u>

Reviewer 1:

SUMMARY OF THE ADVANCE MADE IN THIS PAPER AND ITS POTENTIAL SIGNIFICANCE TO THE FIELD

Leaf adaxial-abaxial polarity has been studied extensively and there are well- characterised genetic pathways demonstrating repressive interactions between adaxial and abaxial fate. One of the genes involved in this pathway is AS2. Several studies, including those of the authors, have demonstrated AS2 acts in a complex associated with the nucleolus and AS2 is postulated to regulate transcription of genes such as rRNA. How this relates to leaf adaxial-abaxial patterning and cell fate is not known. The experiments in the current manuscript nicely combine physiological and genetic approaches to extend our understanding of AS2 function. The manuscript describes screening a library of chemical compounds that induce radial leaves in the as2 mutant identifies several compounds, one of which is a known inhibitor of topoisomerase I and nucleolar integrity. It is shown

© 2026. Published by The Company of Biologists under the terms of the Creative Commons Attribution License (https://creativecommons.org/licenses/by/4.0/).

that this chemical leads to increased levels of abaxial gene ETT transcripts and genetically it is shown AS2 and TOP1a suppress expression of the CDK cell cycle inhibitor KRP5. Testing other chemicals demonstrated radial leaves in as2 of TOPII inhibitors but not chemicals that interfere with microtubules and cell cycle M phase progression. The results from selective chemical treatments that induce radial leaves in as2 mutants is qualified through genetic analysis. Combining mutants in TOP1a with as2 increases radial leaf production, and this is suppressed by mutations in the abaxial gene ETT and by KRP5. Although a synthesis of AS2 function in relation to nucleolar integrity and cell cycle progression remains to be fully determined the data in this manuscript provide further interesting and intriguing insights into this pathway of leaf patterning.

<Response>: We wish to express our appreciation to the Reviewer 1 for his or her insightful comments, which have helped us significantly improve the paper.

Major comments:

Line 153: the chemical library is screened at a concentration of 12.5 μM. Please provide a rational for selecting this concentration.

<Response>: We determined concentrations for selecting based on the following our papers. As shown in Fig. 1A,B, we used concentration of 12.5 μM of each molecule (2209 molecules) for first screening and selected molecules inhibited growth. For second screening, we used several concentrations of each molecule.

[Line 143-144] We added the following words and references.
"Based on previous studies(Nakagawa et al., 2012; Iwasaki et al., 2013; Luong et al., 2018), "
Nakagawa et al. Plant Mol. Biol. 2012
https://link.springer.com/article/10.1007/s11103-012-9929-7
Iwasaki et al. Development 2013
https://journals.biologists.com/dev/article/140/9/1958/46136
Luong et al., Plant Biotechnology 2018
https://pubmed.ncbi.nlm.nih.gov/31275036/

Line 205: ETTp:GUS is expressed in radial leaves of 10H-CPT as2. No conclusion is drawn from this data. Does the reporter expression pattern change in 10H- CPT treated as2 radial leaves relative to as2? These leaves should be sectioned to show the pattern of expression.

<Response>:
The signals due to *FILp-GFP* were detected on either surface of the filamentous leaves of the *as2-1* +10H-CPT. Furthermore, *ETT/ARF3* expression was observed in rod- shaped leaves, but *BPp:GUS* expression was not observed in rod-shaped leaves (Fig. S1), suggesting that rod-shaped filamentous leaves are abaxialized. However, as Reviewer 1 pointed out, these data do not suggest that the filamentous leaves caused in *as2-1* plants treated with 10H-CPT are due to increased *ETT/ARF3* expression. Therefore, we changed the sentence as follows.
[Line 202-203] "These results suggested that the filamentous leaves caused in *as2-1* plants treated with 10H-CPT are abaxialized."

GUS reporter expression changed in 10H-CPT treated *as2* radial leaves. As shown in the revised **Fig. 1G**, we showed the GUS reporter expression in *as2* leaves.

Fig. 1G

© 2026. Published by The Company of Biologists under the terms of the Creative Commons Attribution License (https://creativecommons.org/licenses/by/4.0/).

Fig. 1G

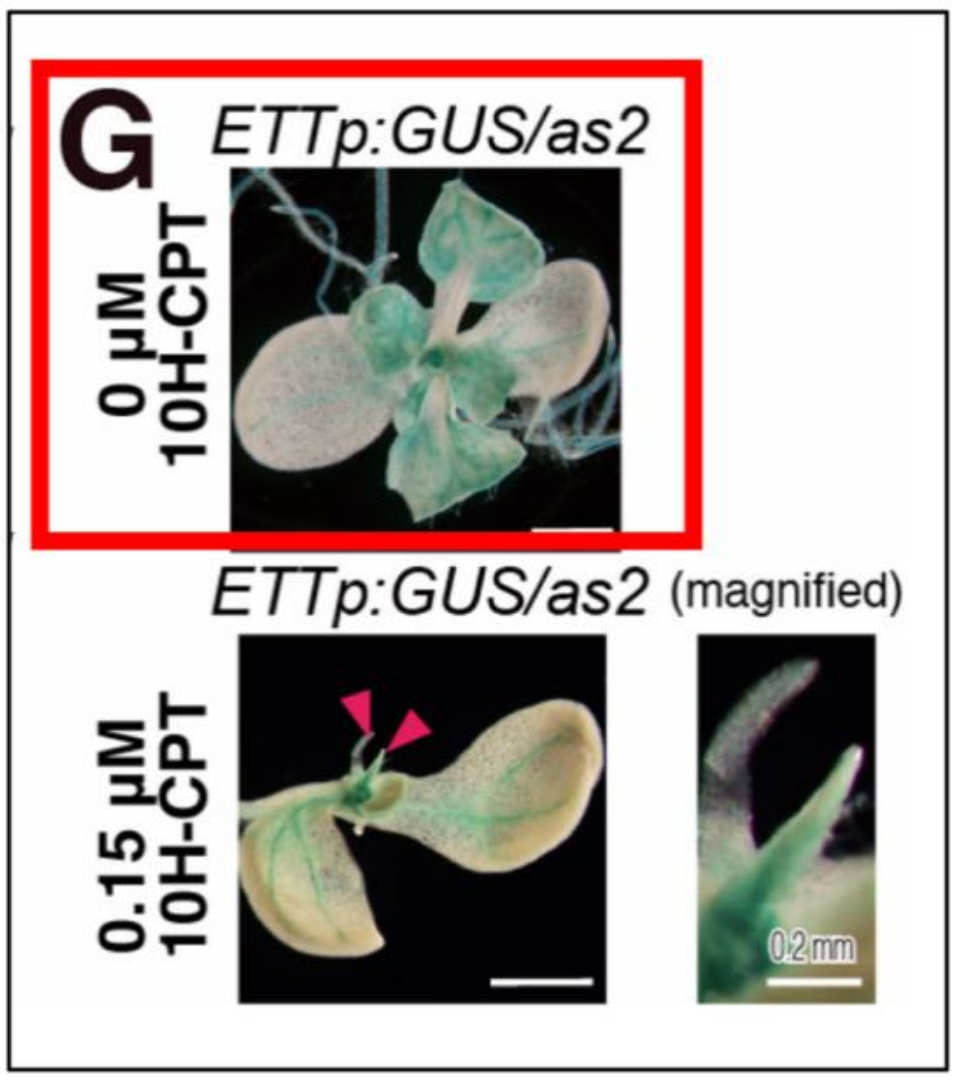

Reviewer 1 commented that these leaves should be sectioned to show the pattern of expression. As reviewer 1 pointed, the characterization of the filamentous leaves would have been more clearly revealed if sections had been prepared. We apologize for not being able to section the filamentous leaves. Since none of the *ETTp:GUS* transgenic seeds germinated, we think it will be necessary to regenerate the *ETTp:GUS* transgenic plants.

Line 207-209: explain the rational for examining KNOXI expression and loss-of- function effects on the radial leaves of chemically treated as2.

<Response>: To confirm the properties of filamentous leaves, we analyzed *BPp:GUS* activity. AS2 and AS1 directly repress *KNOXI* (*BP*, *KNAT2*, *KNAT6*) expression and *ETT*/*ARF3* expression. If class 1 *KNOX* (*BP*, *KNAT2*, *KNAT6*) expression were detected in filamentous leaves, these leaves would be considered to have meristem-like properties. Results showed that *BPp:GUS* activity was not detected in filamentous leaves, but *ETTp:GUS* expression was detected, indicating that filamentous leaves have abaxial properties.

Line 220: Have the authors confirmed the insertion site for the T-DNA in the top1ß mutants? Are these null alleles? Likewise is krp2-5 a null allele? It might effect the outcome if these are weak alelles.

<Response>: Yes, we confirmed the insertion site for the T-DNA in the *top1ß* mutants. The *top1ß-1* mutant (SALK_069847) has been described in Zhang et al., 2016. The *top1ß-1* mutant is null allele.
Zhang et al., *Plant Physiology* (2016) 171, 483-493
https://doi.org/10.1104/pp.15.01754
We confirmed the insertion site for the T-DNA in the *krp2-5* mutant.
The *krp2-5* (SALK_069817) mutant has been described as null allele in Vieira et al., 2013.
Vieira et al., New Phytologist (2013) 199: 505–519
https://doi: 10.1111/nph.12255
We cited these papers as followes

[Line 507-508] In this study, the T-DNA insertion lines SALK_069847, SALK_022236, SALK_089717, and SALK_069817 are designated as *top1ß-1* (Zhang et al., 2016), *top1ß-2*, *krp5-4*, and *krp2-5* (Vieira et al., 2013), respectively.

Line 222: why is there a difference in the severity of effect of top1 and mgo alelles on as2

© 2026. Published by The Company of Biologists under the terms of the Creative Commons Attribution License (https://creativecommons.org/licenses/by/4.0/).

and as1?

<Response>: The *top1a-1* mutant seeds were kindly provided from Dr. Taku Tkahashi. He published characterization of *top1a-1* mutant in Takahashi et al., Plant Cell 2002. The *top1a-1* is a single recessive mutant.
The *mgo1-7* mutant (SALK_112625) was obtained from ABRC. We crossed the *mgo1-7* mutant with *as1-1* or *as2-1* generating *as1-1 mgo1-7* or *as2-1 mgo1-7* doble mutants. Therefore, it is possible that the *mgo1-7* mutant line (SALK_112625) has still second mutation. Such a second mutation may affect phenotype of these double mutants. On the other hand, we cannot rule out a possibility that differences in mutation sites may have had an influence.

Line 279: There is reference to cell fate genes such as BP and ETT being enriched in as1 relative to wt. But Figure S3C shows no change in cluster 12 BP expression in wt + 10H-CPT vs as2 with and without the chemical inhibitor. This requires clarification.

<Response>: We apologize that we did not explain the criteria used for gene expression analysis and clustering analysis in Materials and Methods section.
Among these data, the log2 FC values that were > 1 or < -1 were rounded to 1 and -1, respectively; the log2 FC values with no change were rounded to 0, to avoid category proliferation in clustering.

[Line 680-682] We described this in **Microarray data processing in MATERIALS and METHODS**

Among these data, the log2 FC values that were > 1 or < -1 were rounded to 1 and -1, respectively; the log2 FC values with no change were rounded to 0, to avoid category proliferation in clustering.

Line 274: more detail is required on the data clustering. Unless I have missed something the references provided describe around 14 clusters, whereas this manuscript describes 30 clusters. It is unclear what the clusters are.

<Response>: We apologize that we did not explain KB-FuzzyART in Materials and Methods section. 30 clusters are correct.
We added **Microarray data processing** in **MATERIALS AND METHODS** (as shown in **[Line 666-699]**)

KB-FuzzyART is a knowledge-based clustering method (algorithm) developed in the field of bioinformatics research. Its purpose is to improve the efficiency and accuracy of clustering (grouping) by utilizing existing biological knowledge (such as known genes) when analyzing complex datasets such as gene expression data.
Its main features are as follows: • Use of knowledge: By incorporating known gene information before clustering all the data, the accuracy of the analysis can be improved.
• Applications: It has been applied to research on genes involved in leaf development and differentiation in Arabidopsis thaliana, and to the analysis of gene clusters related to diseases such as cancer. • FuzzyART-based: It is based on a machine learning algorithm called FuzzyART, and can flexibly handle similarities between data. This method is useful for discovering new regulatory networks and unknown candidate genes from large amounts of genetic data.

Line 282: To narrow down candidate target genes a comparison has been carried out with expression data from BOB1 and ELO3. It would be useful to have an explanation of the rational for selecting this data set and these particular modifiers

<Response>:
We apologize that we did not have an explanation of the rational for selecting this data set and these particular modifiers, *BOB1* and *ELO3*.

© 2026. Published by The Company of Biologists under the terms of the Creative Commons Attribution License (https://creativecommons.org/licenses/by/4.0/).

We added the following sentences.

[Line 268-286] As we previously reported that mutations of *BOBBER1* (*BOB1*)/*ENHANCER of AS2 and AS1* (*EAL*) and *ELONGATA3* (*ELO3*)/*ENHANCER of ASYMMETRIC LEAVES TWO1*(*EAST1*) act as modifiers of as2 and as1 to enhance both the adaxial defect to generate filamentous leaves with an abaxialized surface, and the expression levels of many genes including *ETT/ARF3* increased in *as2 bob1/eal* and *as2 elo3* double mutants (Kojima et al. 2011, Ishibashi et al. 2012). *BOB1/EAL* is a gene that encodes an Arabidopsis ortholog of eukaryotic NudC domain proteins (Jurkuta et al. 2009, Perez et al. 2009; Ishibashi et al., 2011). *ELO3/EAST1* is a gene that encodes the histone acetyltransferase, a component of the Elongator complex (Wittschieben et al., 1999; Pandey et al., 2002; Kojima et al., 2011).

We have previously carried out clustering analysis by KB-FuzzyART (Takahashi et al., 2013; Iwasaki et al.,2013), using microarray data of *as2 bob1/eal* and *as2 elo3* double mutants (Kojima et al. 2011, Ishibashi et al. 2012).

We extracted 57 overlapping genes co-regulated by both *BOB1/EAL* and *ELO3/EAST1* (Fig. 3A; Kojima et al., 2011; Ishibashi et al., 2012; Takahashi et al., 2013). We further identified downstream factors by merging between 57 candidates regulated by *AS2*, *AS1*, *ELO3* and *EAL* (Takahashi et al., 2013) and 556 genes in clusters 10, 12, 16, and 25 extracted in this experiment (Fig. S3). Results showed that 18 genes are overlapped (Fig. 3A).

Line 325- 330: KRP5/ICK3 expression peaks during S phase in synchronised cells. The expression pattern is described as "broad" during M phase and overlapping with peaks of M-phase specific genes. How does this broad expression pattern relate to the suggestion that KRP5/ICK3 acts as a repressor of progression in the M phase of the cell cycle? I think the data are overinterpreted.

<Response>: We appreciate the Reviewer’s helpful comment.
We also think the following sentence (original 330-331) was "the data are overinterpreted." Therefore, we simply rewrote it as follows.

[original Line 330-331] These findings were consistent with the notion that *KRP5/ICK3* might act as a factor in a repressive manner on the progressions of S and M phases of the cell cycle.
[Line 340-341] These results suggest that *KRP5/ICK3* expression is cell cycle- dependent and associated with cell cycle progression.

In this paper, we show that AS2 regulates *KRP5/ICK3* expression via *ETT/ARF3*. The factors directly regulating *KRP5/ICK3* expression during leaf development remain unknown. Furthermore, no clear binding of KRP5/ICK3 to either CDKA or CDKB has been shown (Nakai et al., FEBS Letter 2006). The molecular function of *KRP5/ICK3* remains unknown.

Line 335: CYCB1;2p:GUS expression levels are compared in wt, as2, top1α and the double mutant. How were these lines generated? How many independent lines were examined? Could the low level of expression be due to transgene insertion site?

<Response>: We used a *CYCB1;2p:GU*S transgenic line established in a wild-type background and crossed it with *as2* and *top1α* to introduce *CYCB1;2p:G*US into each mutant background. In this case, we are observing the expression of a transgene introduced into the same locus, so we believe this alleviates the reviewer's concerns. We added a sentence in **MATERIALS and METHODS** as follows.

[Line 580-581] We crossed a *CYCB1;2p:GUS*/Col-0 transgenic line with *as2-1*, *top1α- 1*, and *krp5-1*.

Line 350: state the rational for the additional chemical treatments. Were these chemicals selected to test for redundancy between topI and topII?

<Response>: We apologize for not clearly stating the purpose of the experiments involving the addition of TOPII inhibitors such as etoposide. The reason for the additional chemical

© 2026. Published by The Company of Biologists under the terms of the Creative Commons Attribution License (https://creativecommons.org/licenses/by/4.0/).

treatments, rather than testing for redundancy, was that the TOPII inhibitor etoposide has also been shown to induce nucleolar stress in animal cells.
We added sentences as follows.

[Line 360-370)] As shown in Fig. 5A,C and Fig. S9A,B, we further examined the effects of the following reagents on leaf development in the *as2-1* and *as1-1* backgrounds: etoposide, a specific inhibitor of type II DNA topoisomerase (TOPII);mitomycin C, an inhibitor of DNA synthesis that introduces cross-links into DNA at purine bases; and bleomycin A5, which induces double-strand breaks (Kampranis et al., 1999; Yogo et al., 2012; Hutten et al., 2011; Burger et al., 2010; Boulon et al., 2010; González-Arzola, 2024). Etoposide, mitomycin C, and bleomycin A5 have also been shown to induce nucleolar stress in animal cells (Hutten et al., 2011; González-Arzola, 2024). The *as2-1* and *as1-1* mutant plants that were grown in the presence of these molecules formed filamentous leaves, although Col-0 plants scarcely formed, as observed upon the treatment with 10H-CPT (Fig. 5A,C, Fig. S9A,B).

Line 374: Transcripts of ETT and KRP5 did not increase in propyzamide-treated as2 plants. However, this was tested at a concentration (5uM) that does not induce a radial-leaf phenotype (Fig. 5). Why use 5uM for this analysis? Did the authors assay the transcript levels for these genes in plants grown at higher propyzamide concentrations?

Response: At higher concentrations of propyzamide, most plants died, so we were unable to measure the transcript levels of these genes. Therefore, we measured them at 5 μM in intact plants. We also conducted experiments at concentrations lower than 5 μM. We found that rod shape of leaves was not generated in either case.

We show propyzamide all-dose- phenotype.

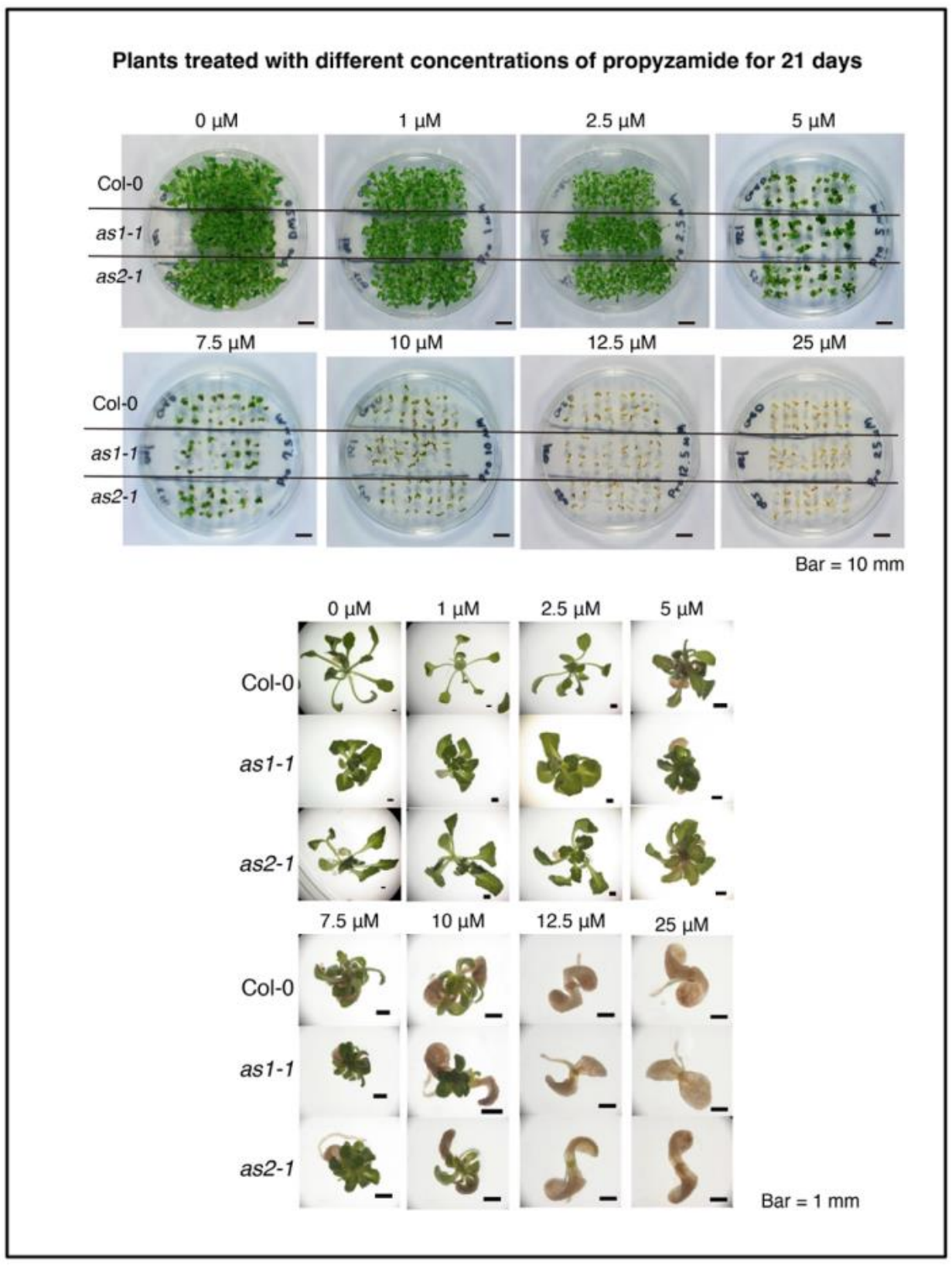

Line 461: I think this paragraph in the discussion could be used to link with the data on ETT.

<Response>: We appreciate the reviewer 1 for suggestion that this paragraph in the discussion

© 2026. Published by The Company of Biologists under the terms of the Creative Commons Attribution License (https://creativecommons.org/licenses/by/4.0/).

could be used to link with the data on *ETT/ARF3*.
It is thought that AS2-AS1 repression of *ETT/ARF3* expression leads to differentiation of superficial cells, resulting in lateral cell division. Although *KRP5/ICK3*has been shown to be downstream of *ETT/ARF3* (Fig. 4C), ETT/ARF3 does not appear to directly regulate *KRP5/ICK3*. Simonini et al. (2017) performed ChIP-seq experiments to identify genes potentially directly regulated by *ETT/ARF3*. However, *KRP5/ICK3*was not among the candidate genes (Simonini et al. 2017). Future work will be important to identify factors that directly regulate *KRP5/ICK3*, analyze the expression pattern of *KRP5/ICK3*, and elucidate its molecular function.

We added the following sentence to link with the data on *ETT/ARF3*.

[Line 450-451] AS2 bodies may function in maintaining the repressed state of the *ETT/ARF3* gene.

Minor comments

Line 67-70: the sentence is unclear, requires rephrasing.
Original :The initial leaf primordium is radial with abaxial cells, and some parts of the basal cells more adjacent to the top of the radially shaped SAM could initiate to differentiate into adaxially fated cells, establishing the leaf adaxial- abaxial polarity (Byrne et al., 2000; Iwakawa et al., 2002; Bowman 2002; Newman et al., 2002; Tsukaya 2006; Fukushima & Hasebe 2014).

<Response>: We appreciate the comment by the reviewer 1. We hope the following sentences become clear.

Revised:

[Line 68-72] During early leaf development, adaxial fate is established in cells adjacent to the SAM, thereby generating adaxial–abaxial polarity, and subsequent cell proliferation along the medial–lateral axis gives rise to a flat leaf blade (Byrne et al., 2000; Iwakawa et al., 2002; Bowman, 2002; Newman et al., 2002; Tsukaya, 2006; Fukushima and Hasebe, 2014).

Line 72: "division of cells locating along." suggest change to "division of cells located along. " OK”

Line 74: "polarity determinants should play a role." suggest remove the word "should".

<Response>: We appreciate the comments（Lines 72 and 74）by the reviewer 1.

Because the sentences were unclear and redundant, line 72-78 (Original) have been shortened as follows:

[Line 72-75] Although antagonistic interactions between adaxial and abaxial determinants are essential for leaf flattening, the mechanisms by which these factors regulate cell division remain unclear (Wu et al., 2008; Iwasaki et al., 2013; Fukushima and Hasebe, 2014; Machida et al., 2015; Husbands et al., 2015).

Line 162-165: the sentence is awkward, needs rephrasing.
Original sentence: Lovastatin analogs, the primary target of which is the inhibition of HMG-CoA reductase (HMGR), the rate-limiting enzyme in cholesterol biosynthesis, are drugs used to treat dyslipidemia, and are molecules that suppress the synthesis of ATP, an essential energy source for cell survival.

<Response>: We appreciate the comment by the reviewer 1.
We rephrased the sentence. We hope the following sentences become better. **Revised sentence:**
[Line 154-157 and Table 1] Lovastatin analogs are therapeutic agents for dyslipidemia that act by inhibiting HMG-CoA reductase (HMGR), the rate- limiting enzyme in cholesterol biosynthesis. In addition, these molecules also reduce ATP production, a critical energy source required for cell survival.

© 2026. Published by The Company of Biologists under the terms of the Creative Commons Attribution License (https://creativecommons.org/licenses/by/4.0/).

Table 1

lovastatin analog	possible inhibitor of HMG-CoA reductase	2650832-70-3	(1S,3R,7S,8S,8aR)-8-{2-[(2R,4R)-4-hydroxy-6-oxooxan-2-yl]ethyl}-3,7,8a-trimethyl-1,2,3,7,8,8a-hexahydronaphthalen-1-yl (2S)-2-methylbutanoate	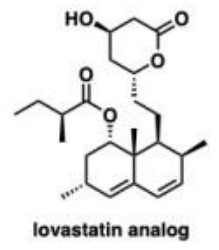 lovastatin analog

Line 199-200: What is meant by "peripheral positions" regarding the radial leaves of as2 and as1 treated with 10H-CPT? I think this requires a clearer description.

<Response>: We appreciate the comment by the reviewer 1. We changed the sentence. We hope the following sentence become a clearer description.

[Line 190-192] As shown in Fig. 1F, strong signals of the GFP fluorescence were detected in cells of the surface of the filamentous leaves induced by the treatment of *as2-1* and *as1-1* plants with 10H-CPT

Line 202: adaxial should be abaxial.

<Response>:
[Line 194] We corrected “adaxial “to “abaxial”.

Line 354: "allows the passage of a double-stranded DNA segment." is unclear and requires clarification.
Original sentence Line 351-355: etoposide, a specific inhibitor of type II DNA topoisomerase (TOPII), which induces a double-stranded break, allows the passage of a double-stranded DNA segment and rejoins the cleaved DNAs; mitomycin C, an inhibitor of DNA synthesis by introduction of cross-links into DNA at purine bases; bleomycin A5, which leads to double strand break.

<Response>: We thank the Reviewer for this insightful comment. The reviewer pointed out that the sentence on Line 351-355 (Original MS) is unclear and requires clarification. We have written separate sentences for each of the three molecules (etoposide, mitomycin C, and bleomycin A5) as shown below. We have also provided references for two points: the mechanism of inhibition and its relationship to nucleoli (the references were listed in Table 2, but they should also be included in the text). Furthermore, I have stated that all three molecules have been shown to induce nucleolar stress in animal cells.

Revised sentences:
[Line 360-367] As shown in Fig. 5A,C and Fig. S9A,B, we further examined the effects of the following reagents on leaf development in the *as2-1* and *as1-1* backgrounds: etoposide, a specific inhibitor of type II DNA topoisomerase (TOPII); mitomycin C, an inhibitor of DNA synthesis that introduces cross-links into DNA at purine bases; and bleomycin A5, which induces double-strand breaks (Kampranis et al., 1999; Yogo et al., 2012; Hutten et al., 2011; Burger et al., 2010; Boulon et al., 2010; González-Arzola, 2024). Etoposide, mitomycin C, and bleomycin A5 have also been shown to induce nucleolar stress in animal cells (Hutten et al., 2011; González-Arzola, 2024).

Line 489: The term "guardian angel" is defined as "An angel conceived as watching over or protecting a particular person or place" and "angel" is defined as "…a member of a class of celestial beings considered intermediate between God and humanity" (OED). I don't see how the description of a protein or gene can be congruent with these definitions. I suggest using different terminology.

<Response>: We appreciate the reviewer’s insightful comment. We agree that the phrase “a guardian angel” is not appropriate for a scientific paper.
We express as follows.

© 2026. Published by The Company of Biologists under the terms of the Creative Commons Attribution License (https://creativecommons.org/licenses/by/4.0/).

[Line 448-450] Together, these findings suggest that AS2 functions as a factor that mitigates nucleolar stress through formation of AS2 bodies during leaf development.

Table 2: G418 analog has a comment "N/A" but the text notes this chemical is an inhibitor of protein synthesis.

<Response>: We appreciate the Reviewer's comment on this point.
After consulting with a researcher specializing in chemistry, Dr. Ayato Sato, we learned that the G418 analog is a G-418 diastereomer and has a CAS registration number and IUPAC name. Therefore, we revised the description in **Table 1**. we also updated the molecular function in **Table 1** and the comments in **Table 2** as follows: Possible inhibitor of protein synthesis by targeting 70S and 80S ribosomes.

Table 1

G-418 diastereomer	possible inhibitor of protein synthesis by targetting 70S and 80S ribosomes	1192351-60-2	(1R,2R,4R,6S)-4,6-Diamino-3-({(5R)-2-amino-2-deoxy-5-[(1S)-1-hydroxyethyl]-α-D-xylopyranosyl}oxy)-2-hydroxycyclohexyl 3-deoxy-4-C-methyl-3-(methylamino)-D-arabinopyranoside	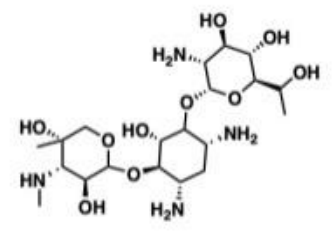 G-418 diastereomers

Table 2

molecules targeting ribosomes	**fusidic acid**	binding to prokaryotic and eukaryotic peptide elongation factors	Kim et al., 2023
	G-418	inhibitor of protein synthesis by targetting 70S and 80S ribosomes	Bidou et al., 2017; Heldin et al., 2023
	**G-418 diastereomer**	possible inhibitor of protein synthesis by targetting 70S and 80S ribosomes	N/A

We also learned that the lovastatin analog has a CAS registration number and IUPAC name, so we revised the description in Table 1. we also revised the molecular function in Table 1 and the comments in Table 2 as follows: Possible inhibitor of HMG-CoA reductase.
We will revise the text to use the same terminology as in the tables.
We would like to thank Dr. Ayato Sato for his advice on the chemicals used in this paper, including Tables 1 and 2, and on how to write the text. We have already mentioned this in the acknowledgements of the submitted manuscript, but we have added him as a co-author because he provided more detailed suggestions.

Figure 1C (and other graphs): The X and Y axes should be fully labelled. For Fig1C should be something like 10H-CPT concentration (uM) on the X axis and "leaf shape frequency" on the Y axis.

<Response>: We thank the Reviewer for this pertinent comment.
In accordance with the Reviewer's comment, The X and Y axes were fully labelled in **Fig. 1C, Fig. 5C,D. Fig. S9B.**

© 2026. Published by The Company of Biologists under the terms of the Creative Commons Attribution License (https://creativecommons.org/licenses/by/4.0/).

Fig. 1C

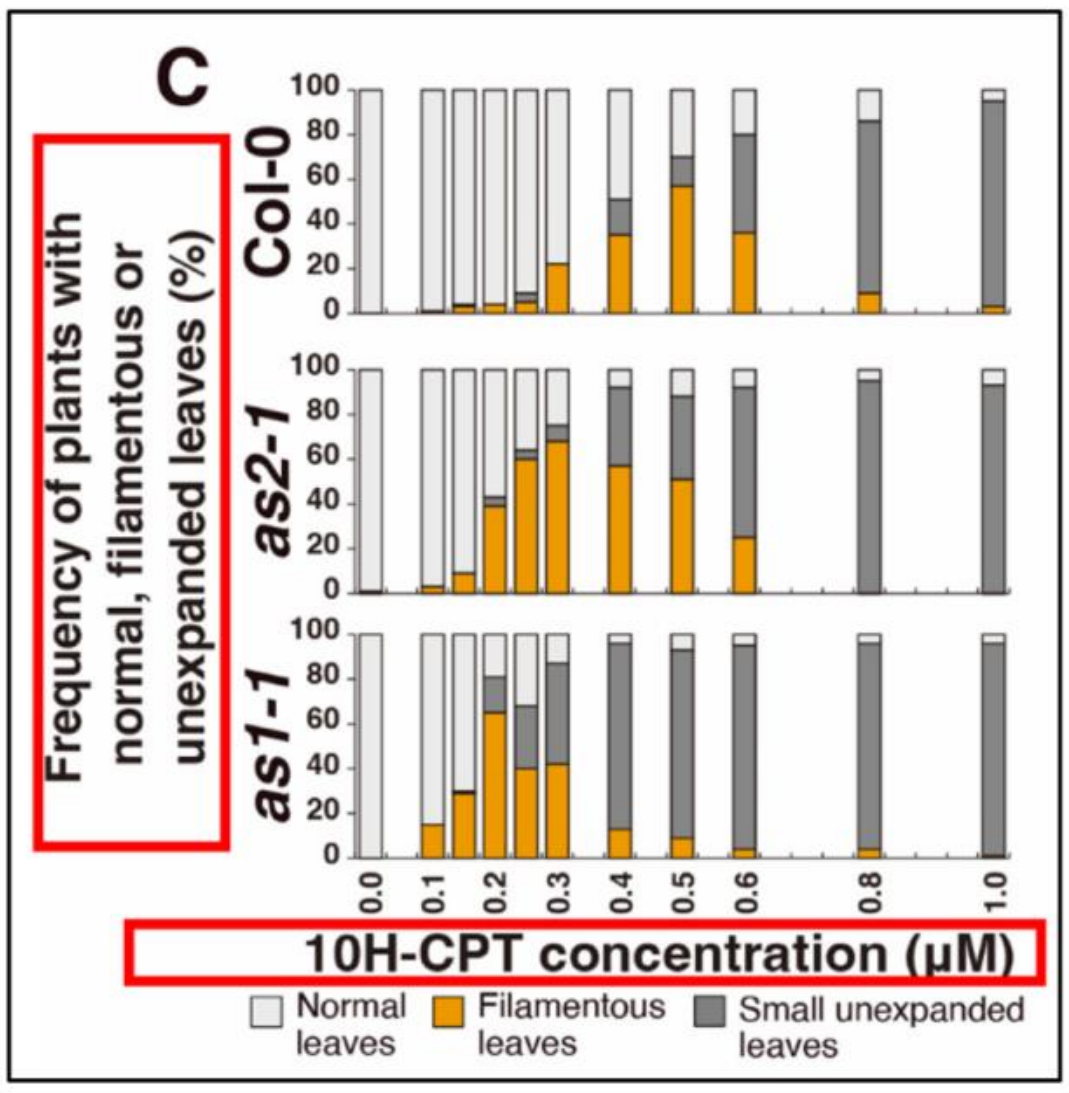

Fig. 5C,D

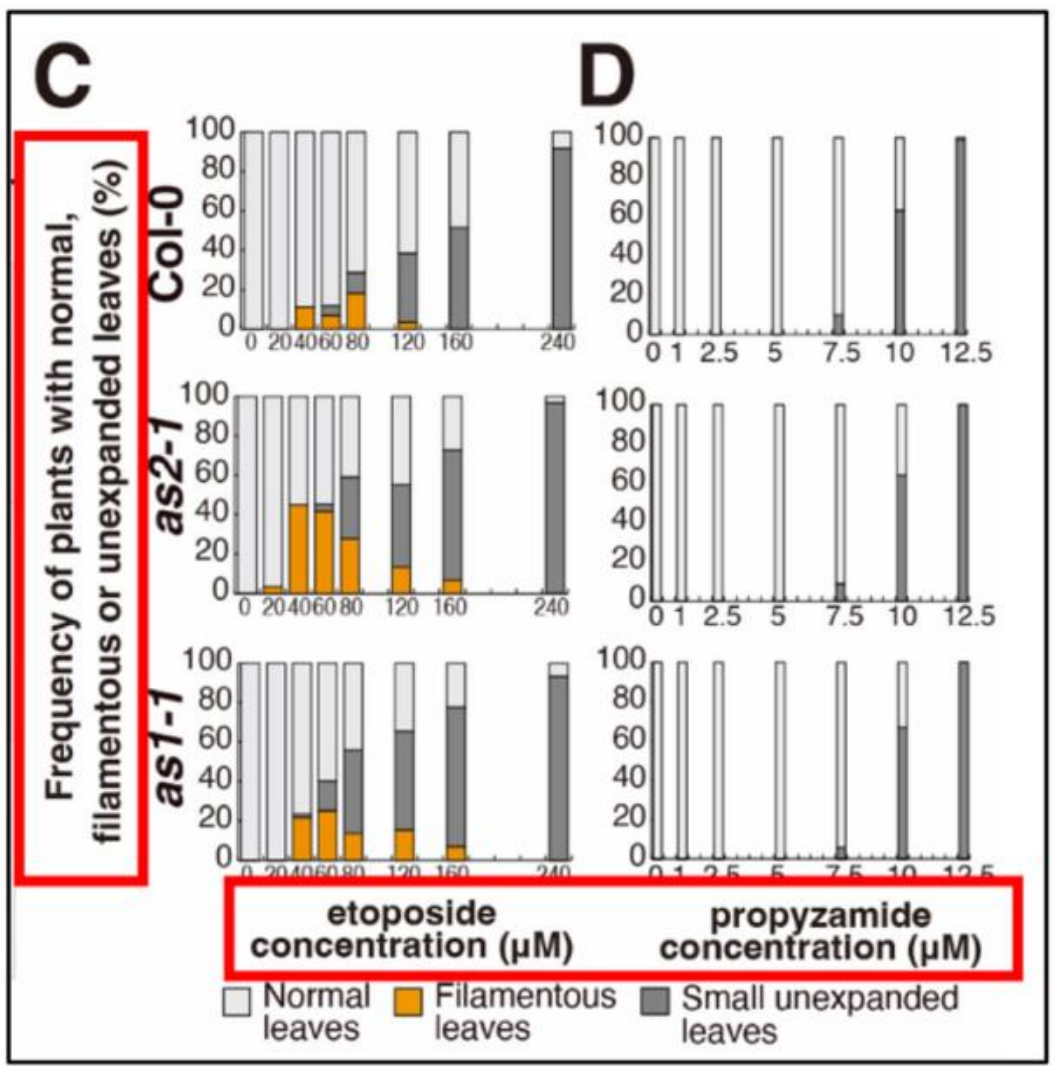

Fig. S9B

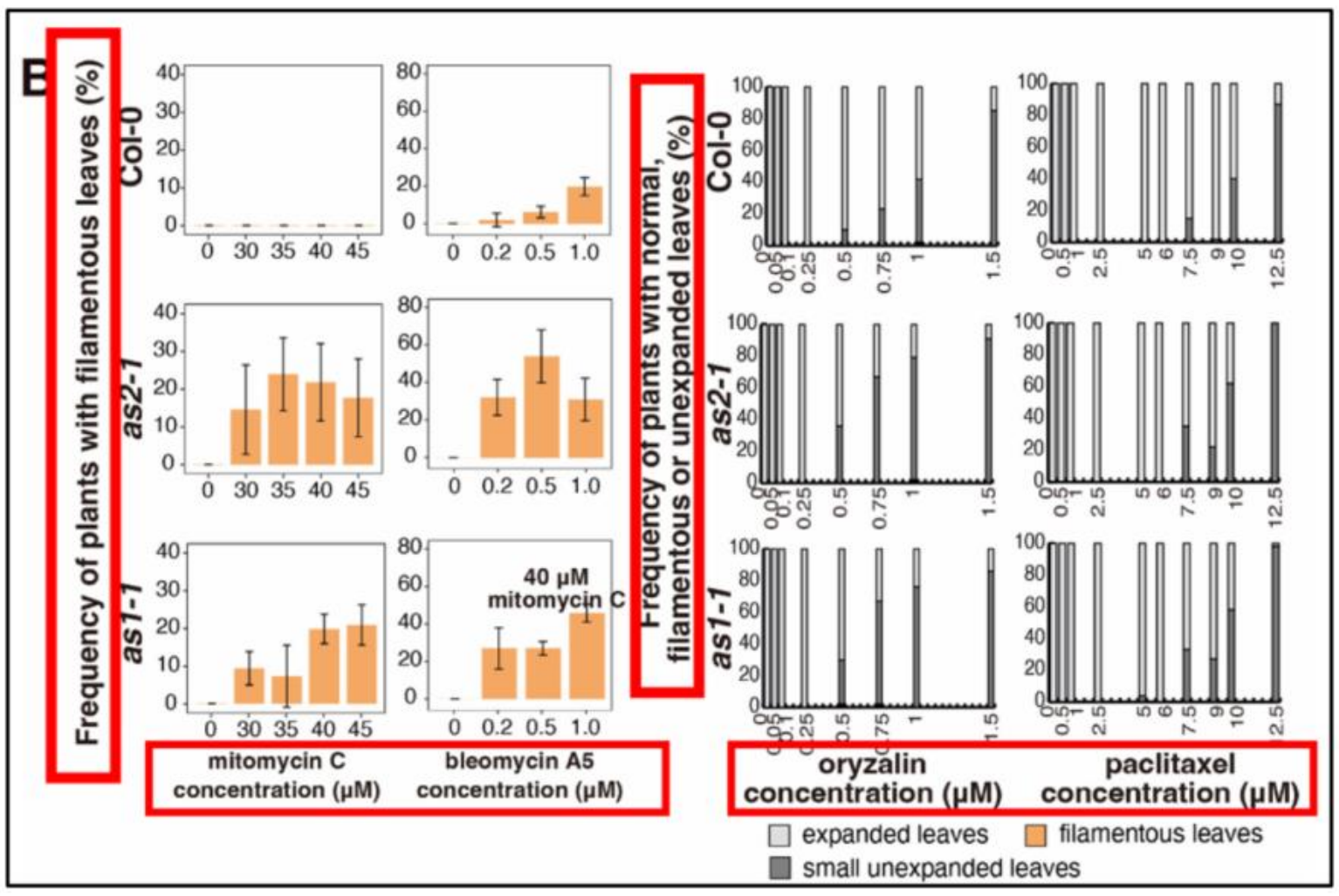

Figure 2A and other similar figures: the figure legend should state what the % indicates (is this the frequency of plants without radial leaves?) and what is in the brackets (is this the number of plants without radial leaves over the total number of plants examined?).

<Response>: We thank the Reviewer for this pertinent comment.
In accordance with the Reviewer's comment, the % and the () were indicated in **the figure legends of Fig. 1D, Fig. 2A,C,D, and Fig. 6A,B,C,D,E**

Figure legends for Fig. 1 [Line1168-1171] Values above each image indicate the frequency (%) of each leaf phenotype at each concentration (0, 0.2, or 0.5 μM). Numbers in parentheses represent the number of seedlings with the indicated phenotype/total number of seedlings examined.

Figure legends for Fig. 2 [Line1184-1187]; [Line1192-1194]; [Line1198-1201] Values above each image indicate the frequency (%) of each leaf phenotype. Numbers in parentheses represent the number of seedlings with the indicated phenotype/total number of seedlings examined.

Figure legends for Fig. 6 [Line1273-1274] Numbers above each photo show numbers of cells with

© 2026. Published by The Company of Biologists under the terms of the Creative Commons Attribution License (https://creativecommons.org/licenses/by/4.0/).

AS2 body patterning exemplified by the photo/numbers of cells examined.

Figure 3E: were the plants used for this qRT-PCR treated with 10H-CPT? Include the information in the figure.

<Response>: We did not treat with 10H-CPT.

The title is somewhat unwieldy. Possibly "Arabidopsis epigenetic factor AS2 attenuates nucleolar stress to establish leaf polarity by repressing a CDK inhibitor".

<Response>: We wish to express our strong appreciation to the Reviewer for his or her insightful comments on Title. We deleted "by camptothecin" and changed the title to follows.
"Arabidopsis epigenetic factor AS2 mitigates nucleolar stress and promotes flat leaf development by repressing a CDK inhibitor"

Reviewer 2:

The AS2 gene plays an important role in determining the upper (adaxial) epidermis. A chemical compound screen identified topoisomerase inhibitors (CPT and related compounds) as agents that inhibit adaxialisation in an as2 mutant background (this was confirmed using abaxial reporter lines). These observations were subsequently confirmed genetically using available topoisomerase mutants, which demonstrated that this was caused by increased transcript levels of the ETT/ARF3 gene. Most interestingly, through transcriptome analysis, the cyclin-dependent kinase gene KRP5 was identified as a downstream target, a finding that was supported by genetic data.

Overall, this manuscript is rich in data and makes a robust and novel contribution to our understanding of leaf polarity regulation in Arabidopsis thaliana, presenting novel insights into the role of AS2 in regulating leaf polarity via CDK inhibition. The work logically progresses from chemical screening to genetic interactions.

<Response>: We wish to express our appreciation to the Reviewer 2 for his or her insightful comments, which have helped us significantly improve the paper.

Major comments:

1. While I find the macroscopic and RT-PCR data on loss of abaxialisation convincing, the role of cell division in the process is currently overinterpreted. Currently, the authors rely on CYCB1;2-GUS reporter activity in the presence or absence of topoisomerase activity. A better approach would be to make a comparison in the as2 krp5 top1 (or as2 krp5) background. As introducing this marker line into these mutant backgrounds might take a long time, an alternative would be to use leaf clearing to estimate the number of cells in both epidermal tissues.

<Response>: We wish to express our appreciation to the Reviewer for the comment on this point. As the reviewer pointed out, it will be important to investigate how *KRP5/ICK3*, a downstream factor of AS2-AS1, is involved in cell division. The experiment measuring CYCB1;2-GUS reporter activity was conducted simply to examine cell division activity,

As shown in **RESULTS, Fig. 4H**, and **Fig. S8C,** we have now obtained a plant (*CYCB1;2p-GUS/as2 krp5*) by crossing the *CYCB1;2p-GUS* reporter line with *as2 krp5* and examined GUS activity.
As reviewer 2 pointed out, we intend to carefully rewrite the text to avoid overinterpreting the role of cell division in the process.

[Line 347-351] When *CYCB1;2p:GUS/as2-1* plants were treated with 10H-CPT, GUS activity was detected at low levels in the rod-shaped leaves (Fig. 4H). Several *CYCB1;2p:GUS/as2-1 krp5-1* plants formed true leaves, in which GUS activity was detected in the petioles and vascular bundles (Fig. 4H and Fig. S8C).

© 2026. Published by The Company of Biologists under the terms of the Creative Commons Attribution License (https://creativecommons.org/licenses/by/4.0/).

These results suggest that *AS2* and *TOP1a* promote cell proliferation in developing leaves by repressing *KRP5*/*ICK3*, which is required for the formation of flat, expanded leaves. However, Fig. S8C shows no difference in signal between CPT-treated *CYCB1;2p:GUS*/*as2-1* and CPT-treated *CYCB1;2p:*GUS/*as2-1 krp5-1*, suggesting that *KRP5*/*ICK3* alone does not regulate cell proliferation.

Fig. 4H

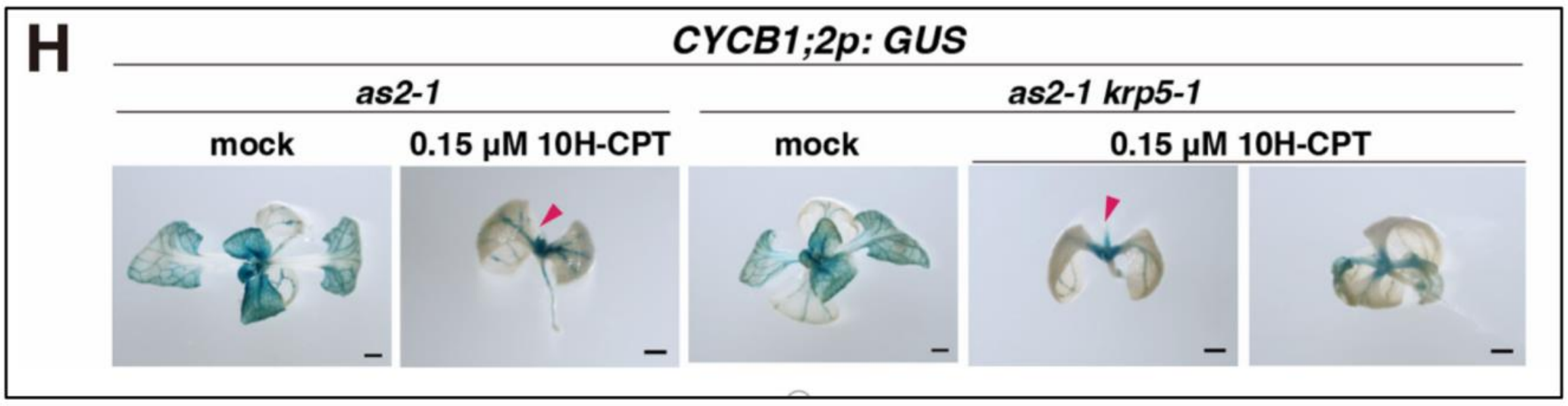

Fig. S8C

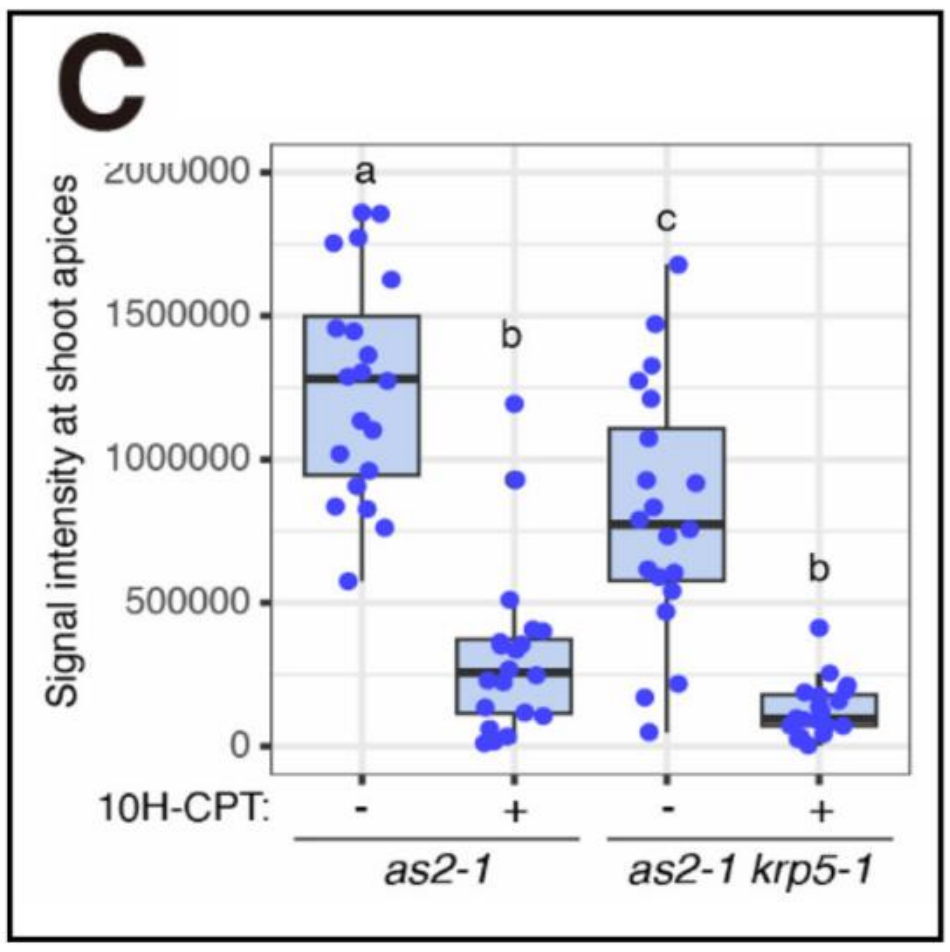

Major comment 2: A related shortcoming is the absence of data on a KRP5 reporter line, despite such a line being available in the field (see, for example, the cited Echevarría et al. paper recently published in Nature Plants). Does it demonstrate any abaxial-adaxial specificity? Alternatively, such data could be extracted from recent leaf scRNA-seq experiments that have been published.

<Response>: We wish to express our deep appreciation to the Reviewer for her or his insightful comment on this point.

As pointed out by the reviewer, we have examined GUS activity in Col-0 using the *KRP5p:KRP5-GUS* reporter line provided by Dr. Umeda. As shown in **Supplementary Fig. S5**, weak GUS activity was observed in the internal tissues of young leaf primordia at the shoot apex. On the other hand, we have already reported that *AS2* is expressed in the adaxial region of leaf primodia, including the epidermal cells (Iwakawa et al., 2007). These results support the microarray finding that *AS2* represses the expression of *KRP5*/*ICK3* gene during leaf development.

© 2026. Published by The Company of Biologists under the terms of the Creative Commons Attribution License (https://creativecommons.org/licenses/by/4.0/).

Fig. S5

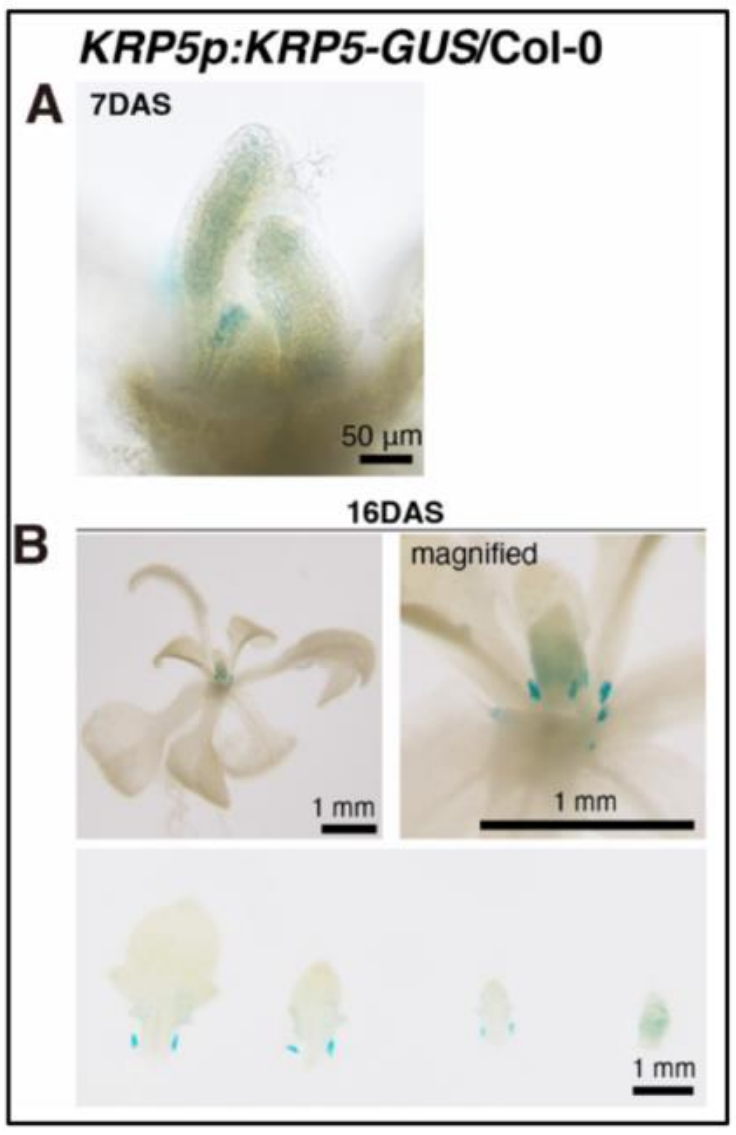

We analyzed scRNA-seq data (Zhang et al., Dev. Cell 2021). We added these data in **Fig. 3, Fig. S4**, and **RESULTS [Line 295-304]**.

Using a published scRNA-seq dataset of Arabidopsis vegetative shoot apices and leaves (BioProject PRJCA003094; Zhang et al., 2021), we re-clustered and visualized cell subsets expressing *KRP5/ICK3*, *ETT/ARF3*, *BP*, and *AS2*, designated as *KRP5*+, *ETT*+, *BP*+, and *AS2*+, respectively, via UMAP (Fig. 3C–F, Fig. S4). Ratios of co-expressing cells revealed that *KRP5/ICK3* was co-expressed with *ETT/ARF3* (16.8%) and *BP* (12.5%), but rarely with *AS2* (1.9%), suggesting that *KRP5/ICK3* may be expressed in very early leaf primordia (Fig. 3G). Conversely, *AS2* was minimally co-expressed with *KRP5/ICK3* (2.5%), *ETT/ARF3* (2.7%), and *BP* (3.0%) (Fig. 3G). These data reinforce prior microarray evidence indicating that *AS2* acts as a repressor of these genes during leaf development.

We also added scRNA-seq data processing in **MATERIALS AND METHODS as shown in [Line 701-741]**.
These results revealed the expression of *KRP5/ICK3*, *ETT/ARF3*, and AS2 in wild-type plants. The results suggest that *KRP5/ICK3* may be expressed in cells of very early leaf primordia expressing *ETT/ARF3* and *BP,* but not *AS2*. These results support the prediction by KB-FuzzyART that AS2 represses the expression of *ETT/ARF3* and *KRP5/ICK3* during leaf development.

© 2026. Published by The Company of Biologists under the terms of the Creative Commons Attribution License (https://creativecommons.org/licenses/by/4.0/).

Fig. 3C,D,E,F,G

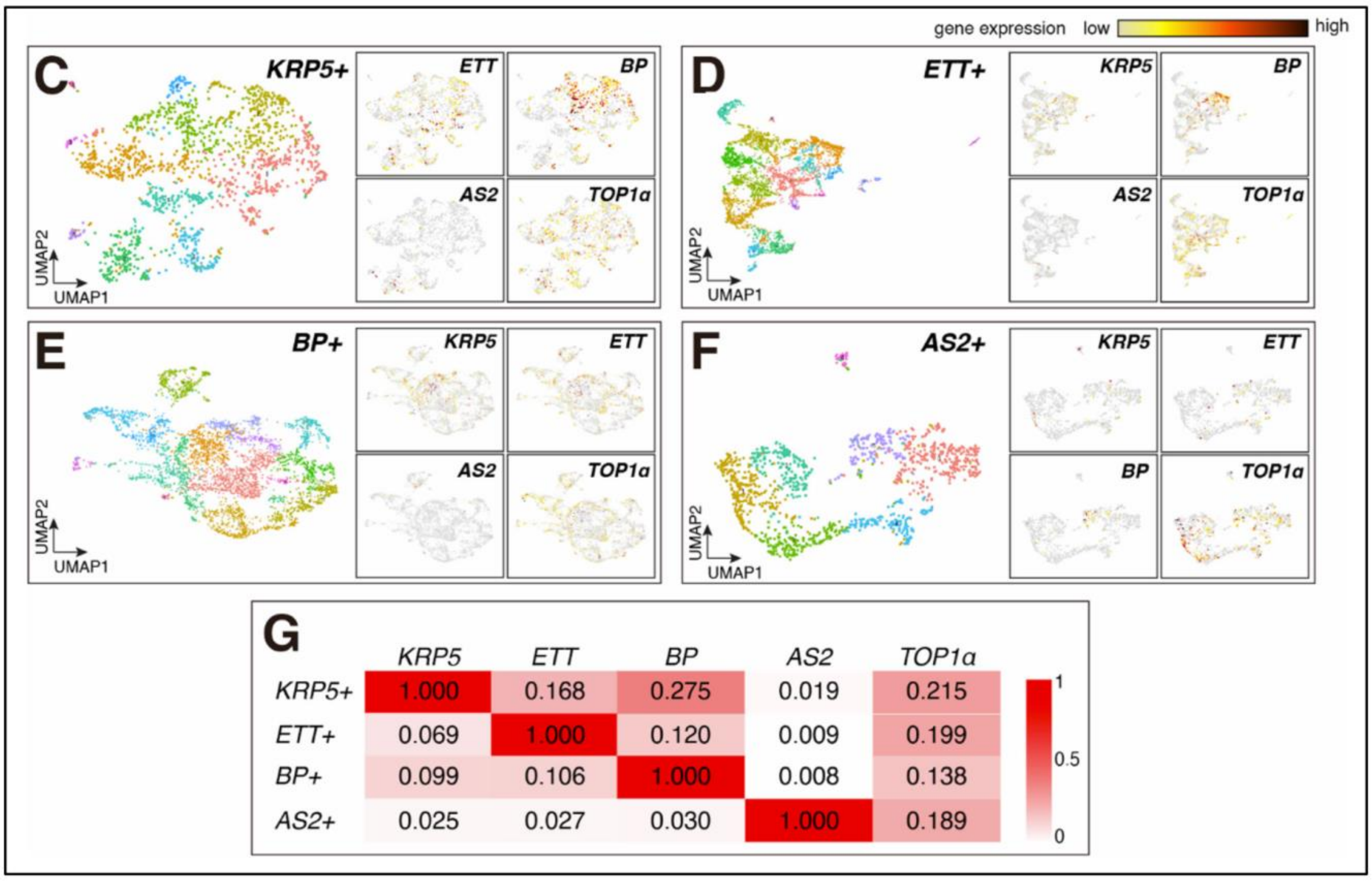

© 2026. Published by The Company of Biologists under the terms of the Creative Commons Attribution License (https://creativecommons.org/licenses/by/4.0/).

Fig. S4

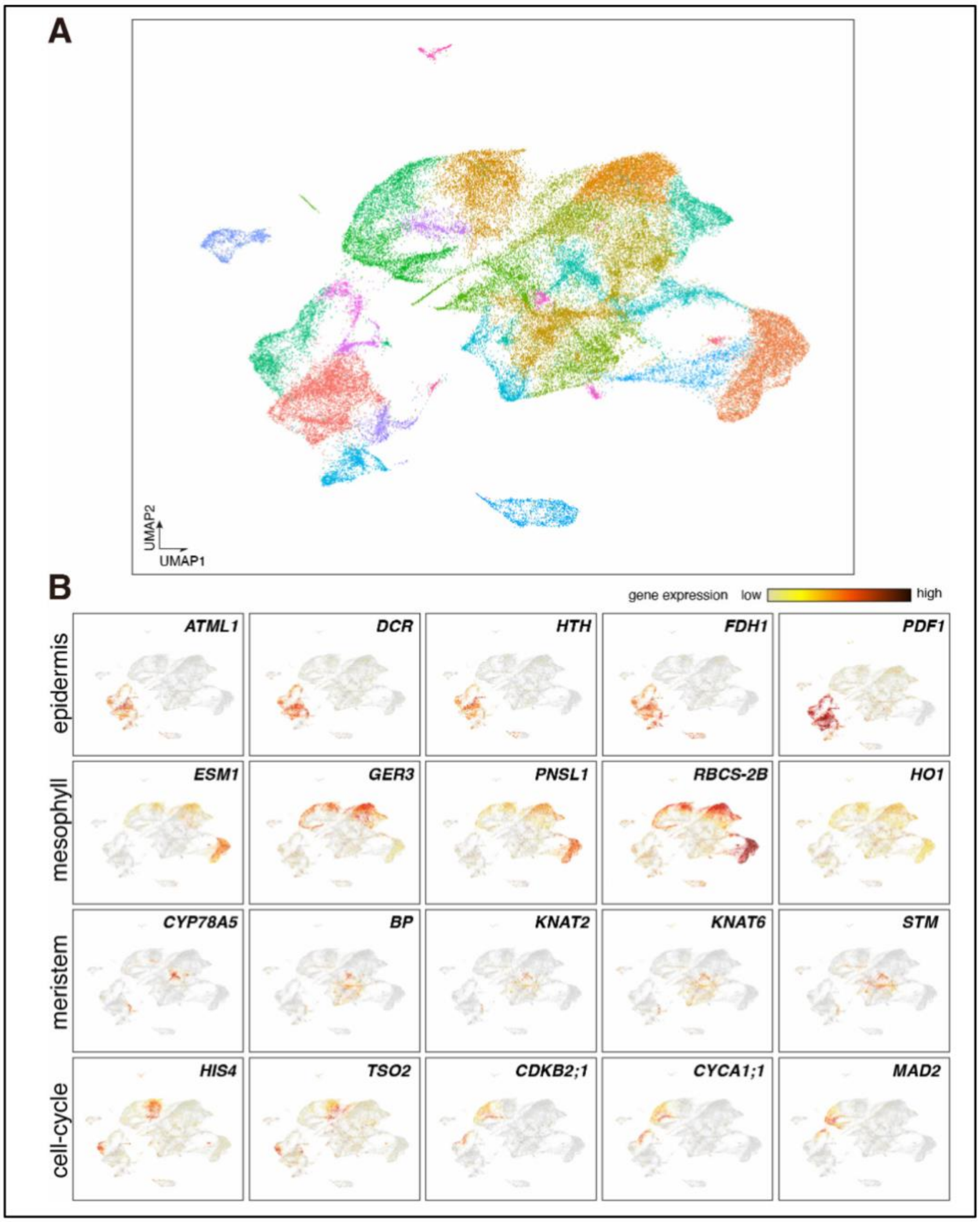

Major comment 3: Another shortcoming is that the authors ignored the possible effects of endoploidy, a process known to be controlled by KRP genes. It should also be noted that many of the mutants previously found to act together with AS2 in determining leaf adaxialisation possess a ploidy link (e.g. ribosome and nucleosome components).

<Response>: We appreciate the Reviewer's comment on this point.
It is very interesting that many mutants contain genes related to ploidy (ribosome and nucleosome components), which previously were found to cooperate with *AS2* in determining leaf adaxialization. We have reported that the AS2 protein partially co- localizes with chromocenters containing 45S rDNA repeats in the perinucleolar region (Luo et al., Plant J. 2020). It has been reported that KRP5/ICK3 links to chromatin structure and endoreduplication (Jegu et al., 2013).

Therefore, we added the sentence as follows.

[Line 480-482] Furthermore, KRP5/ICK3 has been reported to positively regulate endoreduplication (Jegu et al., 2013; Wen et al., 2013). Therefore, AS2 may promote cell proliferation by suppressing KRP5-mediated endoreduplication.

Secondary comments and questions:

© 2026. Published by The Company of Biologists under the terms of the Creative Commons Attribution License (https://creativecommons.org/licenses/by/4.0/).

Comment 1. Some sentences in the introduction are long and complex, making them hard to follow, e.g. lines 67-70 and 100-104.
Line 67-70:
Original sentence: The initial leaf primordium is radial with abaxial cells, and some parts of the basal cells more adjacent to the top of the radially shaped SAM could initiate to differentiate into adaxially fated cells, establishing the leaf adaxial-abaxial polarity (Byrne et al., 2000; Iwakawa et al., 2002; Bowman 2002; Newman et al., 2002; Tsukaya 2006; Fukushima & Hasebe 2014).

<Response>: In accordance with the Reviewer's comment, we revised the sentences (first paragraph in INTRODUCTION) as follows.

Revised sentences:
[Line 68-75] During early leaf development, adaxial fate is established in cells adjacent to the SAM, thereby generating adaxial–abaxial polarity, and subsequent cell proliferation along the medial–lateral axis gives rise to a flat leaf blade (Byrne et al., 2000; Iwakawa et al., 2002; Bowman, 2002; Newman et al., 2002; Tsukaya, 2006; Fukushima and Hasebe, 2014). Although antagonistic interactions between adaxial and abaxial determinants are essential for leaf flattening, the mechanisms by which these factors regulate cell division remain unclear (Wu et al., 2008; Iwasaki et al., 2013; Fukushima and Hasebe, 2014; Machida et al., 2015; Husbands et al., 2015).

Line 100-104:
Original sentence: Each of as2 and as1 single mutants exhibits a phenotype of weakly impaired leaf adaxialization (Iwakawa et al., 2007), additionally, when other gene mutations in the as2 or as1 background often exhibit filamentous (rod-shaped) leaves, which show complete loss of leaf adaxialization (Pinon et al., 2008; Yao et al., 2008; Szakonyi et al., 2011; Horiguchi et al., 2011; Iwasaki et al., 2013; Machida et al., 2015).

<Response>: In accordance with the Reviewer's comment, we revised the sentences as follows.

Revised sentences:
[Line 94-98] Both *as2* and *as1* single mutants show mild defects in leaf adaxialization (Iwakawa et al., 2007), whereas additional mutations in these backgrounds often produce filamentous leaves that lack adaxial identity (Pinon et al., 2008; Yao et al., 2008; Szakonyi et al., 2011; Horiguchi et al., 2011; Iwasaki et al., 2013; Machida et al., 2015).

Comment 2. Define 'nucleolar stress' in the introduction rather than the discussion section.

<Response>: We appreciate the Reviewer's comment on this point. We insert the following sentences in INTRODUCTION.

[Line 107-110] Because disruption of ribosome biogenesis causes nucleolar stress, and a plant-specific nucleolar stress response pathway has recently been identified (Ohbayashi et al., 2017; Ohbayashi & Sugiyama, 2018), the molecular relationship between AS2-AS1, modifier proteins, and nucleolar stress is an important unresolved issue.

Comment 3. Section 363-369: (related to Figure 5): might a dose of propyzamide that is too high have been used to conclude that impaired M phase progression does not trigger a similar phenotype? Given the bleached leaves (compared to the etoposide plants), it appears that these plants are dying. A similar comment applies to the RT-PCR data, for which the figure legend does not specify the length of treatment. Overall, the legends could provide more explanation to enable standalone understanding. Overall, the rationale for choosing certain concentrations or compounds could be better justified.

<Response>: We appreciate the Reviewer's comment on this point.

© 2026. Published by The Company of Biologists under the terms of the Creative Commons Attribution License (https://creativecommons.org/licenses/by/4.0/).

At higher concentrations of propyzamide, most plants died, so we were unable to measure the transcript levels of these genes. Therefore, we measured them at 5 µM in intact plants. We also conducted experiments at concentrations lower than 5 µM. We found that rod shape of leaves did not generate in either case.
We showed propyzamide all-dose-phenotype.

Fig. S4

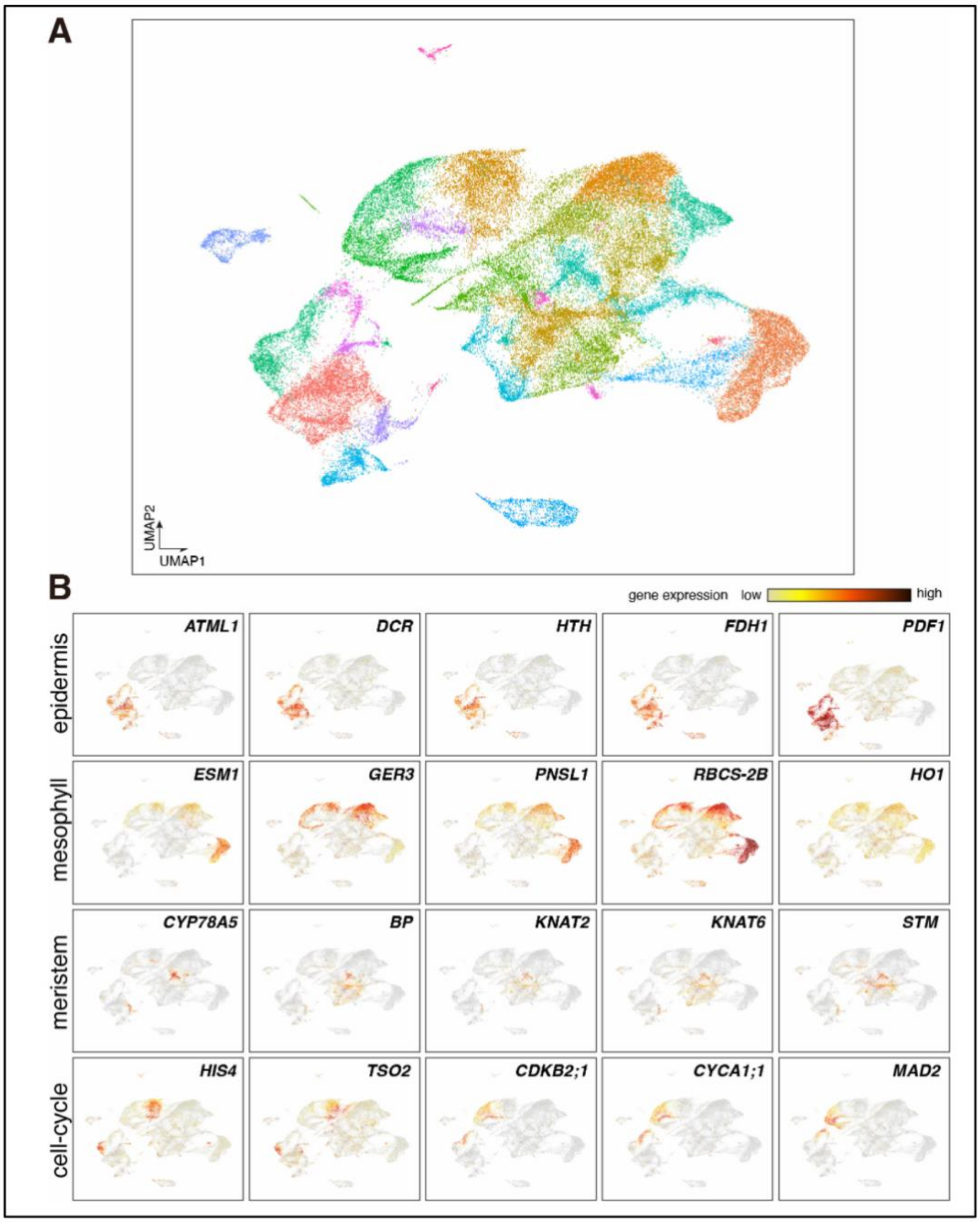

The timing of each compound addition was described in "**Chemicals**" section of **MATERIALS AND METHODS**and Figure legends.

[531-540] All chemical treatments (except 17ß-estradiol) were performed by supplementing MS agar medium with the indicated compounds. Seeds were sown directly on chemical-containing MS medium and grown for 7 (for AS2-YFP and NUC1-GFP imaging, Fig. 6A,B,C,D), 13 (for *CYCB1;2p:GUS*, Fig. 4G,H, Fig. S8), 14 (for observation of *ETTp:GUS*, *BPp:GUS*, *FILp:GFP*, *krp5-1* (*krp5-4* or *krp2-5*) *as2-1* treated with 10H-CPT, all the qRTp-PCR analysis, Fig. 1F,G, Fig. S1, Fig. 4A,B, Fig. S6B, Fig. S7C,D, Fig. 1E, Fig. 5E-G, Fig. 6F-H, Fig. S3F, Fig. S7B), or 21-22 days (all the other leaf phenotypic analysis using 10H-CPT, etoposide, mitomycin C, bleomycin A5, propyzamide, oryzalin, paclitaxel, Fig. 5A-D, Fig. S9).

We also described the rationale for choosing certain concentrations or compounds in **RESULTS**

© 2026. Published by The Company of Biologists under the terms of the Creative Commons Attribution License (https://creativecommons.org/licenses/by/4.0/).

[Line 367-370] The *as2-1* and *as1-1* mutant plants that were grown in the presence of these molecules formed filamentous leaves, although Col-0 plants scarcely formed, as observed upon the treatment with 10H-CPT (Fig. 5A,C, Fig. S9A,B).

Comment 4: Is the nucleolar bodies phenotype still present in the top1 as2 sriw1 mutant?

<Response>: The reviewer's point is very important. Unfortunately, we have not examined AS2 bodies in the *top1 as2 sriw1* mutant, yet. Mutant AS2 proteins (mutations in the AS2/LOB domain) cannot form AS2 bodies. (Luo et al., Plant J. 2020 Fig. 2; Ando et al., et al., Plants 2023 Fig. 2, as shown below). Therefore, even if the *sriw1* mutation suppresses the rod-shaped phenotype, we believe that the inability to form an AS2 body remains unchanged. The leaf phenotype of the *top1 as2 sriw1* mutant is similar to that of the *as2* single mutant. On the other hand, mutations in the *ETT/ARF3* gene suppress the leaf phenotype of the *as2* mutation, resulting in a leaf phenotype that is almost wild-type (Iwasaki et al., Development 2013). Therefore, we believe that the *SRIW1/ANAC082* pathway is different from the *ETT/ARF3* pathway for leaf formation (Fig. 7C).

SRIW1/ANAC082, which is involved in the nucleolar stress response, is a protein with an NAC domain, and the Ohbayashi-Sugiyama group is currently conducting vigorous research on it. We believe that clarifying the molecular relationship between SRIW1/ANAC082 and AS2 will be important in the formation of leaves in angiosperms.

NOTE: Figure provided for reviewer has been removed. It showed Figure 2(a) from Luo, L., Ando, S., Sakamoto, Y., Suzuki, T., Takahashi, H., Ishibashi, N., Kojima, S., Kurihara, D., Higashiyama, T., Yamamoto, K.T., Matsunaga, S., Machida, C., Sasabe, M. and Machida, Y. (2020). The formation of perinucleolar bodies is important for normal leaf development and requires the zinc-finger DNA-binding motif in Arabidopsis ASYMMETRIC LEAVES2. Plant J. 101, 1118–1134. doi:10.1111/tpj.14579. We have removed unpublished data that had been provided for the referees in confidence.

Luo et al., Plant J. 2020 Fig. 2 https://doi.org/10.1111/tpj.14579

The zinc-finger motif is required for localization of AS2 bodies and the ICG and LZL regions are required for nuclear localization of AS2. DNA constructs encoding mutant proteins were fused to the sequence encoding to the N-terminus of YFP and these fused constructs were linked to the estrogen-inducible promoter.

NOTE: Figure provided for reviewer has been removed. It showed Figure 2A, 2B and 2E from Ando, S., Nomoto, M., Iwakawa, H., Vial-Pradel, S., Luo, L., Sasabe, M., Ohbayashi, I., Yamamoto, K.T., Tada, Y., Sugiyama, M., Machida, Y., Kojima, S. and Machida, C. (2023). Arabidopsis ASYMMETRIC LEAVES2 and nucleolar factors are coordinately involved in the perinucleolar patterning of AS2 bodies and leaf development. Plants 12, 3621–3640. doi:10.3390/plants12203621. We have removed unpublished data that had been provided for the referees in confidence.

Ando et al., Plants 2023 Figure

https://w.mdpi.com/2223-7747/12/20/3621

Amino acid residues in the ZF motif and the conserved glycine of the ICG sequence are required for the formation of AS2 bodies.

Reviewer 3:

SUMMARY OF THE ADVANCE MADE IN THIS PAPER AND ITS POTENTIAL SIGNIFICANCE TO THE FIELD

© 2026. Published by The Company of Biologists under the terms of the Creative Commons Attribution License (https://creativecommons.org/licenses/by/4.0/).

Nakagawa et al. described the involvement of TOPI function in regulating leaf morphology, through the interaction with AS2, an epigenetic regulator. Multiple compounds with the effect of enhancing the as2 phenotype were isolated from chemical screening, and among them, the authors focused on the mode of action of camptothecin

, a well-known topoisomerase I inhibitor. Indeed, the top1α mutation enhanced as2 phenotype to induce filamentous leaves and seedling lethality. The authors also found that ETT/ARF3 and/or KRP5/ICK3 genes were upregulated in as2, top1α, and as2 top1α mutants, and that the mutations of them can partially suppress the phenotypes of as2 top1α as well as the camptothecin effects, indicating that the severe leaf morphology and lethal phenotype in as2 top1α would be attributable to ETT/ARF3 and KRP5/ICK3. They also demonstrated that the induction of genomic stress, such as DNA double-strand breaks, rather than cell cycle progression inhibition, enhanced the upregulation of ETT/ARF3 and KRP5/ICK3 expression and the formation of filamentous leaves in as1 or as2 mutants. Finally, the top1α mutation and camptothecin disturbed the number and morphology of AS2 bodies. Taken together with the suppression effect of sriw1 mutation, in which the regulator of nucleolus stress response is defective, the authors concluded that AS2 would function as a mediator of TOPI-related nucleolus stress response to the determination of leaf morphology. Overall, the study was well-executed and undoubtedly contributes to a deeper understanding of the effects of nuclear (or nucleolar) stress on leaf morphogenesis.

<Response>: We wish to express our appreciation to the Reviewer 3 for her or his insightful comments, which have helped us significantly improve the paper.

SUGGESTIONS TO AUTHORS

While I believe important findings are presented in the manuscript, to support the conclusion, several questions remain. The major points are as follows:

1. Title and discussions: The leaf morphogenesis process related to TOPI and KRP5 may not be the stage of polarity determination itself, but rather the stage of cell division control that occurs after polarity determination. Or, do the authors think that the control of cell division itself is included in leaf polarity determination? (Based on the last sentence of the first paragraph in Introduction, I understood that these stages are considered separately, but is that incorrect?) Throughout the paper's title and discussions, the terms like leaf morphogenesis and polarity determination are used interchangeably sometimes. However, to place the AS2-ETT-KRP5 pathway precisely on the leaf development, I would like the authors to clarify this once more. In other words, the current title is highly misleading. I request a careful reconsideration, including the phrasing used throughout the discussion.

<Response>: We appreciate your insightful question and comment.
We propose that medio-lateral polarity is established through cell division. This indicates that the regulation of cell division plays a crucial role in determining leaf polarity, leading to the formation of flattened leaves. We will clarify this distinction and revise the terminology throughout the manuscript accordingly.
However, in this study, we demonstrated that flattened leaves can form even under nucleolar stress when AS2 and AS1 (a PHAN homolog) repress abaxial genes and then the CDK inhibitor KRP5/ICK3 during Arabidopsis leaf development.
Nevertheless, we have not yet analyzed the properties of the genes (factors) involved in establishing medio-lateral polarity.
Therefore, we revised the title to avoid using the word “polarity” as follows: **“Arabidopsis epigenetic factor AS2 mitigates nucleolar stress and promotes flat leaf development by repressing a CDK inhibitor”**

2. The experimental verification of the involvement of TOPI and KRP5 in leaf morphology is an excellent result. However, the view that "ETT is a target of AS2, and KRP5 would act as its downstream regulator" and that "nucleolar stress is involved in as2 leaf morphogenesis" have already been reported by the authors, i.e. these points are not novel. To ensure the novelty of this

© 2026. Published by The Company of Biologists under the terms of the Creative Commons Attribution License (https://creativecommons.org/licenses/by/4.0/).

paper, it is necessary to examine how the top1α mutant or camptothecin affect nucleolar structure using markers other than AS2-YFP. Furthermore, it is necessary to clarify whether ETT-mediated induction of KRP5 expression occurs through the direct transcriptional activation by ETT.

<Response>: We appreciate your insightful comments.
We believe that the novelty of this paper lies in the following two points:
First, it revealed that AS2 is involved in formation of flat leaves by suppressing the CDK inhibitor KRP5/ICK3, and second, it revealed that AS2 functions to alleviate nucleolar stress during leaf development.

First, we previously demonstrated that *ETT/ARF3* is a direct target of AS2 using ChIP analysis, transcriptome analysis, and genetic analyses (Iwasaki et al., Development 2013). Furthermore, transcriptome meta-analysis identified *KRP5/ICK3* as a candidate gene regulated by AS2. We also genetically demonstrated that *KRP5/ICK3* is downstream of *ETT/ARF3* (Takahashi et al., Plant Cell Physiol. 2013). Here, we demonstrate for the first time that AS2 induces flat leaf formation by suppressing the CDK inhibitor KRP5/ICK3.

Secondly, we have previously shown that a double mutant of a nucleolar stress-inducing mutant and an *as2* mutant forms rod-shaped leaves, and that the shape of AS2 bodies formed around the nucleolus is altered in the nucleolar stress-inducing mutant (Ando et al., Plants 2023). Here, we demonstrate for the first time that the rod-shaped leaf phenotype is suppressed by *sriw1*, a mutant of *ANAC082* involved in nucleolar stress response. We believe that this point is the novelty of this paper.

However, it remains unclear whether *ETT/ARF3* directly regulates *KRP5/ICK3*. ChIP analysis of *ETT/ARF3* using floral tissue has been reported, but *KRP5/ICK3* was not included as a direct target candidate (Simonini et al., Plant Cell 2017). It will be important to identify genes that directly regulate *KRP5/ICK3*. Therefore, it is necessary to clarify the molecular mechanism by which *KRP5/ICK3* is regulated during leaf development, which is a future challenge.

We examined 35S:NUC1-GFP/Col-0 plants treated with 0.1, 0.15 and 0.4 μM 10H-CPT at the same condition of Fig. 6A.
As shown in **Fig. 6C** and **[Line 401-404]**, In NUC1-GFP transgenic plants, the GFP signal was uniformly distributed throughout the nucleolus (Fig. 6C). However, upon treatment with 10H-CPT, the GFP signal exhibited a non-uniform distribution within the nucleolus (Fig. 6C), suggesting that 10H-CPT induces structural changes in the nucleolus.

Fig. 6C

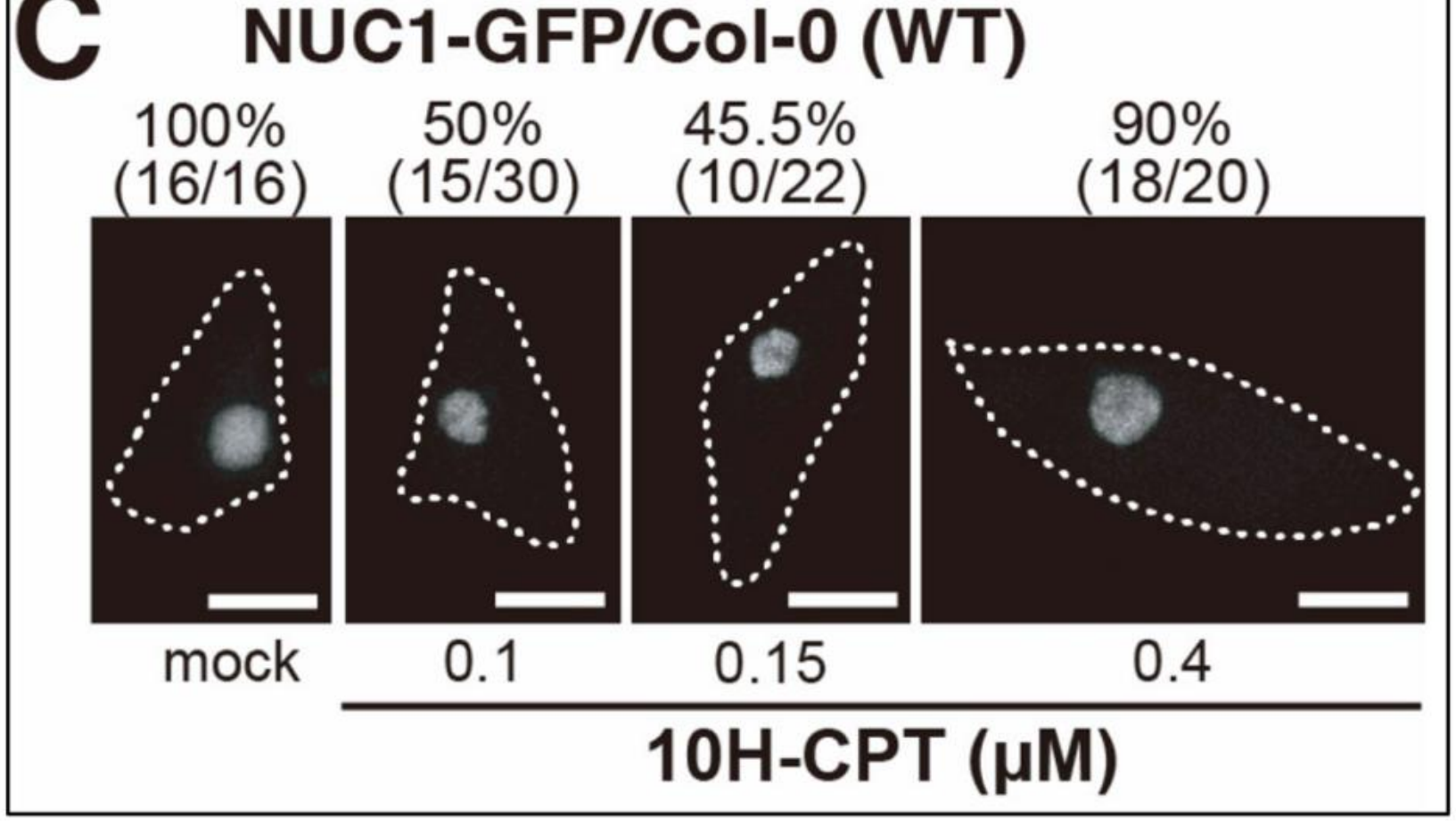

© 2026. Published by The Company of Biologists under the terms of the Creative Commons Attribution License (https://creativecommons.org/licenses/by/4.0/).

We have observed the formation of multiple small nucleolar clusters (NUC1-GFP) after exposure to a higher concentration (100 µM 10H-CPT), a phenomenon previously reported in animal cells.

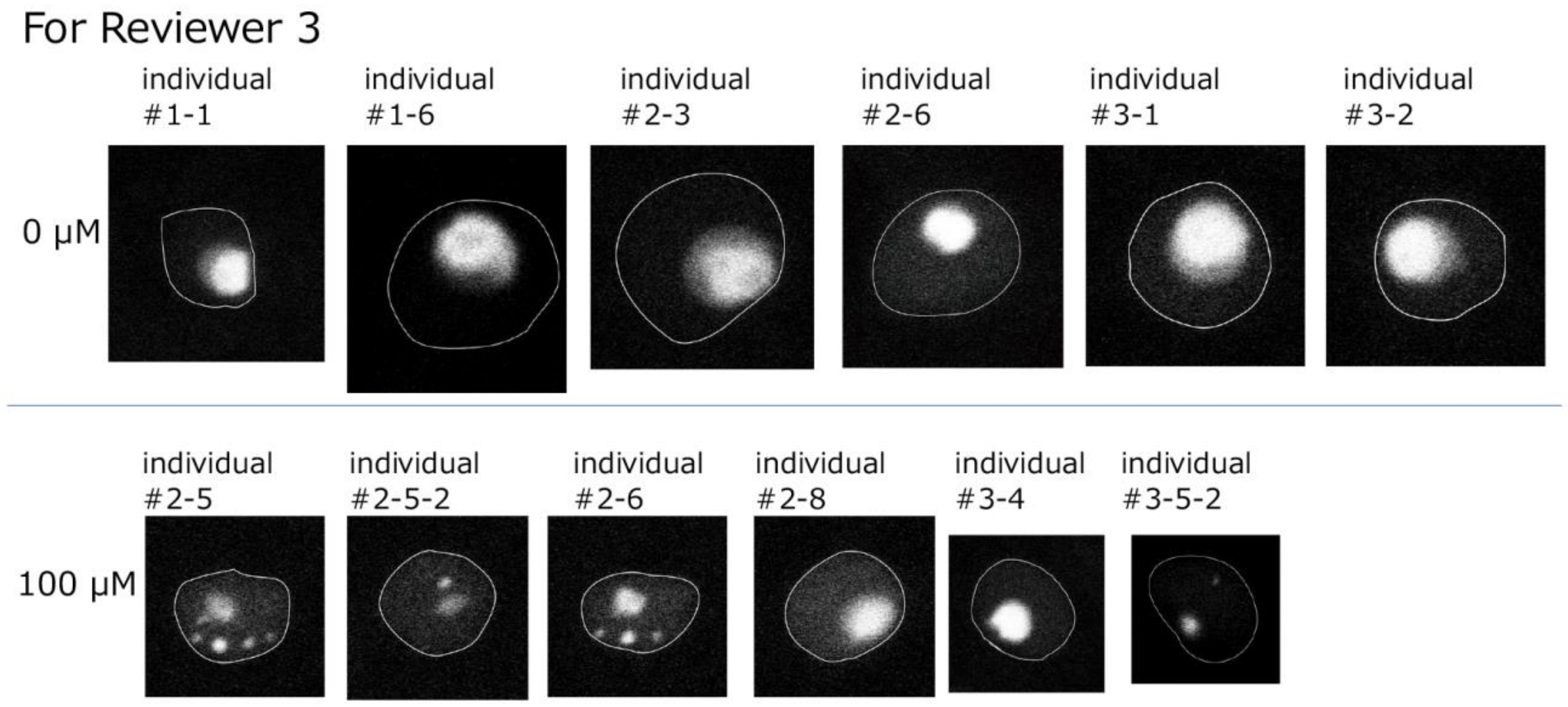

Observation of abnormal nucleoli in 10H-CPT-treated Arabidopsis root.

Shown photos are root tips of 3-day-old 35Spro:Nuc1-GFP treated with 0 or 100 µM 10H-CPT overnight.

In 0 µM-treated root, a large nucleolus per a nuclei are observed.

On the other hand, in 100 µM-treated root, GFP signals are weak, or 2-5 signals are observed.

We are currently analyzing electron microscopy images of nucleoli in various mutants, including the *rid2* mutant, which has been reported to induce nucleolar stress. We are also investigating the intranuclear localization of 45S rDNA, which partially colocalizes with AS2 bodies, in *rid2* and other mutants.

Understanding the structural changes in nucleoli and 45S rDNA under nucleolar stress is extremely important and will be a key focus of our future research.

3. The authors state that AS2 and TOP1 "synergistically" regulate cell proliferation (e.g. line 308, 319, 344). But where is the "synergy"? Since ETT expression levels are approximately twice wild-type in as2 and top1α single mutants, and 4-5 times in double mutants, this is simply additive, not synergistic, I guess. Furthermore, it is problematic that KRP5 expression in top1α single mutants has not been examined. Please investigate the KRP5 expression in top1α.

Even in the final model presented in Fig. 7, TOP1 is simply located at the upstream of AS2. If the effect is synergistic (or even additive), this contradicts the authors' claim. This point also requires either a revision of the description or a modification of the model.

<Response>: We thank the Reviewer for this insightful comment. Certainly, I think it would be more appropriate to use "additive" rather than "synergistic". We deleted "synergistic" in the text.

We show the transcript levels of *KRP5/ICK3* in the *top1a* mutant in **Fig. 4C.** The transcript levels were significantly higher in the *top1a* mutant than in the wild type, suggesting that *TOP1a* represses *ETT/ARF* and *KRP5/ICK3* expression (Fig. 7C).

© 2026. Published by The Company of Biologists under the terms of the Creative Commons Attribution License (https://creativecommons.org/licenses/by/4.0/).

Fig. 4C

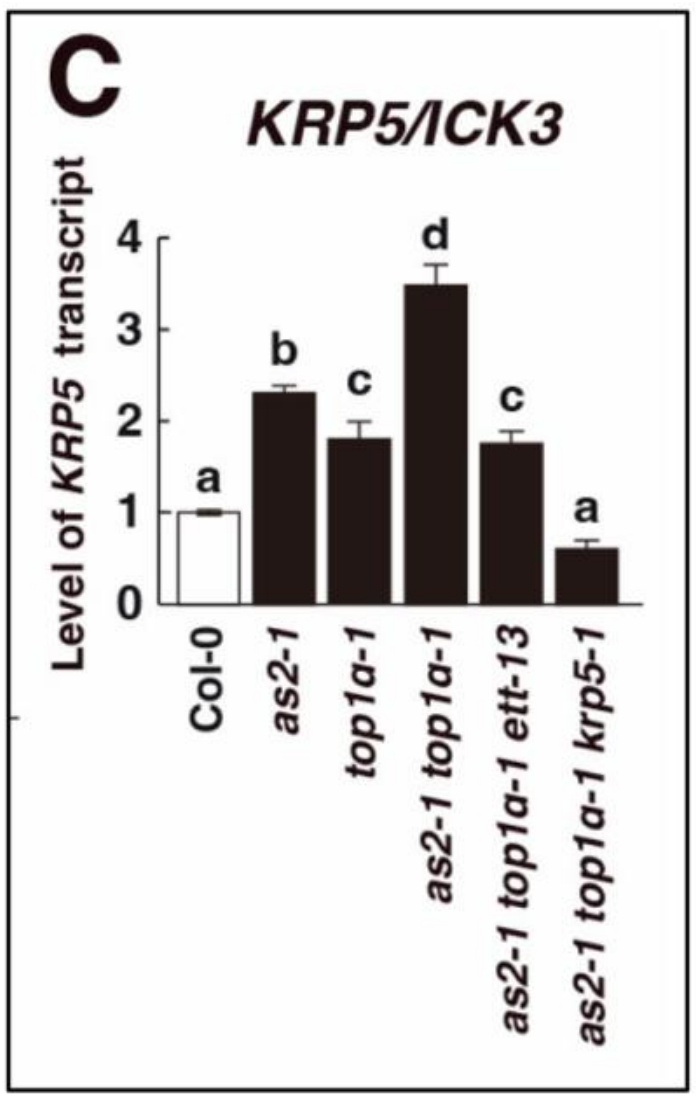

The final model shown in Fig. 7C shows that TOP1*α* regulation is mediated by AS2 bodies, and that AS2 and TOP1*α* regulate ETT-KRP5 independently. However, to avoid such misunderstandings, I believe the final model shown in Fig. 7C needs to be revised.

Fig. 7C

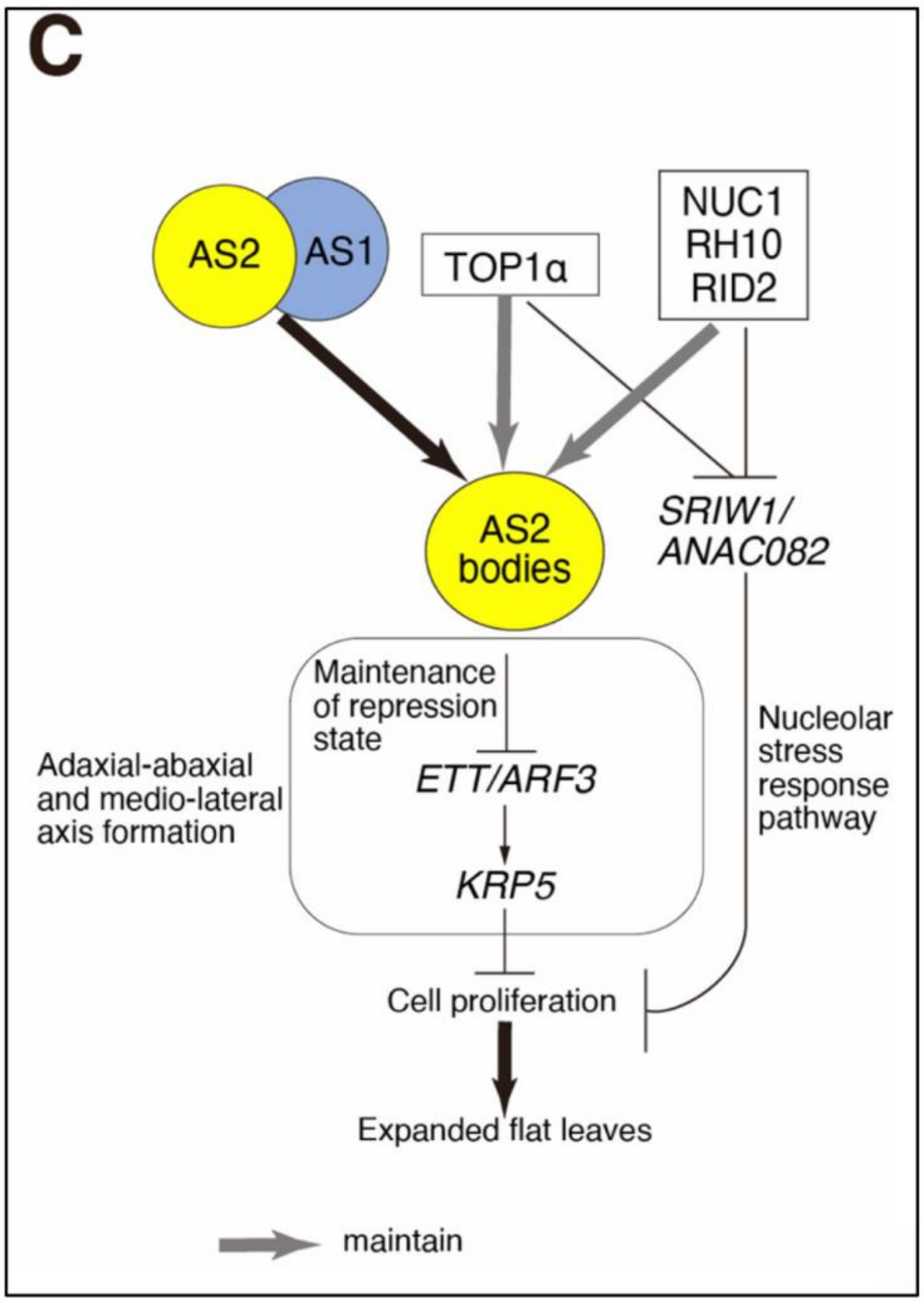

4. The descriptions related to Figure 3, S3, and the corresponding supplemental Tables need to be rewritten. Materials and Methods section completely lacks any explanation of the criteria used for gene expression analysis and clustering analysis. Without this information, the readers cannot understand what kind of gene analysis was performed. Specifically, the gene categorization such as "cell fate" and "cell division was serious problem. How were these categories determined? If GO

© 2026. Published by The Company of Biologists under the terms of the Creative Commons Attribution License (https://creativecommons.org/licenses/by/4.0/).

term enrichment was used, this should be explicitly stated. If an in-house method was employed, it should be disclosed carefully. Particularly since the microarray experiments are now irreproducible, more careful description is essential.

<Response>: We wish to thank the Reviewer for this comment. We apologize that we did not explain KB-FuzzyART.
In accordance with the Reviewer's comment, we added sentences, **Microarray data processing in MATERIALS AND METHODS section, as shown in [Line 666-699].**

Our group have published papers related to KB-FuzzyART as follows.
Tomida et al., *Bioinformatics* 2002; Takahashi et al, *J. Biosci. Bioeng.* 2008; Takahashi et al., *Plant Cell Physiol.* 2013; Iwasaki et al., *Development* 2013; Matsumura et al., *Biol. Open* 2016.

KB-FuzzyART is a knowledge-based clustering method (algorithm) developed in the field of bioinformatics research. Its purpose is to improve the efficiency and accuracy of clustering (grouping) by utilizing existing biological knowledge (such as known genes) when analyzing complex datasets such as gene expression data.

Its main features are as follows: • Use of knowledge: By incorporating known gene information before clustering all the data, the accuracy of the analysis can be improved.

• Applications: It has been applied to research on genes involved in leaf development and differentiation in Arabidopsis thaliana, and to the analysis of gene clusters related to diseases such as cancer. • FuzzyART-based: It is based on a machine learning algorithm called FuzzyART, and can flexibly handle similarities between data. This method is useful for discovering new regulatory networks and unknown candidate genes from large amounts of genetic data.

5. Please examine the ETT and KRP5 expression level in as2 top1α sriw1 triple mutant, to make sure the nucleolus stress response-based leaf phenotype is mediated by the ETT and KRP5 upregulation.

<Response>: We appreciate the reviewer's comment on this point. We examined the level of transcripts of the *ETT/ARF3* and *KRP5/ICK3* genes in *as2 top1a sriw1* triple mutant. We showed these results in **Fig. 6F,G,H,**

[Line 419-425] We next examined *ETT/ARF3*, *KRP5/ICK3*, and *SRIW1/ANAC082* transcript levels in these mutant plants. All three transcripts accumulated to higher levels in the *as2-1 top1a-1* mutant, and these increases were suppressed by the *sriw1* mutation (Fig. 6F-H). *SRIW1/ANAC082* transcript levels were increased in shoot apices of *top1a-1*, and further elevated in the *as2-1 top1a-1* mutant (Fig. 6H). These results suggest that nucleolar stress is induced in the *top1a* mutant.

AS2 may alleviate leaf formation defects under nucleolar stress caused by the *top1a* mutation.

© 2026. Published by The Company of Biologists under the terms of the Creative Commons Attribution License (https://creativecommons.org/licenses/by/4.0/).

Fig. 6F,G,H

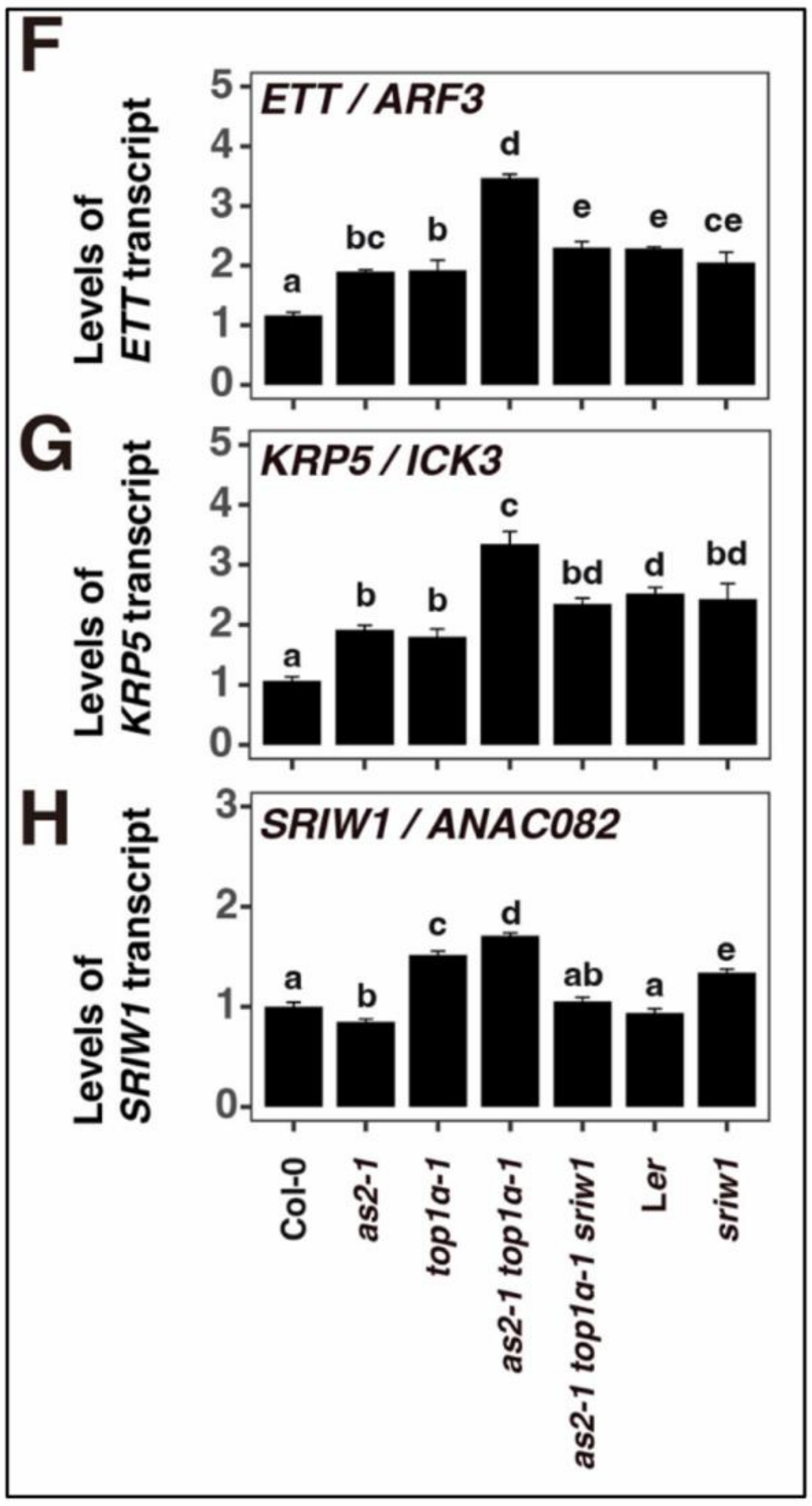

6. Figure 5 and S6: please check the effects of these chemicals on as2 ett and as2 krp5, or more appropriate mutant lines, to conclude "the genome stress affects AS2-ETT-KPR5 pathway," as the authors claimed. In addition,the top2 mutant analysis might be required. However, to be honest, I feel this section is unnecessary for this paper. Although the authors would like to conclude with nucleolar stress, this part discussed the genomic stress (and TopII) instead, leaving it hanging and not incorporated into the model in Fig. 7. The data showing oryzalin nor propyzamide did not affect leaf morphogenesis is very interesting, as it indicates the importance of something beyond simple cell divisional control, therefore it would be reasonable to leave as is.

<Response>:
Etoposide treatment has been shown to induce nucleolar stress in animal cells. Therefore, we hypothesized that etoposide treatment in *Arabidopsis* would similarly trigger nucleolar stress and lead to the formation of rod-shaped leaves in the *as2* and *as1* mutant backgrounds, as observed with 10H-CPT.

Based on these results, we now believe that etoposide, mitomycin C, and bleomycin A5 may also induce nucleolar stress in Arabidopsis, as observed with 10H-CPT. Weexamined the effects of these molecules on the early stages of Arabidopsis leaf development.

Following the reviewer's suggestion, we conducted experiments to examine the leaf phenotype of the *as2 krp5* mutant when etoposide, mitomycin C, and bleomycin A5 are administered.

We showed the results in Fig. 5H,I and Fig. S9C,D,E,F,

[Line 382-388] Etoposide treatment induced filamentous leaves in 30% of *as2-1* plants (**Fig. 5H,I**). The incidence of filamentous leaves was reduced to 10% in the *as2-1 krp5-1* mutant (Fig. 5H,I).

© 2026. Published by The Company of Biologists under the terms of the Creative Commons Attribution License (https://creativecommons.org/licenses/by/4.0/).

Thus, inhibition of TOPI and TOPII was closely associated with filamentous leaf formation and increased *ETT/ARF3* and *KRP5/ICK3* transcript levels.

After treatment with mitomycin C and bleomycin A5, the incidence of filamentous leaves in the *as2-1 krp5-1* mutant was lower mean values than in *as2-1*, although the difference was not statistically significant (**Fig. S9C-F**).

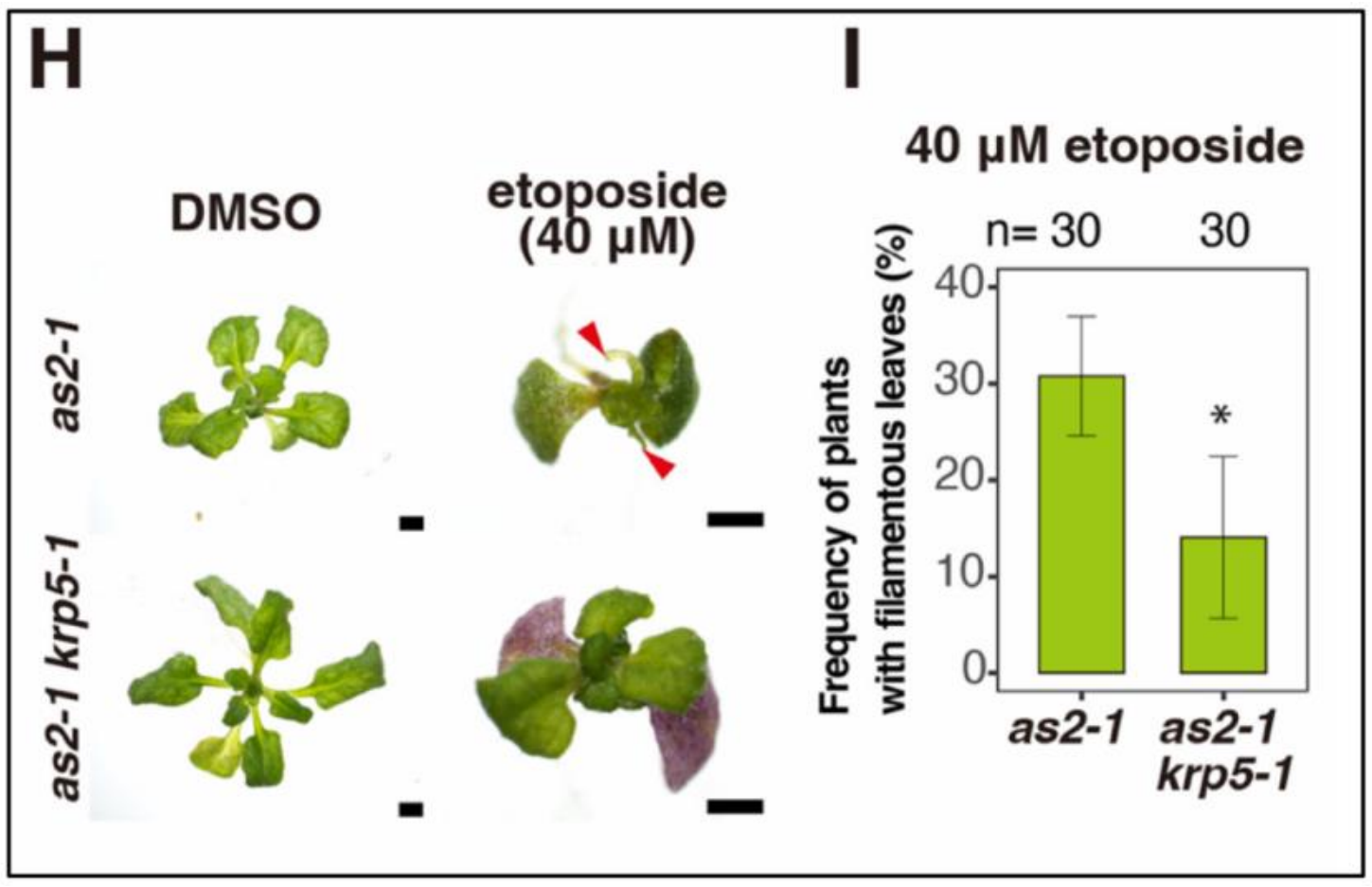

Fig. 5H,I

Fig. S9C,D,E,F

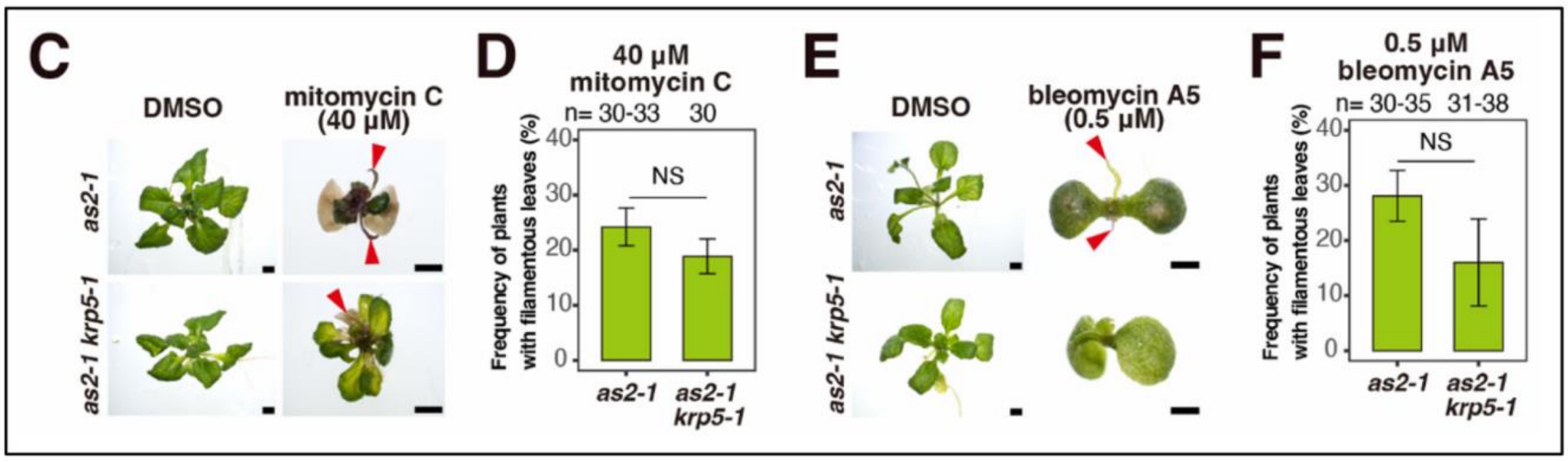

We next investigated whether the localization of AS2 bodies changes when etoposide is administered.
We showed these results in **Fig. 6B.**
[Line 398-400] As shown in Fig. 6A,B, the number of AS2 bodies markedly increased and their morphology became more heterogeneous with increasing concentrations of 10H-CPT and etoposide.

© 2026. Published by The Company of Biologists under the terms of the Creative Commons Attribution License (https://creativecommons.org/licenses/by/4.0/).

Fig. 6A

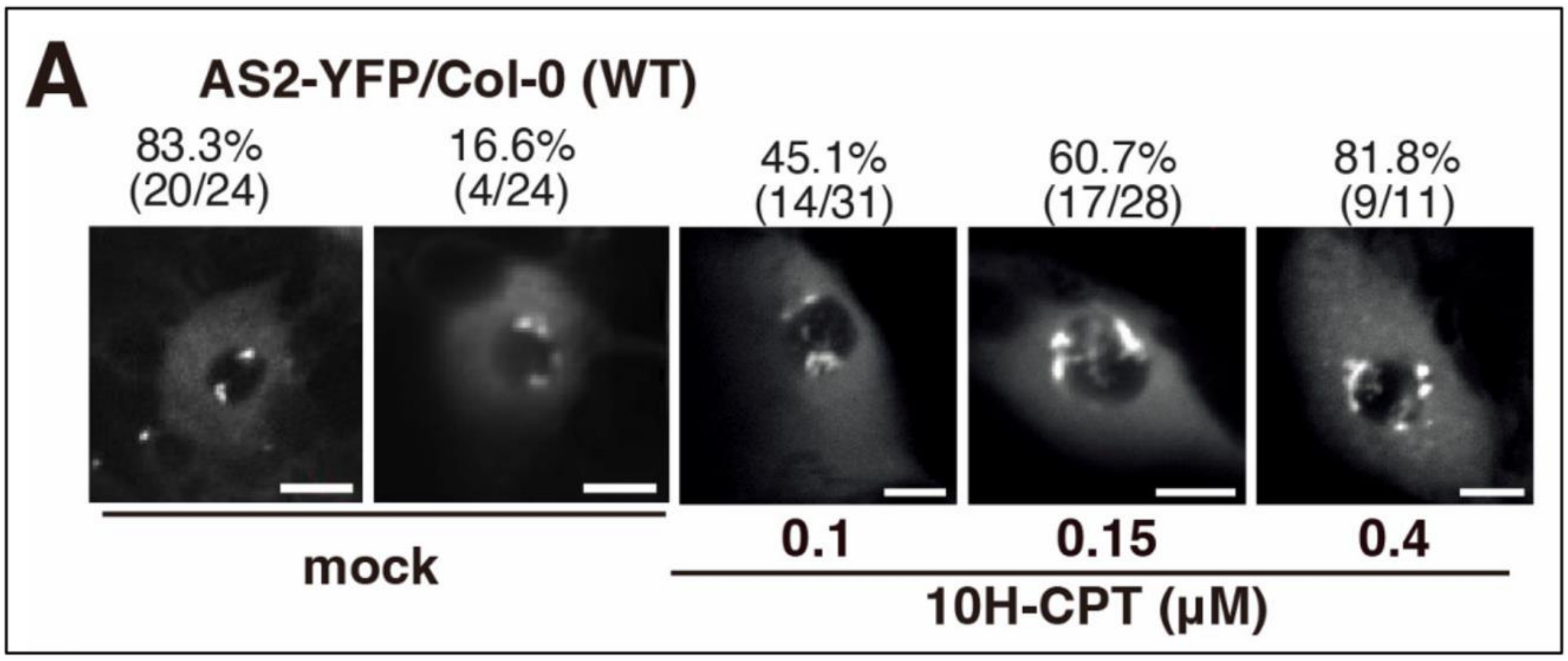

Fig. 6B

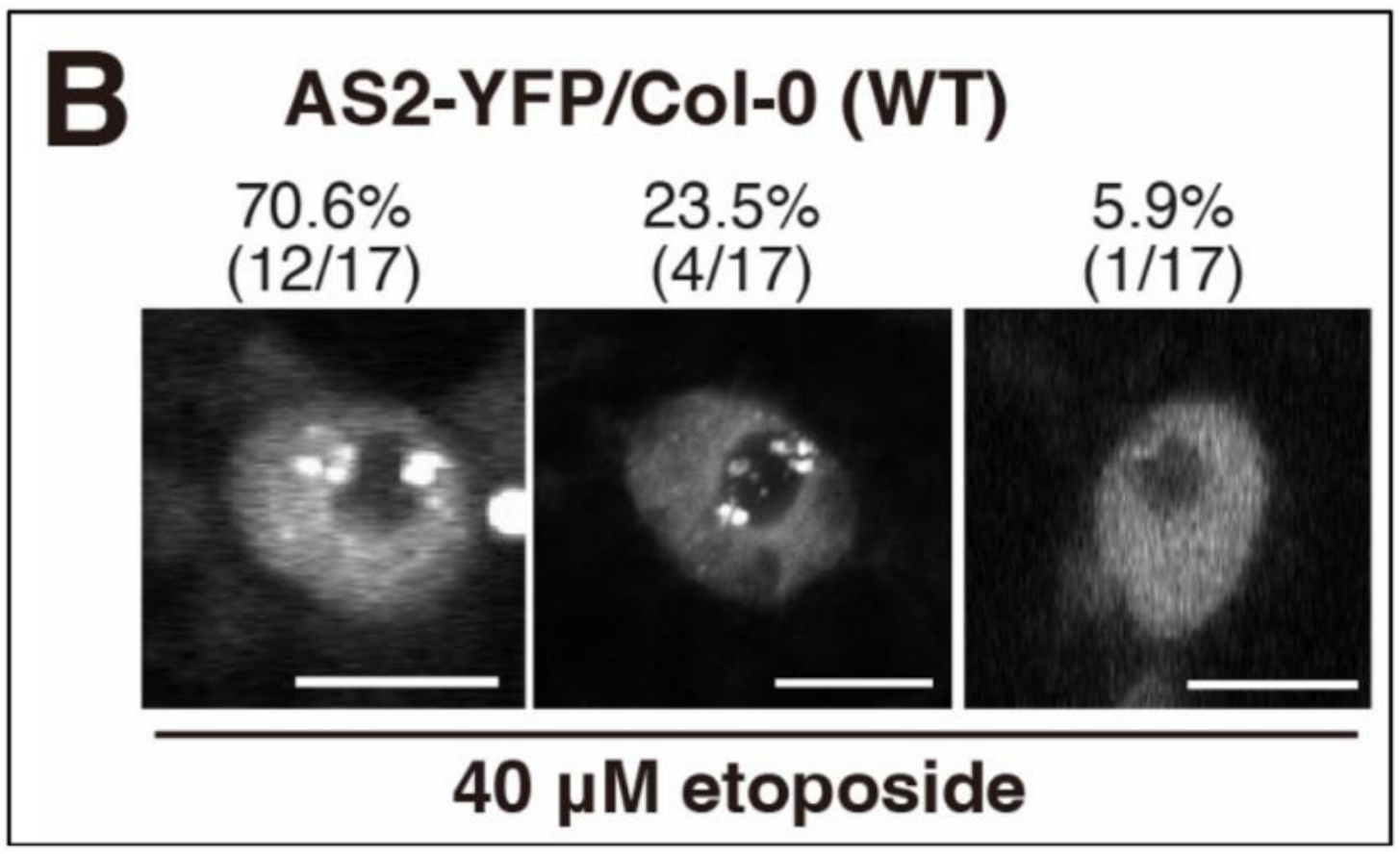

On the other hand, propyzamide, oryzalin, and paclitaxel are inhibitors of microtubule depolymerization during cell division. As shown in **Fig. 5** and **Fig. S9**, the reviewer suggested that the data showing that these substances did not affect AS2-AS1-related leaf morphogenesis might suggest the importance of something beyond simple cell division control. Much remains unknown, and we hope to clarify this further in the future.

Minors

1. For the order of data appearance: Fig. S1A and S1B should be re-named, since Fig. S1B is appeared first.

<Response>: We appreciate the reviewer's comment on this point. We have mistaken the order of data appearance. We re-named Fig. S1A and S1B, as follows.
Fig. S1A and S1B have been corrected (they were previously swapped).

Revised sentences in text [Line 198-201]:
We analyzed GUS activity in filamentous leaves of 10H-CPT-treated *BREVIPEDICELLUS* (*BP*)*p:GUS*/*as2* plants, but GUS activity was not observed (**Fig. S1A**). The *bp-1 knat2-3 knat6-2* mutation did not suppress the filamentous leaf phenotype in 10H-CPT-treated *as2-1* mutants (**Fig. S1B**).

© 2026. Published by The Company of Biologists under the terms of the Creative Commons Attribution License (https://creativecommons.org/licenses/by/4.0/).

Fig. S1A,B

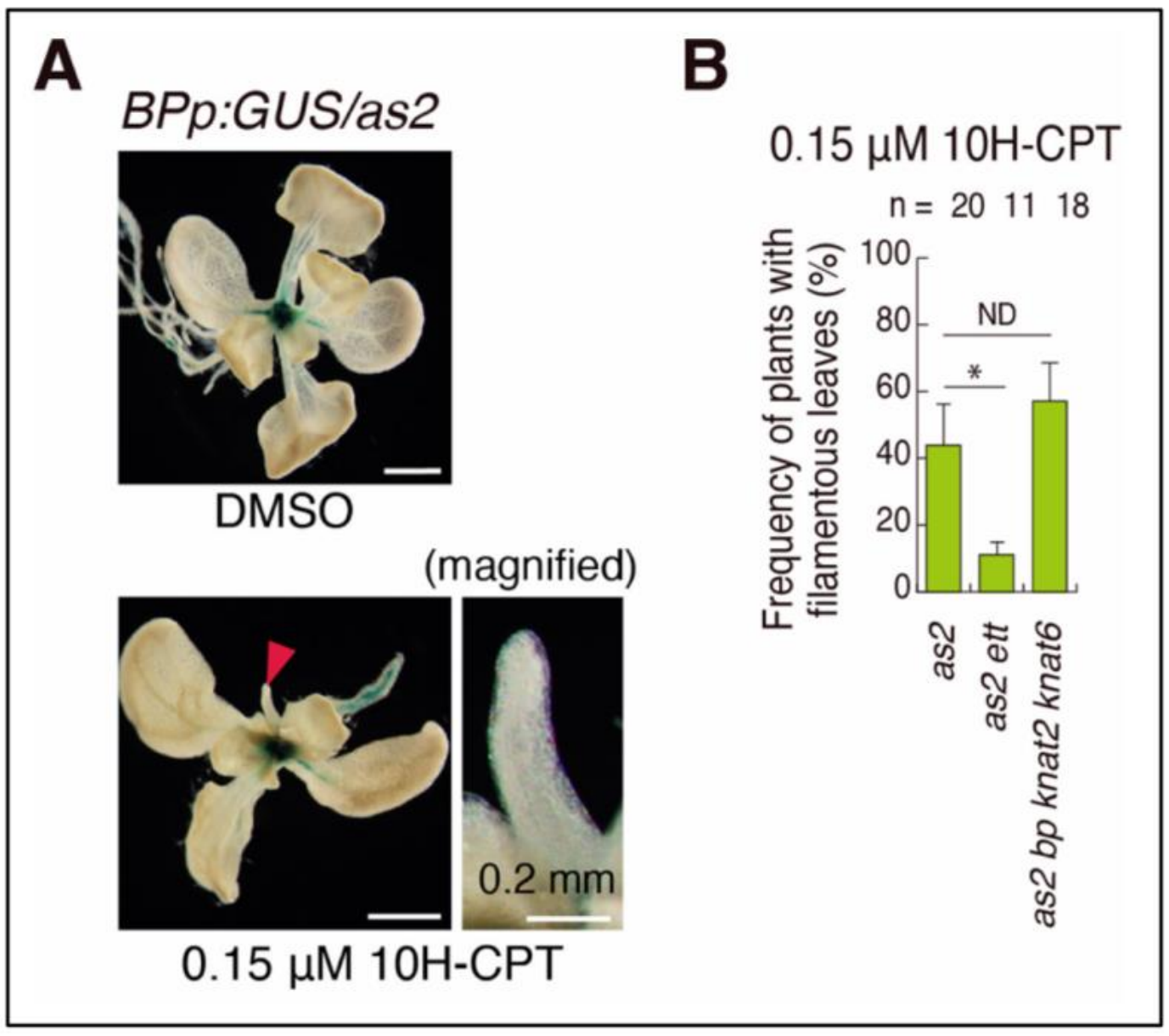

2. Page 9 Line 282 and 294, what means "common"? Please make sure common between what and what.

<Response>: We appreciate the reviewer's comment on this point. We removed "common" as follows:
Original sentence (Line 282):
we identified genes common to 57 candidate genes regulated by both *BOB1/EAL* and *ELO3/EAST1*, which have been shown to act as modifiers of *as2-1* and *as1-1*.

Revised sentence [Line 281-283]:

We extracted 57 overlapping genes co-regulated by both *BOB1/EAL* and *ELO3/EAST1* (Fig. 3A; Kojima et al., 2011; Ishibashi et al., 2012; Takahashi et al., 2013).

Original sentences (Line 294):

The transcription levels of the other six KRP genes did not increase in as2-1 treated with 10H-CPT, indicating that KRP5/ICK3 is the only common downstream gene among the KRP genes.

Revised sentence [Line 290-294]:

The level of *KRP5/ICK3* transcripts was increased in 10H-CPT-treated *as2-1* plants (Fig. S3E). The transcription levels of the other six *KRP* genes did not increase in the *as2-1* mutant treated with 10H-CPT, indicating that *KRP5/ICK3* is the only downstream gene within this family (Fig. S3E, Fig. S3F, Fig. S7B).

3. Line 381: "Cell biology with the recombinant…" should be "Cell biological analysis with the recombinant…". "Cell biology" is too huge to describe your own analysis with AS2, I feel.
Original sentences (Line 381): Cell biology with the recombinant AS2-fused YFP protein (yellow fluorescent protein: AS2-YFP) has shown that the AS2 protein mostly appears as two speckle-like structures at periphery of nucleolus, named AS2 bodies.

<Response>: We appreciate the reviewer's comment on this point. We understand that the term *cell biology* is inappropriate in this context. Therefore, we have revised it as follows.

© 2026. Published by The Company of Biologists under the terms of the Creative Commons Attribution License (https://creativecommons.org/licenses/by/4.0/).

Revised sentence [Line 392-395]: Fluorescence microscopy using the recombinant AS2-YFP fusion protein has shown that the AS2-YFP signal typically appears as two speckle-like structures, termed AS2 bodies, at the periphery of the nucleolus (Ueno et al., 2007; Luo et al., 2012, 2020; Ando et al., 2023).~~;~~

4. Line 489 and other places: "AS2 is a guardian angel" this expression is not acceptable for me. This is far too abstract and religious for a scientific paper. Especially since the as2 ko mutant is viable, thus AS2 is not an essential gene. In this sense, the term "guardian angel" is highly misleading. Please reconsider your phrasing.

<Response>: We appreciate the reviewer's insightful comment. We agree that the phrase "a guardian angel" is not appropriate for a scientific paper.
We deleted the sentence.

5. Related to above comment: the final section of Discussion part (line 489-557) contains many repetitions of results and introductions to past findings, and I believe it lacks substantive messages relevant to the results of this paper. Therefore, it should be made much shorter.

<Response>: We thank the Reviewer for this insightful comment. We intend to rewrite the final section of the Discussion to eliminate the repetition of the results and to make it a more appropriate short sentence that contains a substantive message related to the results of this paper.

AS shown in [Line 429-473], we deleted the 3rd session in DISCUSSION and combined to the 1st session.

6. Line 288-289 "10 genes encoding proteins with known domains such as ETT/ARF3 an BP", I could not understand the meaning of this phrase. Please reword for easy understanding.

<Response>: We appreciate the Reviewer's comment on this expression. We changed sentence as follows.

[Line 287-290] The group of 18 selected genes included 10 genes encoding proteins with **well-characterized** domains, such as *ETT/ARF3* and *BP*, among which *KRP5/ICK3*, a Cip/Kip family CDK inhibitor gene, was included as a gene in the cluster of 'division-related genes'

7. Figure legends for Fig. 1D, 2A,C,D, Fig. 6 (and so on): The author must clearly state what the percentage represents.

<Response>: We appreciate the Reviewer's comment on this point. In accordance with the Reviewer's comment, we added explanation of the state of percentage in Figure legends for **Fig. 1D, Fig. 2A,C,D, Fig. 6A,B,C,D,E.**
Figure legends for Fig. 1 [Line1168-1171] Values above each image indicate the frequency (%) of each leaf phenotype at each concentration (0, 0.2, or 0.5 μM). Numbers in parentheses represent the number of seedlings with the indicated phenotype/total number of seedlings examined.

Figure legends for Fig. 2 [Line1184-1187]; [Line1192-1194]; [Line1198-1201] Values above each image indicate the frequency (%) of each leaf phenotype. Numbers in parentheses represent the number of seedlings with the indicated phenotype/total number of seedlings examined.

Figure legends for Fig. 6 [Line1273-1274] Numbers above each photo show numbers of cells with

© 2026. Published by The Company of Biologists under the terms of the Creative Commons Attribution License (https://creativecommons.org/licenses/by/4.0/).

AS2 body patterning exemplified by the photo/numbers of cells examined.

Second decision letter

MS ID#: dev.205275R1

MS TITLE: Arabidopsis epigenetic factor AS2 mitigates nucleolar stress and promotes flat leaf development by repressing a CDK inhibitor

AUTHORS: Ayami Nakagawa; Hidekazu Iwakawa; Hiro Takahashi; Simon Vial-Pradel; Mari Takahashi; Kazuomi Ohga; Takuma Ito; Motoki Tamai; Eri Takada; Sumie Keta; Nanako Ishibashi; Sayuri Ando; Iwai Ohbayashi; Masaki Ito; Mami Yamazaki; Ayato Sato; Masaaki Umeda; Byung-Yoon Cha; Je-Tae Woo; Michiko Sasabe; Munetaka Sugiyama; Shoko Kojima; Yasunori Machida; Chiyoko Machida

Dear Dr Machida,

I am happy to tell you that your manuscript has been accepted for publication in Development, pending our standard publication integrity checks. It was accepted on 24 Jul 2026.

Reviewer 1

SUMMARY OF THE ADVANCE MADE IN THIS PAPER AND ITS POTENTIAL SIGNIFICANCE TO THE FIELD

The paper demonstrates nucleolar integrity, mediated by AS2, is essential for leaf adaxial-abaxial development.

SUGGESTIONS TO AUTHORS

The author have sufficiently addressed all my reviewer comments.

© 2026. Published by The Company of Biologists under the terms of the Creative Commons Attribution License (https://creativecommons.org/licenses/by/4.0/).
